# Antagonistic fungal enterotoxins intersect at multiple levels with host innate immune defences

**DOI:** 10.1371/journal.pgen.1009600

**Published:** 2021-06-24

**Authors:** Xing Zhang, Benjamin W. Harding, Dina Aggad, Damien Courtine, Jia-Xuan Chen, Nathalie Pujol, Jonathan J. Ewbank

**Affiliations:** 1 Aix Marseille Univ, CNRS, INSERM, CIML, Turing Centre for Living Systems, Marseille, France; 2 Institute of Molecular Biology, Mainz, Germany; The University of Texas Health Science Center at Houston, UNITED STATES

## Abstract

Animals and plants need to defend themselves from pathogen attack. Their defences drive innovation in virulence mechanisms, leading to never-ending cycles of co-evolution in both hosts and pathogens. A full understanding of host immunity therefore requires examination of pathogen virulence strategies. Here, we take advantage of the well-studied innate immune system of *Caenorhabditis elegans* to dissect the action of two virulence factors from its natural fungal pathogen *Drechmeria coniospora*. We show that these two enterotoxins have strikingly different effects when expressed individually in the nematode epidermis. One is able to interfere with diverse aspects of host cell biology, altering vesicle trafficking and preventing the key STAT-like transcription factor STA-2 from activating defensive antimicrobial peptide gene expression. The second increases STA-2 levels in the nucleus, modifies the nucleolus, and, potentially as a consequence of a host surveillance mechanism, causes increased defence gene expression. Our results highlight the remarkably complex and potentially antagonistic mechanisms that come into play in the interaction between co-evolved hosts and pathogens.

## Author summary

When a pathogenic fungus invades an animal, it can deploy a bewildering battery of molecular weapons: hundreds of proteins are injected directly into the host’s cells to enable the fungus to grow and reproduce. We study a simple animal host, the nematode worm *C*. *elegans* and its natural enemy, the fungus *Drechmeria coniospora*, which has a large repertoire of uncharacterised potential “virulence factors”. Here, we focused on just two of these fungal proteins, called enterotoxins. By producing each enterotoxin inside the epidermis of *C*. *elegans*, we were able to study their specific effect on the host, and in particular on the way in which they altered the host’s immune defences. The two enterotoxins had strikingly different effects. One blocked the worms’ ability to make protective antimicrobial peptides, while the other actually stimulated their production. By combining genetics, biochemistry and cell biology, we uncovered the basis of these antagonistic actions. Each of the enterotoxins turned out to act by altering different aspects of the worms’ biology, despite both interfering with host protein translation. Our findings provide a first insight into the molecular and cellular basis of enterotoxins in this particular host-pathogen battle.

## Introduction

The co-evolution of host and pathogen species can be interpreted as a constant arms race, with rounds of reciprocal adaptations driving diversification and divergence on the molecular and macroscopic scales [1]. Thus, an examination of the virulence strategies of a host’s natural pathogens can aid understanding the origin and function of innate immune mechanisms. Our studies focus on the interaction between the nematode *Caenorhabditis elegans* and its natural fungal pathogen *Drechmeria coniospora*. Having dissected in considerable detail *C*. *elegans* defences, we are now also addressing the biology of *D*. *coniospora*.

*In silico* analysis of the fungal genome revealed a large number of genes and gene families potentially involved in virulence [2]. Several approaches exist to address the role of such candidate virulence factors. For example, the corresponding gene could be knocked out in the *D*. *coniospora* genome, and the effect on virulence measured. Alternatively, the endogenous fungal gene could be engineered so that the resultant protein is tagged to allow its visualisation during infection and/or for the use of biochemistry to identify host protein targets. Both these strategies have been applied to *D*. *coniospora* [2]. For unknown reasons, however, the protocol we established for transformation of *D*. *coniospora* [3] no longer functions. This means that we are currently not able to generate mutants in specific *D*. *coniospora* genes. In order to study candidate virulence factors, we therefore adopted a strategy to express the corresponding genes directly in *C*. *elegans*. In a proof of principal, the Falkow laboratory had previously used such a method of heterologous expression, transforming worms with a gene encoding the catalytic subunit of pertussis toxin (PTX) from *Bordetella pertussis*, to find the exotoxin’s conserved target [4]. More recently, we showed that expressing the *Shigella* virulence factor OspF (a MAP kinase inhibitor) in the epidermis of *C*. *elegans* blocked the induction of the antimicrobial (AMP) gene *nlp-29* [5], one of the hallmarks of the innate immune response to *D*. *coniospora* infection [6]. Given the toxic nature of some virulence factors, we refined the method to allow tight control of transgene expression, specifically in the adult epidermis of *C*. *elegans*.

Heat-labile enterotoxins are common and potent virulence factors secreted by pathogenic bacteria. They include cholera and pertussis toxins. They have an αβ_5_ structure, where α is the enzymatically active subunit and the β subunits correspond to the receptor-binding moiety. After the β subunits bind to their specific cell receptor, the toxin is transported into the host cell’s cytosol by endocytosis. The α subunit is then cleaved into α1 and α2 fragments; α1 possesses ADP-ribosylation activity and will modify specific host protein targets [7]. In common with other fungi, *D*. *coniospora* has no genes encoding enterotoxin β subunits, but does have an unusually large number of genes for enterotoxin α subunit proteins [8]. Most of these predicted proteins with a “heat-labile enterotoxin alpha chain” (PFAM: PF01375) domain also have a signal peptide [2] and are expected to be secreted directly into the host cytoplasm, where they could act as toxins, ADP-ribosylating specific targets. The others, lacking a signal peptide, may represent examples of intracellular toxins with a potential role in defence against nematode predation (see [9]). Here, we focused on representative secreted heat-labile enterotoxins and explored the consequences of their expression in *C*. *elegans* to understand better how *D*. *coniospora* is able to infect and harm its host.

## Methods

### *C*. *elegans* culture

Strains used in this study, including SJL1, a kind gift from Adam Antebi, are listed in the S1 Table. All strains were maintained on nematode growth media (NGM) and fed *E*. *coli* strain OP50 [10]. Hygromycin-resistant transgenic worms were maintained on OP50-seeded NGM plates containing 0.2 mg/ml hygromycin B (Thermo Fisher). When large populations of aged-matched worms of such strains were required, young adult worms were bleached by standard alkaline hypochlorite treatment [10] and eggs allowed to hatch overnight in 50 mM NaCl with 0.2 mg/ml hygromycin. L1 larvae were washed three times in 50 mM NaCl to remove hygromycin and grown on standard OP50-seeded NGM plates. This proved an efficient way to select for transgenics, while having a minimal effect on worm physiology, as judged by assaying the expression of *irg-1* (S1 Fig). Otherwise, to perform assays requiring moderate numbers of worms, including for confocal microscopy, young adult transgenic worms were transferred from hygromycin-supplemented onto standard NGM plates and their transgenic progeny selected manually on the basis of fluorescent marker gene expression, to avoid the potentially confounding effects of hygromycin (S1 Fig). In such cases, siblings without fluorescent marker gene expression were also picked to be used as controls.

### *D*. *coniospora* culture and infection

*D*. *coniospora* (Swe3, derived from ATCC 96282 [11]) spores were amplified by infecting worms every one or two weeks in the lab. The method to grow spores is described in detail in [12]. Briefly, sterile 50 mM NaCl was added to plates containing infected worms. A sterile glass microspreader or an L-shaped Pasteur pipette was used to scrape spores gently from the agar surface until the solution became turbid. About 300 μl of the freshly harvested spore solution was then added to a standard 10 cm OP50 plate with 1000–2000 synchronized L4 or young adult worms. The plate was dried in a laminar flow hood and incubated at 25°C for 1 day. Infected worms were harvested with 50 mM NaCl and transferred to an NGM plate supplemented with 100 μg/ml gentamicin and 100 μg/ml ampicillin. The plate was incubated at 25°C for 1 week. Spores were then harvested as above. For assays requiring infected worms, synchronized young adult worms obtained either following treatment with an alkaline hypochlorite solution or using an egg-laying window, were infected by adding 100 μl of a fresh spore solution to a 4 cm OP50 plate (or 200 μl spore solution to a 6 cm OP50 plate). Plates were dried briefly under a hood and then incubated at 25°C.

### Plasmid construction

*D*. *coniospora* (Swe3) cDNA was generated as previously described [2] and purified by QIAquick PCR Purification Kit (Qiagen, Cat No./ID: 28104). The fungal gene RJ55_04834/g4535, without the 5’ sequence corresponding to the predicted signal peptide, with an engineered ATG, and without the stop codon, was amplified from cDNA by PCR with Gibson assembly 5’ and 3’ primers that included *NotI* and *ClaI* sites, respectively (S2 Table). FLAG and tev sequences were synthesised by Integrated DNA Technologies (Leuven, Belgium). mKate2 was amplified from the plasmid pNP152 [13] with Gibson assembly primers. The vector backbone was amplified by PCR using the destination vector pSX103 that contains the promoter of *col-19* [14], a generous gift from Andrew Chisholm, as the template. All the fragments were assembled using the Gibson assembly protocol [15] to give pZX12. The degron sequence was amplified from DNA extracted from the worm strain PX627 [16] and inserted into *AscI*-digested pZX12 using Gibson assembly to give pZX17. To make other plasmids, pZX17 was double digested by *NotI* and *ClaI*, and the RJ55_04834/g4535 gene fragment replaced with the appropriate alternative *D*. *coniospora* gene fragment, generated as described above. *rps-0p*::hygR was amplified from pSO5.3 [5] and inserted into the pSX103 vector (double digested with *KpnI* and *NarI*) using Gibson assembly to give the pZX13 plasmid.

### Transgenic strains

Transgenic strains were obtained by microinjection of 20 ng/μl of each virulence factor construct, 20 ng/μl *rps-0p*::hygR and the coinjection marker *unc-122p*::GFP, a kind gift from Jean-Louis Bessereau, at a concentration of 40 ng/μl into JDW141 (*eft-3p*::TIR::P2A:::BFP-NLS-degron::*tbb-2* 3’UTR). This strain, for use with the auxin-inducible degron system, with an internal degradation control [17], was a generous gift of Jordan Ward. To generate the control hygR;*fr*Is7 strain (IG1864), 60 ng/μl *rps-0p*::hygR and 60 ng/μl *unc-122p*::GFP were microinjected into IG274 (+;*frIs7*) worms that contain integrated *nlp-29p*::*GFP* and *col-12p*::*dsRed* reporter transgenes [18]. All other strains were obtained by conventional crosses using various reporter strains (S1 Table).

### RNA extraction, reverse transcription and quantitative PCR

Worms were harvested and washed three times with 50 mM NaCl and pelleted by centrifugation before Trizol extraction (Thermo Fisher Scientific) following the manufacturer’s instructions. Reverse transcription was performed using High-Capacity cDNA Reverse Transcription Kit (Invitrogen). Quantitative real-time PCR was performed as described [19] by using SYBR Green PCR Master Mix (TaKaRa). Values were normalized to those of *act-1* and were analyzed by the cycling threshold method using the appropriate qRT-PCR primers (S2 Table). Control and experimental conditions were tested in the same run.

### Microscopy and image analysis

Worms were picked into a drop of 0.25 mM levamisole on a 2% agarose pad on a glass slide and observed using a Leica DMRBE microscope. Fluorescent images were taken with a Zeiss AxioCam HR digital colour camera and Axio-Vision Rel. 4.6 software (Carl Zeiss AG). Confocal microscopy used a Zeiss LSM 780. All image processing was done using Fiji software [20]. Comparative analyses were performed with image sets acquired on same day with the same settings on age-matched worms. The area and Feret’s (caliper) diameter for lateral hyp7 nucleoli were measured with an automatic particle analysis method. Feret’s diameter represents the longest distance between any two points along an object’s boundary. For better information extraction, minimizing the background noise and to avoid over and under estimation, automatic thresholding was applied to images of each nucleolus, selected after pseudo-colouring based on pixel intensity and smoothing to discriminate better the area of interest. For any data that did not have a normal distribution (determined with a Shapiro-Wilk test), statistical significance was determined using a nonparametric Mann Whitney test (GraphPad Prism software).

### Analyses with the Biosort worm sorter

Fluorescent protein expression of reporter strains was quantified with the COPAS (Complex Object Parametric Analyzer and Sorter) Biosort system (Union Biometrica; Holliston, MA) as described [18]. For each strain, a minimum of 150 synchronized young adult worms were analyzed for length (assessed as TOF, time of flight), optical density (assessed as extinction) and Green and/or Red fluorescence (GFP/Red). Raw data were filtered on the TOF for adult worms (typically 300 ≤ TOF ≤ 1500). Statistical significance was determined using a non-parametric analysis of variance with a Dunn’s test (GraphPad Prism).

### RNA interference

RNAi bacterial clones were obtained from the Ahringer or Vidal libraries [21,22] and checked by sequencing. RNAi bacteria were seeded on NGM plates with the appropriate antibiotics. Worms were transferred onto RNAi plates as L1 larvae and cultured at 20°C or 25°C as indicated.

### Lifespan

L4 worms were manually transferred to small (4 cm) plates containing NGM agar seeded with *E*. *coli* OP50. Typically, for each strain, 5 plates of 10 worms were assayed. Worms were grown at 25°C and the surviving and dead worms were counted every day. Worms were transferred to new plates every day at the start of the experiment to eliminate the larvae of the next generation. Once worms had stopped producing viable eggs, they were kept on the same plates and the worms that no longer responded to light touch were picked out and scored as dead.

### Survival upon *D*. *coniospora* infection

L4 or young adult worms were manually transferred to 4 cm plates containing NGM agar seeded with *E*. *coli* OP50. Fresh spores were harvested and spread on the plate. Normally, 1 x 10^8^ spores were used for infecting about 100 worms on a 4 cm OP50 plate. After infection (either 8 hours or overnight), for each experimental condition, 4 wells of 25 worms were assayed in a 12-well plate containing NGM agar seeded with *E*. *coli* OP50. Images of each well were collected automatically every 24 minutes using a custom system that will be described elsewhere. The images were examined, and worms scored as dead when they no longer exhibited movement between successive images.

### Cuticle fragility test

The cuticle fragility was tested by measuring the time to cuticle rupture in bleach as previously described [23].

### Cycloheximide (CHX) treatment

CHX (40 mM in DMSO) was added to OP50-seeded NGM agar plates to a final concentration of 1.78 mM (500 μg/ml) and allowed to dry before use. Young adult worms that had been grown at 20°C on OP50 NGM plates were transferred to the plates with CHX for 6h. While exposure to this concentration of CHX for prolonged periods affects development and animal health [24], we found that adults tolerated this concentration well for short (6 h) periods.

### Preparation of worms for biochemistry

Large quantities of worms were prepared using enriched NGM agar medium (NGM+; 3 g NaCl, 20 g peptone, 25 g agar, 1 ml of 5 mg/ml cholesterol (in ethanol) in 975 ml of H_2_0 autoclaved, cooled then supplemented with 1 ml of 1 M CaCl_2_, 1 ml of 1 M MgSO_4_, 25 ml of 1 M Phosphate buffer, 1 mL of 100 mg/mL ampicillin), seeded with 10x concentrated HT115 *sta-1*(RNAi) clone, and grown for 20–24 h at 37°C to obtain a thick bacterial lawn. During the amplification of strains, to select transgenic worms, seeded plates were supplemented with hygromycin at a final concentration of 0.2 mg/ml. In the case of worms expressing DcEntA and DcEntB, plates were additionally supplemented with auxin at a final concentration of 1 mM to limit any potential reduction of fecundity associated with expression of the virulence factors.

Mixed stage worm populations were collected by using 50 mM NaCl, 0.05% Triton X-100 in 15 ml tubes, then washed three times in 50 mM NaCl prior to standard alkaline hypochlorite treatment. The recovered eggs were then washed three times and allowed to hatch overnight in 3 ml of 50 mM NaCl supplemented with 0.2 mg/ml hygromycin, with gentle agitation. The synchronised L1 worms were then added to NGM + plates, and grown at 25°C until they reached the young adult stage. The expression of the chimeric virulence protein was confirmed by the observation of the expected red fluorescence signal using a dissecting fluorescence microscope. Worm samples were then collected by using 50 mM NaCl, 0.05% Triton X-100 in 15 ml tubes and then washed 3–4 times in 50 mM NaCl, until the supernatant was cleared of bacteria, before freezing the worm pellets at -80°C.

### Immunoprecipitation assay

Pellets of synchronised young adult worms (0.8–1 ml) were thawed in the presence of an equal volume of 2x lysis buffer (75 mM Hepes, pH 7.5; 1.5 mM EGTA; 1.5 mM MgCl_2_; 150 mM KCl; 15% glycerol; 0.075% NP-40 to which Roche cOmplete Mini tablets containing a protease inhibitor cocktail were added just prior to use). Thawed worm pellets were then flash frozen in liquid nitrogen and then crushed using a pestle and mortar on dry ice prior to sonication in Diagenode 15 ml tubes (C30010017) using a Diagenode BioRuptor Pico (10 cycles 15 s on; 45 s off) cooled to 4°C. Lysates were then transferred to 2 ml Eppendorf tubes, the concentration of NP-40 adjusted to 0.5% and centrifuged for 10 min at 8000 rpm at 4°C. The supernatant was then transferred in 900 μl aliquots to fresh Eppendorf tubes and incubated with gentle head-over-tail agitation for 2 h at 4°C with 30 μl of anti-RFP or control beads (Chromotek rtma-2 and bmab-20, respectively) that had been washed in 1x lysis buffer. The beads were then washed once in Wash buffer I (25 mM Tris-HCl pH 7.4, 300 mM NaCl, 1 mM MgCl_2_) and twice in Wash buffer II (1 mM Tris-HCl pH 7.4, 150 mM NaCl, 1 mM MgCl_2_) and proteins then released from the beads through incubation in 1x Laemmli buffer at 95°C for 10 minutes.

### Protein in-gel digestion

Proteins were separated briefly in a 4–12% NuPAGE Bis-Tris gel, stained with Coomassie blue and cut into small gel cubes, followed by destaining in 50% ethanol/25 mM ammonium bicarbonate. The proteins were then reduced in 10 mM DTT at 56°C and alkylated by 50 mM iodoacetamide in the dark at room temperature. Afterwards, proteins were digested by trypsin (1 μg per sample) overnight at 37°C. Following peptide extraction through sequential incubation of gel cubes in 30% and 100% acetonitrile, the sample volume was reduced in a centrifugal evaporator (Eppendorf) to remove residual acetonitrile. The resultant peptide solution was purified by solid phase extraction in C18 StageTips [25].

### Liquid chromatography tandem mass spectrometry

Peptides were separated in an in-house packed 50 cm analytical column (inner diameter: 75 μm; ReproSil-Pur 120 C18-AQ 1.9 μm resin, Dr. Maisch GmbH) by online reversed phase chromatography through a 90 min gradient of 2.4–32% acetonitrile with 0.1% formic acid at a nanoflow rate of 250 nl/min. The eluted peptides were sprayed directly by electrospray ionization into an Orbitrap Exploris 480 mass spectrometer (Thermo Scientific). Mass spectrometry measurement was conducted in data-dependent acquisition mode using a top15 method with one full scan (resolution: 60,000, target value: 3 × 10^6^, maximum injection time: 28 ms) followed by 15 fragmentation scans via higher energy collision dissociation (HCD; normalised collision energy: 30%, resolution: 15,000, target value: 1 × 10^5^, maximum injection time: 40 ms, isolation window: 1.4 m/z). Precursor ions of unassigned, +1, +7 or higher charge state were rejected for fragmentation scans. Additionally, precursor ions already isolated for fragmentation were dynamically excluded for 25 s.

### Mass spectrometry data analysis

Raw data files were processed by MaxQuant software package (version 1.6.5.0) [26] using Andromeda search engine [27]. Spectral data were searched against a target-decoy database consisting of the forward and reverse sequences of WormPep release WS275 (28,466 entries), UniProt *E*. *coli* K-12 proteome release 2020_01 (4,403 entries), the corresponding transgenic fusion protein and a list of 246 common contaminants. Trypsin/P specificity was selected. Carbamidomethylation of cysteine was chosen as fixed modification. Oxidation of methionine and acetylation of the protein N-terminus were set as variable modifications. A maximum of 2 missed cleavages were allowed. The minimum peptide length was set to be 7 amino acids. At least one unique peptide was required for each protein group. False discovery rate (FDR) was set to 1% for both peptide and protein identifications. A separate database search was performed in strain DcEntA to identify mono-ADP-ribosylation (MAR; C_15_H_21_N_5_O_13_P_2_, *m/z* 541.0611) sites on residues CDEKNRST as variable modifications and ribose, adenosine, AMP and ADP as diagnostic ions [28].

Protein quantification was performed using the LFQ label-free quantification algorithm [29]. Minimum LFQ ratio count was set to one. Both the unique and razor peptides were used for protein quantification. The “match between runs” option was used for transferring identifications between measurement runs allowing a maximal retention time window of 0.7 min. All mass spectrometry raw data have been deposited to the PRIDE repository [30] with the dataset identifier PXD021929. DcEntA and DcEntB are referenced as pZX26 and pZX25, respectively. This dataset also includes pull-down data for three other structurally-unrelated virulence factors used to identify proteins that bound in a non-specific manner, as described below.

Statistical data analysis was performed using R statistical software. Only proteins quantified in at least two out of the three RFP pull-down replicates were included in the analysis. LFQ intensities were log-transformed. Imputation for missing values was performed for each pull-down replicate by random picking from a normal distribution that simulated low intensity values below the noise level. The LFQ abundance ratio was then calculated for each protein between the RFP pull-downs and the controls. Significance of the enrichment was measured by an independent-sample Student’s *t* test assuming equal variances. Specific interaction partners were then determined in a volcano plot where a combined threshold (hyperbolic curve) was set based on a modified *t*-statistic (SAM, significance analysis of microarrays) [31,32]. Proteins that passed the combined threshold in a second strain (PRIDE repository PXD021929) were considered as potential cross-reactive, non-specific binders and were filtered out from the volcano plot and summary spreadsheets.

### *In silico* analyses

BLASTP searches were conducted at the NCBI on 09/11/20, using default parameters, but excluding *D*. *coniospora* entries. We used the command-line version of NoD (v1.3b) [33] available as a Conda package (https://anaconda.org/bioconda/clinod). In addition to the default parameters, we used “-clean_sequence” as some *D*. *coniospora* proteins in the dataset [2] contain Xs in their sequence. The default output of NoD was parsed using a Python script (enclosed as a Jupyter notebook; available on request). Gene class enrichment analyses used Wormbase release WS277.

## Results

### Enterotoxins have distinct expression patterns and impact physiology when expressed in the epidermis

**Fig 1:**
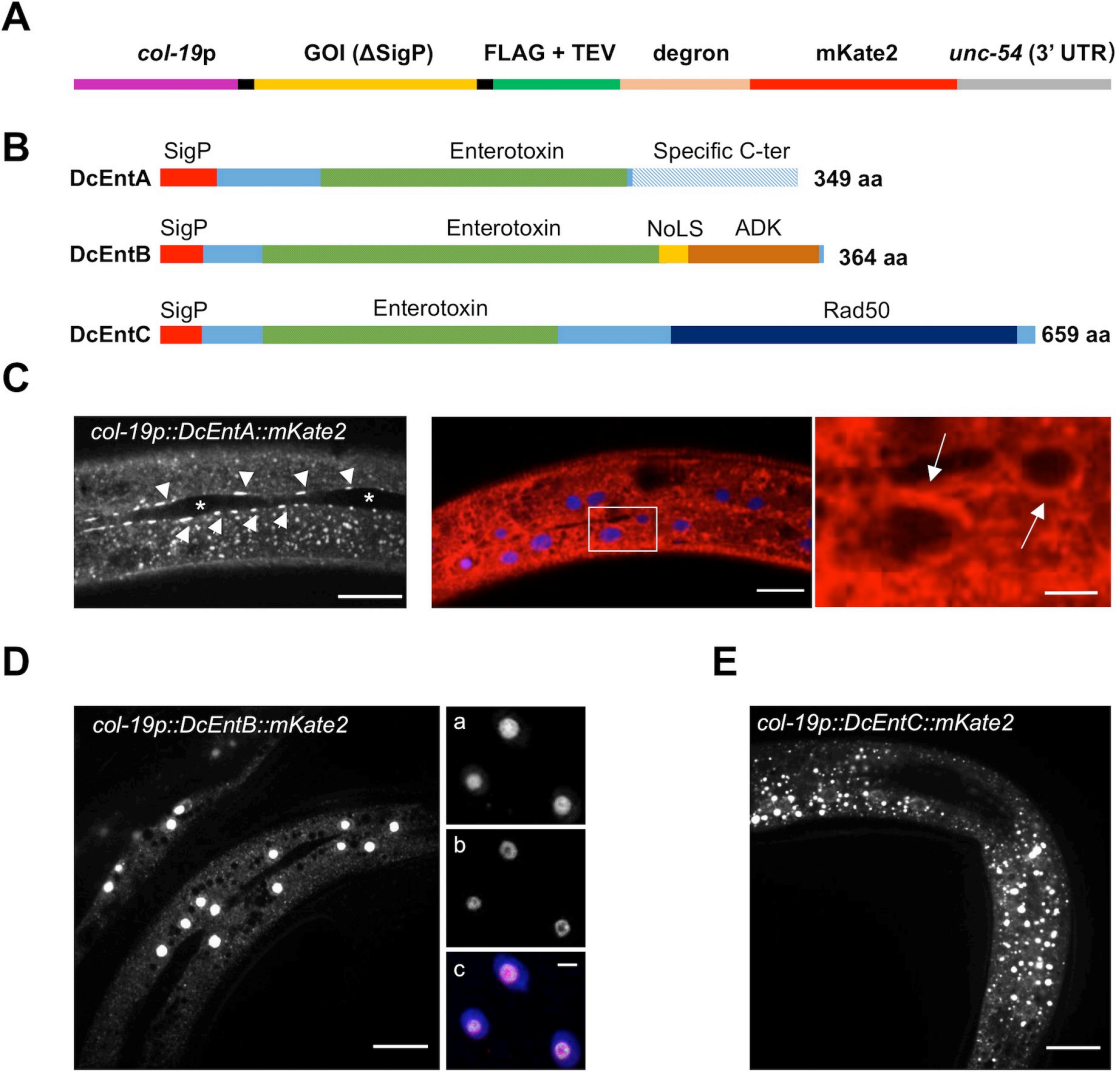
Three enterotoxins have different expression patterns in the epidermis. (**A**) Schematic overview of the plasmid insert used to express virulence factors. The candidate gene of interest (GOI), corresponding to a virulence factor without its signal peptide (ΔSigP) and stop codon was cloned between the *col-19* promoter and *unc-54* 3’ UTR, and expressed as a fusion protein with FLAG, tobacco etch virus (TEV) protease cleavage site, degron and mKate2. (**B**) Schematic overview of three selected enterotoxins from *D*. *coniospora*. The signal peptide (SigP) is represented in red, the heat-labile enterotoxin alpha chain domain (PFAM: PF01375) in green, a *D*. *coniospora* specific 90-residue C-terminal domain in DcEntA (hatched blue), the DcEntB nucleolar targeting sequence (NoLS) predicted by NoD [33] in yellow, the regions similar (p = 9.44e^-3^) to part of an adenylate kinase (ADK) domain (PRK13808), in brown, and DNA double-strand break repair ATPase Rad50 (PRK03918; 4.4e^-5^) in dark blue for DcEntC. The remainder of the proteins’ sequences is in light blue. Representative confocal fluorescence images of young adult worm expressing DcEntA::mKate2 (IG1926; **C**), DcEntB::mKate2 (IG1925; **D**), and DcEntC::mKate2 (IG1880; **E**). (**C**) Left panel: DcEntA (arrowheads) adjacent to seam cells (asterisks). Middle: DcEntA (red) accumulation around nuclei (blue) is highlighted with arrows in the right panel showing a magnified view of the boxed area. Scale bars, left and middle, 20 μm, right, 5 μm. (**D**) Left panel: young adult IG1925 worm expressing DcEntB::mKate2. Scale bar, 20 μm. Right: Image of young adult IG1984 worm expressing DcEntB::mKate2, FIB-1::GFP, and BFP-NLS (a: red channel; b: green channel; c: overlay red, green and blue channels). Scale bar, 5 μm. (**E**) DcEntC::mKate2 appears as a punctate cytoplasmic pattern. Scale bar, 20 μm.

As part of our investigation of the virulence mechanisms of *D*. *coniospora*, we adopted the strategy of expressing individual candidate fungal proteins through transgenesis in *C*. *elegans*. We expressed them under the control of a promoter (*col-19*) that is principally active in the epidermis from the young adult stage onwards [34,35]. In anticipation of potential deleterious effects of residual virulence factor expression during development, we engineered the proteins as chimeric constructs, adding an auxin-inducible degron (AID) [36]. We also included tags for microscopy and biochemistry (mKate2 and Flag, respectively; Fig 1A). We used this approach to address the function of representative members of the large family of enterotoxin α (PF01375) domain proteins. Enterotoxins are well known as important mediators of bacterial virulence [7] and this protein family is expanded in *D*. *coniospora* [8]. Through manual curation, 23 enterotoxin α genes were predicted in the genome of the *D*. *coniospora* strain used here, distributed across all 3 chromosomes, including several clusters of paralogues, compared to 27 in a second strain (ARSEF 6962; S2 Fig). On the basis of their predicted sequence, expression and position in a phylogenetic tree (S3 Fig), we selected three candidates (ODA75893.1/RJ55_08534/g7949, ODA80052.1/RJ55_03010/g2819 and ODA76808.1/RJ55_07324/g6833) that we refer to here as DcEntA, DcEntB and DcEntC, respectively (Fig 1B). The 3 proteins have all the sequence features exhibited by other validated ADP-ribosylating enterotoxins and are presumed to be enzymatically active. When expressed as fusion proteins, each gave a distinct pattern of intracellular localisation in the main epidermal syncytium (hyp7). DcEntA was enriched in the perinuclear region as well as at the cell membrane, adjacent to seam cells (Fig 1C). DcEntB appeared to be restricted to the nucleolus, consistent with the presence of a predicted nucleolus-localisation signal (NoLS) in its primary sequence (Fig 1B and 1D). This was confirmed by its co-localisation with the known nucleolar marker FIB-1::GFP [37] (Fig 1D). DcEntC, on the other hand, gave a punctate, cytoplasmic pattern (Fig 1E). Any strategy of expressing chimeric proteins under the control of a heterologous promoter runs the risk of experimental artefacts. The very distinct patterns seen for the 3 chimeric DcEnt proteins, however, suggests that their localisation was not unduly influenced by the added non-fungal sequences that are common to all 3 proteins.

**Fig 2:**
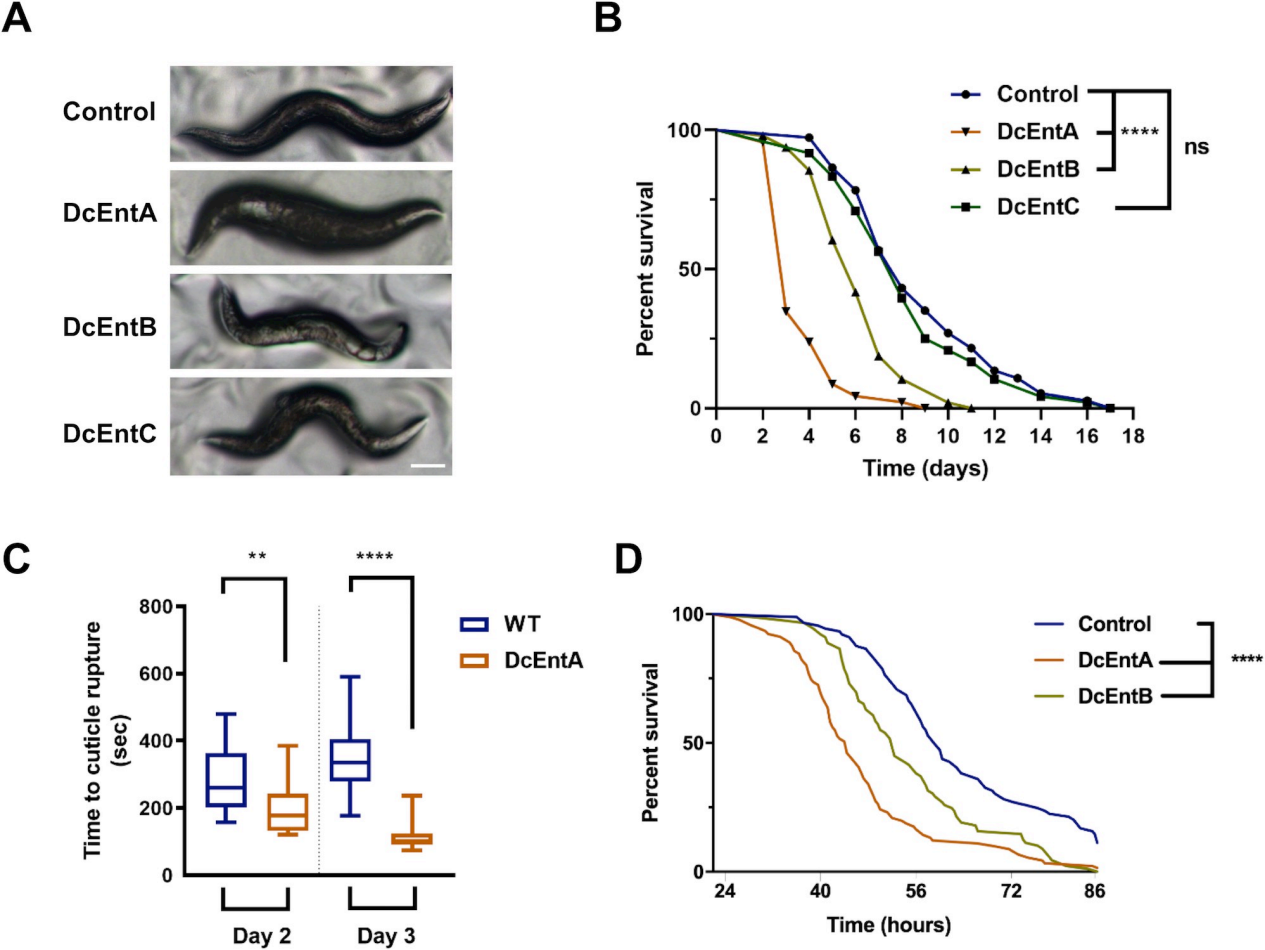
Expression of DcEntA or DcEntB makes worms sick and die precociously. (**A**) Representative images of control and enterotoxin-expressing worms on the third day of adulthood. From the top: control, DcEntA-, DcEntB- and DcEntC-expressing worms (JDW141, IG1926, IG1925 and IG1880, respectively); scale bar, 100 μm. (**B**) Lifespan counted from the L4 stage at 25°C of worms of these 4 strains. For each strain, n = 50. **** p < 0.0001, one-sided log rank test. (**C**) Expression of DcEntA increases cuticle fragility in 2- and 3-day old adult worms. Tukey boxplots (n > 20, for each condition); unpaired t test, ** p < 0.01; **** p < 0.0001. (**D**) Survival of worms carrying *frIs7* [*nlp-29p*::*GFP* and *col-12p*::*dsRed*] and a *hygR* transgene (control; IG1864) or also expressing DcEntA (IG1942) or DcEntB (IG1941) after infection as young adults with *D*. *coniospora* at 25°C (n = 91, 89 and 89 respectively). **** p < 0.0001, one-sided log rank test. Representative of 3 independent biological replicates.

The different strains underwent larval development normally and we were able to maintain them under standard culture conditions. Except when amplifying large populations for biochemistry (see Methods, and below), we therefore conducted experiments on worms that had not been exposed to auxin, avoiding its possible confounding side effects [38]. After the 4^th^ larval stage (L4) to adult moult, coincident with the increasing activity of the *col-19* promoter, fluorescence from the chimeric proteins started to be visible. As the worms aged and virulence factor expression increased (reflected by an increase in fluorescence), the strains expressing DcEntA and DcEntB manifested signs of sickness (Fig 2A and 2B and S3 Table) and the worms were short-lived. Worms expressing DcEntC, however, had more subtle phenotypes, an almost normal morphology and behaviour, and had a lifespan that was indistinguishable from control animals (Fig 2A and 2B and S3 Table). We therefore concentrated principally on the characterisation of DcEntA and DcEntB. Upon normal handling, adult DcEntA-expressing worms, but not DcEntB-expressing worms, appeared more prone to break apart. This was confirmed in the standard test of cuticle fragility, wherein the time to rupture for 2-day old adults expressing DcEntB was significantly shorter than for age-matched control worms (Fig 2C). On the other hand, both the DcEntA- and DcEntB-expressing worms were also significantly more susceptible to infection by *D*. *coniospora* than control worms (Fig 2D). Thus, controlled transgenic expression of certain individual fungal enterotoxins in the epidermis, albeit at an elevated level (S4 Fig), is sufficient to reduce *C*. *elegans* longevity and resistance to infection.

### DcEntA blocks AMP gene expression after infection

**Fig 3:**
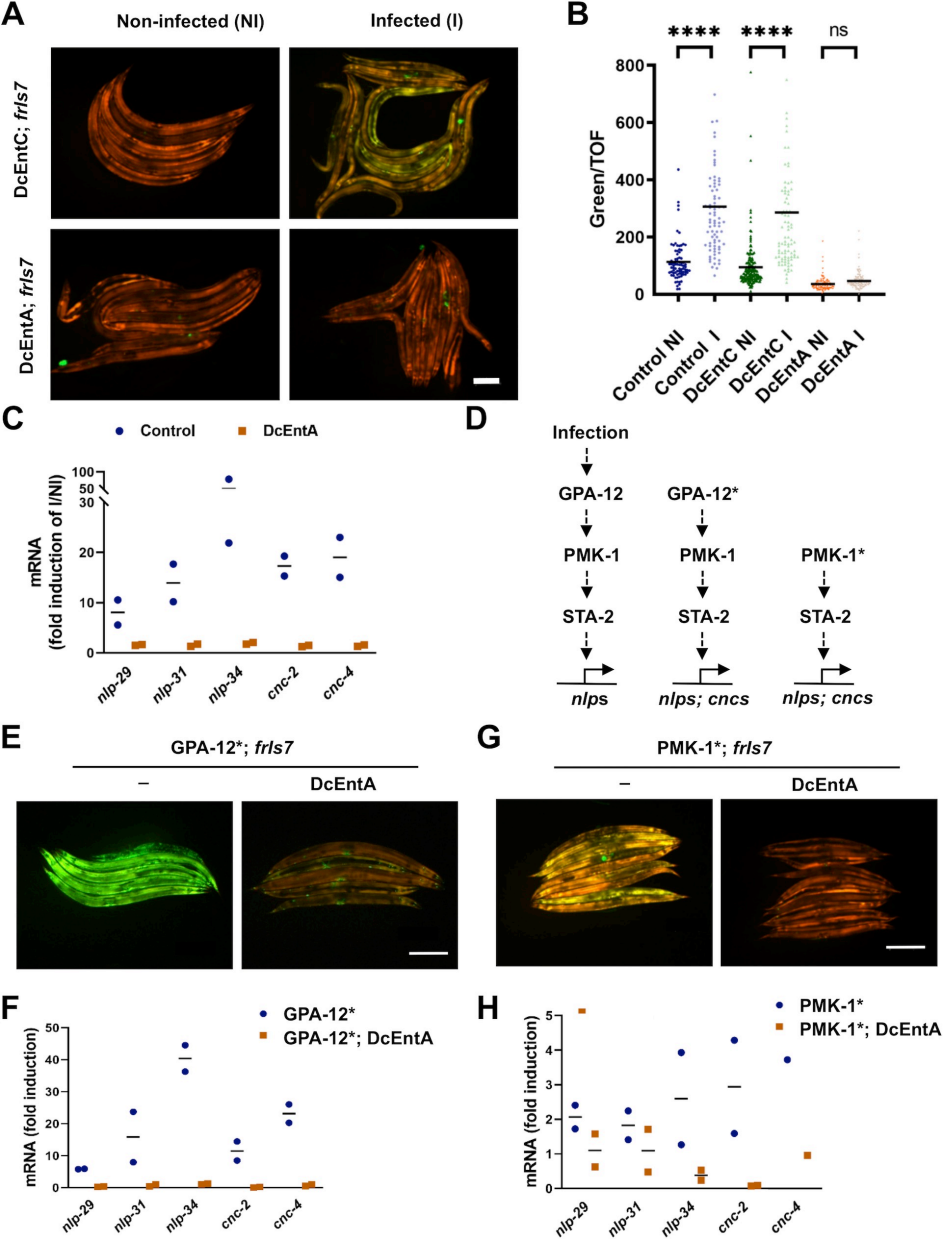
DcEntA blocks AMP gene expression after infection. (**A**) Representative images of DcEntC;*frIs7* (IG1883; upper panels) and DcEntA;*frIs7* (IG1942; lower panels) worms either not infected (left), or 20 h after infection with *D*. *coniospora* (right) as young adults. *frIs7* includes *nlp-29p*::*GFP* and *col-12p*::*dsRed* transgenes; red and green florescence is visualized simultaneously. Scale bar, 200 μm. (**B**) Quantification of relative green fluorescence in worms carrying *frIs7* and *hygR* transgenes (control; IG1864) or also expressing DcEntA (IG1942) or DcEntC (IG1883) either not infected (NI) or 20 h after infection (I) as young adults with *D*. *coniospora* at 25°C. **** p < 0.0001; ns, not significant, one way ANOVA test, n > 80. (**C**) Quantitative RT-PCR analysis of the expression of *nlp* and *cnc* genes in worms carrying *frIs7* and *hygR* transgenes (control; IG1864) or also expressing DcEntA (IG1942) after 18h of infection by *D*. *coniospora*. Results from 2 independent experiments are shown relative to the expression levels in age-matched uninfected worms. (**D-H**) DcEntA acts parallel to or downstream of *gpa-12* and *pmk-1* to block AMP gene expression. (**D**) *D*. *coniospora* infection activates a signal transduction pathway that, via the Gα protein GPA-12, the p38 MAPK PMK-1 and the STAT-transcription factor-like protein STA-2, positively regulates the expression of AMP genes of the *nlp* family. The expression of *cnc* family AMP genes is also induced, but via a distinct *pmk-1*-independent pathway [40] that converges on *sta-2* [48]. In the absence of infection, expression of a constitutively active Gα protein (GPA-12*) or uncleavable p38 MAPK (PMK-1*) leads to higher expression of AMP genes of both the *nlp* and *cnc* families. (**E**, **G**) Representative images of young adult worms carrying *frIs7*, expressing GPA-12* (**E**) or PMK-1* (**G**) and expressing (right, IG1948 and IG1963) or not (left, IG1389 and BPW24) DcEntA. Red and green florescence is visualized simultaneously. Scale bar, 200 μm. (**F**, **H**) Quantitative RT-PCR analysis of the expression of *nlp* and *cnc* genes in worms expressing a constitutively active Gα protein (GPA-12* (IG1389), **F**) or uncleavable p38 MAPK (PMK-1* (BPW24), **H**) and worms also expressing DcEntA (DcEntA;GPA-12* (IG1948), **F**; DcEntA;PMK-1* (IG1963), **H**). Results from 2 independent experiments are shown relative to the expression levels in age-matched IG1864 worms.

One of the key elements in the innate immune response of *C*. *elegans* to *D*. *coniospora* is the increased expression of a battery of AMP genes, including the well-studied *nlp-29* [6,19,39]. When we assayed the effect of DcEntA expression on the induction of an *nlp-29p*::*GFP* reporter that is normally provoked by *D*. *coniospora* infection, we observed an almost complete block, that was not seen in worms expressing DcEntC. Notably, the level of the dsRed reporter, expressed in hyp7 under the constitutive *col-12* promoter, was unchanged (Fig 3A and 3B). Using qRT-PCR, we confirmed the inhibitory effect of DcEntA on *nlp-29* expression after infection. *nlp-29* is one of a cluster of related AMP genes of the *nlp* family that are all up-regulated upon *D*. *coniospora* infection [19]. The expression of a second family of AMP genes, the caenacins (*cnc*), is also induced by the fungus [6,40]. By qRT-PCR, we observed that DcEntA blocked the expression of several AMP genes of both the *nlp* and *cnc* families (Fig 3C). Thus, DcEntA blocks AMP gene expression at the transcriptional level.

### DcEntA acts in parallel to, or downstream of, a canonical p38 MAPK pathway

The main pathway regulating *nlp-29* expression upon infection has been delineated [41–43]. It starts with activation of the G-protein coupled receptor DCAR-1 by the endogenous ligand HPLA [44] and signal transduction via the Gα protein GPA-12 [45]. Expression of a constitutively active form of the latter (referred to as GPA-12*) recapitulates many of the transcriptional changes that accompany infection [5], including an increased expression of *nlp-29* (Fig 3D), and also leads to a high level of expression of the *nlp-29p*::*GFP* reporter, making this a useful tool for epistasis analysis [46].

When we crossed the *DcEntA* transgene into a strain expressing GPA-12*, we observed an abrogation of the high *nlp-29p*::*GFP* reporter expression, and of the elevated expression of endogenous AMP genes as judged by qRT-PCR (Fig 3E and 3F). It should be noted that expression of GPA-12* down-regulates *col-19* expression [5]. Thus, expression of the *col-19p*::*DcEntA* transgene used here is decreased in a gpa-12* background (S5A and S5B Fig), and these worms showed a suppression of the increased susceptibility to *D*. *coniospora* infection normally associated with expression of DcEntA (S5C Fig). Notwithstanding this effect, together these results indicate that DcEntA acts in parallel to, or downstream of, *gpa-12* to block defence gene expression.

The *gpa-12* pathway feeds into a conserved p38 MAPK cascade that ends with *pmk-1* [18]. The activity of p38 MAPK PMK-1 is normally limited by proteolysis by the caspase CED-3. Mutating the caspase cleavage site (changing Asp327 to Glu) in PMK-1 results in higher p38 MAPK activity and increased expression of *nlp-29p*::*GFP* [47]. When we crossed the *DcEntA* transgene into a strain carrying the *pmk-1* (*D327E*) gain-of-function allele, we also observed a block of the elevated expression of the *nlp-29p*::*GFP* reporter and of endogenous AMP genes, although the effect was less dramatic than for the strain expressing GPA-12* since, as previously reported [47], constitutive levels of *nlp* and *cnc* gene expression were only moderately elevated in the *pmk-1*(*D327E*) background (Fig 3G and 3H). This indicates that DcEntA acts in parallel to, or downstream of, *pmk-1* to block *nlp* and *cnc* AMP gene expression.

### DcEntA affects the key immune regulators SNF-12 and STA-2

**Fig 4:**
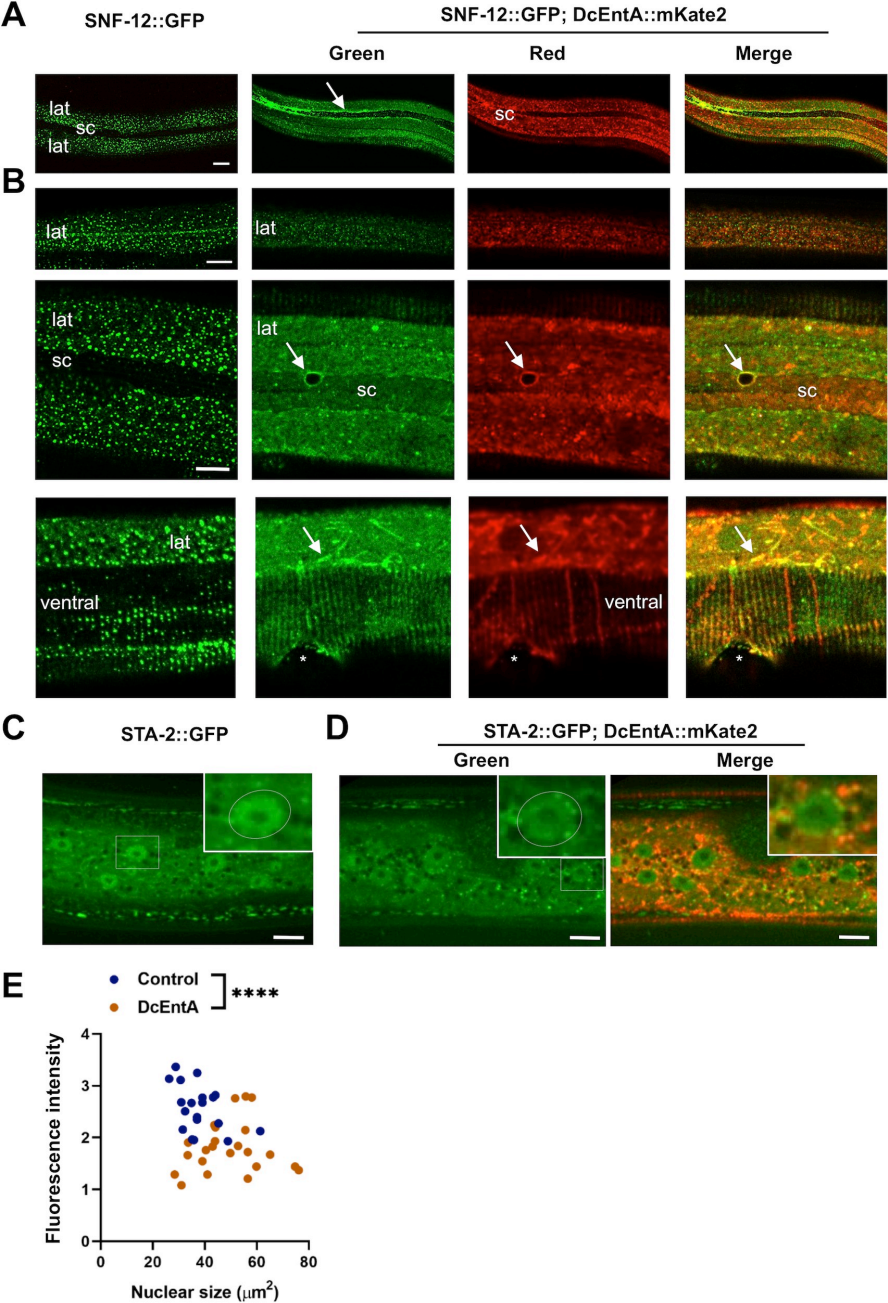
DcEntA alters the localisation of key immune regulatory proteins. (**A**, **B**) DcEntA disrupts the vesicular pattern of SNF-12 in the epidermis. Confocal images of adult worms expressing SNF-12::GFP together with DcEntA::mKate2 (IG1998, right-hand panels, in order, green, red, and green and red channels together) and their siblings without the DcEntA transgene (left panel, green channel only). (**A**) The normal vesicular expression of SNF-12 in the lateral epidermis (lat) is disrupted in young adult worms expressing DcEntA and an accumulation of green fluorescence (arrow) is observed at the junction with the seam cell (sc). (**B**) When DcEntA expression is low in very young adults (upper panels), some of the SNF-12 vesicular pattern is retained. In older worms, when DcEntA is more highly expressed (lower panels), the SNF-12 pattern is diffuse in the lateral epidermis (lat) and underneath the muscle (ventral) and some aggregates can be observed to colocalise with DcEntA (arrow). Scale bar 10 μm, (*, vulva), n > 10. (**C-E**) DcEntA decreases STA-2 nuclear accumulation. Confocal images of young adult worms expressing STA-2::GFP with DcEntA::mKate2 (IG1971, **D**; left panel green channel only, right panel green and red channels shown together) and their siblings without the DcEntA transgene (**C**). STA-2::GFP levels are reduced in the epidermal nucleus (white box, enlarged insert; highlighted by white oval) in the presence of DcEntA. Scale bar, 20 μm. (**E**) Intensity of green fluorescence in the nuclei of DcEntA;STA-2::GFP worms (DcEntA, IG1971, brown) and their siblings without the DcEntA transgene (Control, blue), plotted against nuclear size, measured using Fiji. Each dot represents a nucleus; n > 20. The difference between the 2 populations is significantly different (**** p < 0.0001; unpaired t-test).

The p38 MAPK PMK-1 acts upstream of STA-2, a STAT-like protein. STA-2 is the common transcriptional regulator of *nlp* and *cnc* AMP genes. It interacts physically and functionally with the SLC6 protein SNF-12 [48]. When we crossed the *DcEntA* transgene into a strain expressing a SNF-12::GFP reporter protein, we observed a disruption of its normal vesicular pattern at the apical surface of the hyp7 epidermal syncytium (Fig 4A). In young adult transgenic worms with low DcEntA expression, the vesicular fluorescence was decreased (Fig 4B, upper panel, n>10). A few hours later into adulthood, when DcEntA expression was higher, the SNF-12::GFP signal was largely diffuse within the cytoplasm, with some colocalisation of green and red fluorescence and accumulation at the boundary with the seam cells, as well as in a filamentous pattern at the apical surface of hyp7 (Fig 4B, middle and lower panels, n>10 for each). Since SNF-12’s correct intracellular localisation is essential for the induction of AMP gene expression [49], this disruption could be the cause of DcEntA’s inhibitory effect.

STA-2 is found in both the cytoplasm and nuclei of the hyp7 epidermal syncytium of uninfected worms [48]. Upon *D*. *coniospora* infection, at early time points, it is technically challenging to correlate increasing AMP expression with the translocation of STA-2 into epidermal nuclei in individual worms, due in part to the inhomogeneous adhesion of spores to the worms’ surface. Later, when infection is more homogeneous, increased AMP gene expression is accompanied by a significantly higher level of nuclear STA-2 (S6 Fig). Expression of DcEntA alone, in the absence of infection, significantly reduced the amount of a STA-2::GFP reporter protein within the nucleus (Fig 4C–4E). Thus, since STA-2 has DNA-binding ability (J. Polanowska, personal communication) and is expected to be a direct transcriptional regulator, DcEntA could abrogate *nlp* and *cnc* AMP gene expression by preventing the accumulation of STA-2 in the nucleus upon infection, potentially indirectly, through an effect on membrane trafficking and the activity of SNF-12.

### DcEntA increases the expression of *ifas-1*

**Fig 5:**
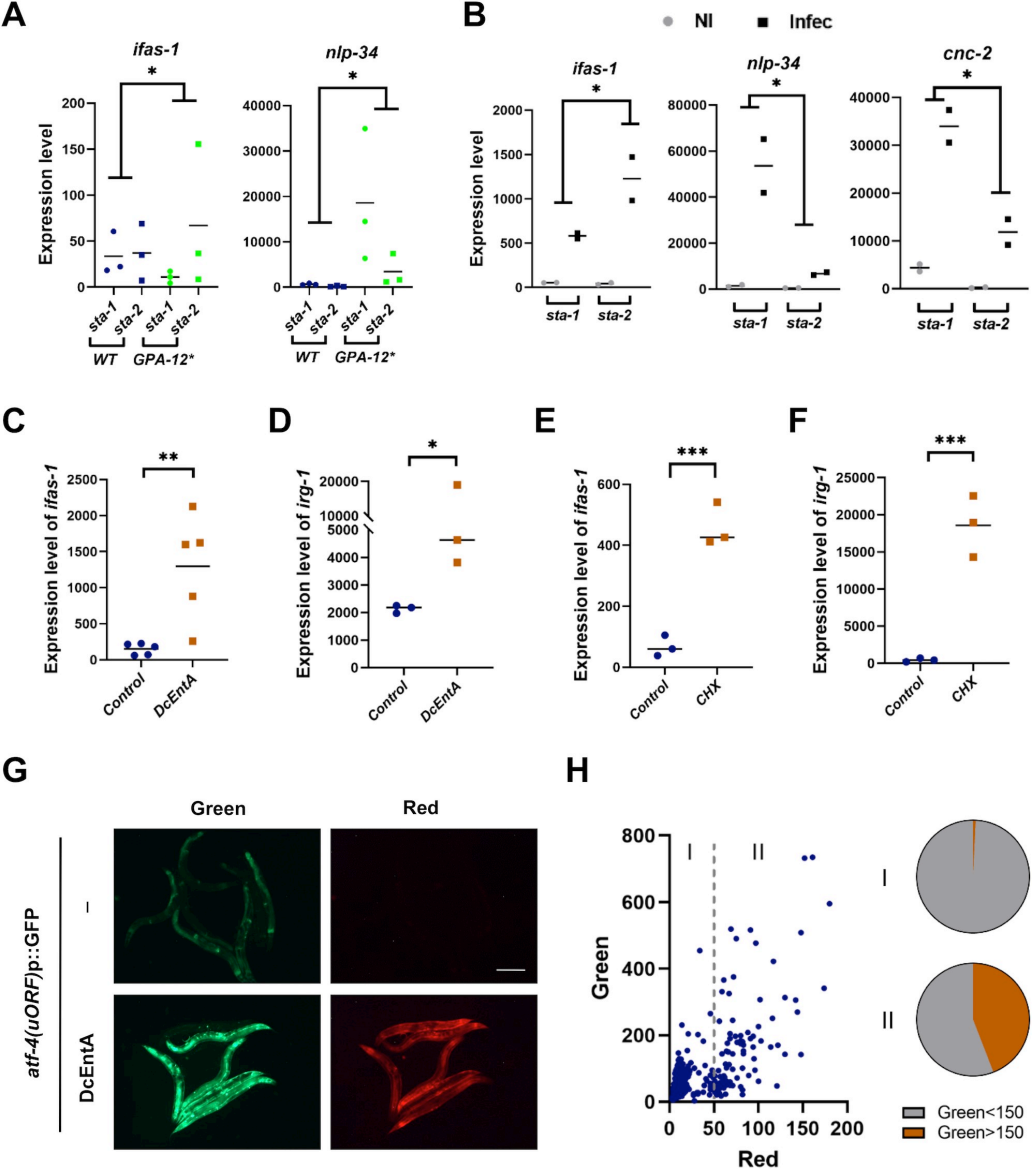
DcEntA induces expression of *ifas-1*, a target of negative regulation by *sta-2*, and inhibits translation. (**A**) Quantitative RT-PCR analysis of the expression of *ifas-1* and *nlp-34* in control (hygR;*frIs7* IG1864) worms and worms expressing GPA-12* (IG1389) following RNAi against *sta-1* or *sta-2*. Data from three independent experiments are shown. (**B**) Quantitative RT-PCR analysis of the expression of *ifas-1*, *nlp-34* and *cnc-2* following RNAi against *sta-1* or *sta-2* in worms infected for 18h (Infec) and non-infected (NI) controls in the epidermis-specific RNAi strain IG1502. Data from two independent experiments are shown. (**C, D**) Quantitative RT-PCR analysis of the expression of *ifas-1* (**C**) and *irg-1* (**D**) genes in young adult worms carrying *hygR* and *frIs7* with (IG1942) or without DcEntA (control; IG1864). (**E, F**) Quantitative RT-PCR analysis of the expression of *ifas-1* (**E**) and *irg-1* (**F**) in control worms (carrying *frIs7* [*nlp-29p*::*GFP*; *col-12p*::*dsRed*]; IG274) or following exposure to cyclohexamide (CHX) for 6 h. (**G**) Representative fluorescence images of adult worms carrying an *atf-4p(uORF)*::GFP (Green) reporter also expressing DcEntA::mKate2 (Red, IG2044; bottom panels) and their siblings without the DcEntA transgene (upper panels). Scale bar, 200 μm. (**H**) Relative green fluorescence plotted against red fluorescence in the mixed progeny of IG2044 worms. Each dot represents a single worm (left panel). As the DcEntA transgene is not integrated, the population contains worms expressing or not DcEntA. They can be distinguished on the basis of the red fluorescence associated with DcEntA. Right panel: the proportion of the worms with a high (> 150 a.u.) green fluorescence among those with a low (<50, population I, n = 892) or high (>50, population II, n = 102) level of red fluorescence. In population I, that corresponds to worms that do not express DcEntA, only 0.78% have Green >150, while in population II, 44.12% do. (**A-F**) paired one-sided t test, * p < 0.05; ** p < 0.01; *** p < 0.001. (**A**) and (**B**) the fold-change in expression level between the 2 indicated conditions is significantly different.

Both *nlp* and *cnc* AMP genes are positively regulated by the STAT-like transcription factor STA-2 [48]. The gene *ifas-1* (“inducible fascin domain-containing”; *F40H7*.*12*), on the other hand, is induced upon *D*. *coniospora* infection [50] but this does not require *sta-2* [51]. When we knocked down *sta-2* expression by RNAi in worms expressing GPA-12*, we observed the expected decrease in the expression of the AMP gene *nlp-34*, but a significant increase in the expression of *ifas-1* (Fig 5A). Similarly, upon infection, knocking down *sta-2* specifically in epidermis (in strain IG1502 [52]) decreased expression of the AMP genes *nlp-34* and *cnc-2*, but provoked a significant increase in the expression of *ifas-1* (Fig 5B). In vertebrates, as well as acting as positive regulators of gene expression, STAT proteins can directly repress the accessibility and transcription of specific loci [53]. Indeed, the only other STAT protein in *C*. *elegans*, STA-1, is known to be a repressor of virus infection response genes [54]. Our results suggest that in addition to acting as a positive regulator of AMP gene expression, STA-2 acts in a cell-autonomous manner to repress *ifas-1* expression. Consistent with this model, the expression of *ifas-1* was markedly increased by DcEntA and this effect was abrogated when STA-2 was activated in the GPA-12* background (Figs 5C and S5D). Thus, we hypothesise that DcEntA exerts opposite effects on the expression of *ifas-1* and of the *nlp* and *cnc* AMP genes by decreasing the level of STA-2 in the nucleus.

### *D*. *coniospora* infection and DcEntA inhibit translation

*ifas-1* shares some of the characteristics of “Intracellular Pathogen Response” genes [55], including regulation by *pals-22* and *pals-25* [56], and the fact that like *irg-1* and *irg-2* [57], its expression is strongly induced by the translational elongation inhibitor cycloheximide (CHX; Fig 5E and 5F). Thus, upon *D*. *coniospora* infection, or heterologous expression of DcEntA, the level of *ifas-1* transcription is potentially increased as a consequence of translation inhibition.

The capacity to detect perturbation of normal protein synthesis is an important part of the response of *C*. *elegans* to *Pseudomonas aeruginosa* infection [57,58]. In this case, it involves the bZIP transcription factor ZIP-2 [59] that, on the basis of publicly available ModERN ChiP-seq data [60], directly regulates *irg-1* and *irg-2*. On the other hand, neither *irg-1* nor *irg-2* is induced after 12 or 24 hours of infection by *D*. *coniospora* [50], and in line with the ModERN ChiP-seq data, *zip-2* has not been reported to regulate *ifas-1* expression [59]. As described above, the expression of *ifas-1*, as well as of *irg-1*, was markedly increased by DcEntA (Fig 5C and 5D), consistent with DcEntA interfering with translation.

To extend these observations, we examined the effect of infection on an ATF4/ATF-4 reporter gene. This contains two upstream open reading frames (uORF) before the GFP coding sequence [61]. Under normal conditions, the uORFs are translated, to the detriment of GFP expression. When translation rates are reduced, ribosomal scanning can bypass the uORFs, and translation re-initiation occur at the downstream initiation site, leading to translation of GFP [62]. Infection of adult worms carrying the *atf-4*p(uORF)::GFP reporter by *D*. *coniospora* was associated with a marked increase in GFP expression (S7 Fig). A strain carrying the *atf-4*p(uORF)::GFP reporter and also expressing DcEntA exhibited high GFP in the adult epidermis in the absence of infection (Fig 5G and 5H). Together, these results are consistent with *D*. *coniospora* infection, and expression of DcEntA alone, causing a reduction in translation in the epidermis, a hitherto uncharacterised effect.

Expression of ATF-4 can be increased in response to endoplasmic reticulum (ER) stress through PERK1/PEK-1 phosphorylation of the initiation factor eIF2α, which reduces global translation rates [63]. PERK1/PEK-1 activation is also a hallmark of the ER unfolded protein response (UPR^ER^) [64]. Infection of adult worms does not induce the UPR^ER^, or the expression of UPR^ER^ genes such as *hsp-4* [65]. Expression of DcEntA in the adult epidermis did not increase *hsp-4* expression either. Further, the expression of *hsp-6* and *hsp-60* that are markers of the mitochondrial UPR, as well as *gst-4* (oxidative stress) and *gpdh-1* (osmotic stress) [66–70], showed no increase of expression in worms expressing DcEntA (S8 Fig). Together these results indicate that DcEntA exerts a specific inhibitory effect on the expression of *nlp* and *cnc* AMP genes that are positively regulated by *snf-12* and *sta-2*. This is not the consequence of a generalized suppression of gene expression, since DcEntA increases expression of the infection-regulated gene, *ifas-1*, potentially as it interferes with translation, and in part as a consequence of reducing a suppressive effect of *sta-2*.

### DcEntA likely has ADP-ribosylation activity

**Fig 6:**
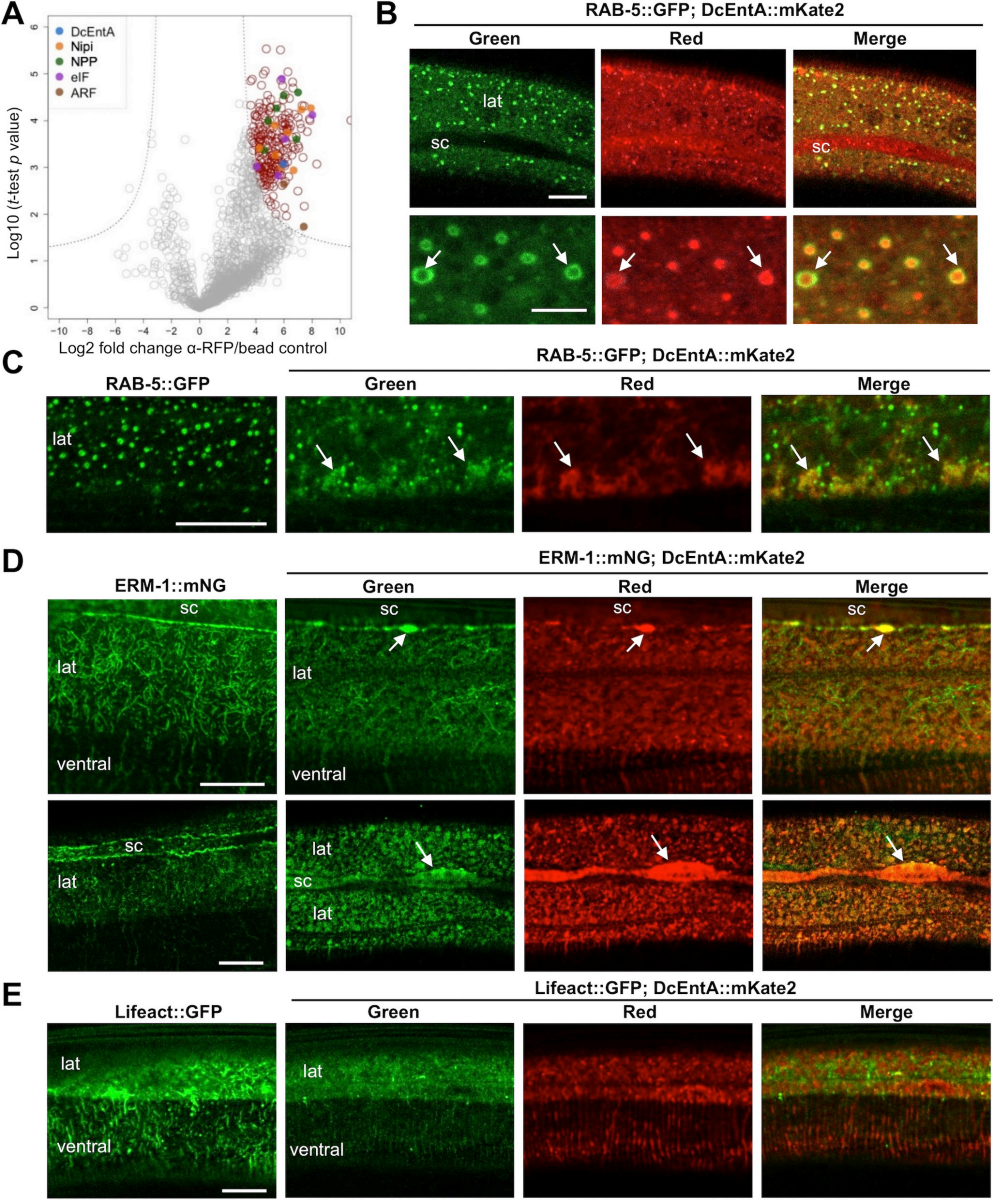
DcEntA interacts with many candidate host proteins and disrupts endocytosis and the host cytoskeleton. (**A**) The relative abundance of proteins co-precipitated with DcEntA::FLAG::Degron::mKate2 was assessed by mass spectrometry. Volcano plot showing specific interaction partners (in red) of DcEntA::FLAG::Degron::mKate2 (DcEntA in blue). The mean values for fold change from 3 independent experiments are shown. The SAM (significance analysis of microarrays) algorithm was used to evaluate the enrichment of the detected proteins. Proteins that met the combined enrichment threshold (hyperbolic curves, *t*_*0*_ = 2) are coloured in red. The 2 *C*. *elegans* ARF proteins are shown in brown, known members of the nuclear pore complex (NPP) are in green, eukaryotic initiation factor proteins (eIF) in purple and proteins corresponding to Nipi (No Induction of Peptide after *Drechmeria* Infection) genes in orange. (**B**) Confocal images of young adult worms expressing RAB-5::GFP together with DcEntA::mKate2 (IG2022, panels, in order, green, red, and green and red channels together) in the epidermis. Selected instances of DcEntA’s colocalisation with RAB-5 are highlighted with arrows. Scale bar 10 μm in the upper panel, 5 μm in the lower panel. (**C**) Confocal images of young adult worms expressing RAB-5::GFP together with DcEntA::mKate2 (IG2022, right-hand panels, in order, green, red, and green and red channels together) in the epidermis, and their siblings without the DcEntA transgene (left panel). When DcEntA expression is higher, some RAB-5 signal becomes diffuse in the cytoplasm of the lateral epidermis (lat), colocalising with DcEntA (arrow). (**D**) Confocal images of young adult worms expressing ERM-1::mNG together with DcEntA::mKate2 (IG2051, right-hand panels, in order, green, red, and both channels together) in the epidermis, and their siblings without the DcEntA transgene (left panels). When DcEntA expression is low (upper panels), some ERM-1 is still observed in a fibre pattern, as well as with a localisation at the junction with the seam cell, co-localised with DcEntA (arrow). When DcEntA expression is higher, (lower panels), the ERM-1 pattern becomes diffuse at the seam cell (sc) boundary and some aggregates can be observed in the cytoplasm, co-localised with DcEntA. Scale bar 10 μm, n> 10. (**E**) DcEntA disrupts the actin cytoskeleton. Confocal images of young adult worms expressing Lifeact::GFP together with DcEntA::mKate2 (IG2024, right-hand panels, in order, green, red, and green and red channels together) in the epidermis, and their siblings without the DcEntA transgene (left panel). Scale bar 10 μm.

As a first step to understand the basis of these pleiotropic effects, we took an unbiased biochemical approach to identify proteins that interacted with DcEntA *in vivo*. From a synchronized population of adult worms expressing the DcEntA fusion protein, we pulled down DcEntA::FLAG::Degron::mKate2 by immunoprecipitation from whole worm extracts and subjected the purified proteins to mass spectrometry analysis. Samples from four other strains of transgenic worms, each expressing a different candidate virulence factor, were processed in parallel (see below; Harding *et al*., in preparation). These were used as control samples, allowing proteins that specifically interacted with DcEntA to be identified. As expected, DcEntA itself featured among the most abundant proteins identified (Fig 6A and S4 Table). Detailed examination of its spectrum supported the presence of at least one site with an ADP-ribosylation modification, at an asparagine in the non-conserved C-terminus of the protein (S9 Fig). This suggests that in common with some bacterial exotoxins [7], DcEntA is able to auto-ADP-ribosylate.

Cholera toxin from *Vibrio cholera* requires a host protein, ADP ribosylation factor (ARF), in order to ADP-ribosylate its targets [71,72]. Notably, the 2 nematode orthologues of human ARF1 and ARF3, ARF-1.2 and ARF-1.1, respectively, were found among the 245 candidates interactors of DcEntA, consistent with a conserved mode of action for this fungal heat-labile enterotoxin. Attempts to confirm the functional relevance of this interaction were hampered by the essential role of ARF proteins; *arf-1*.*2(RNAi)* alone, or in combination with *arf-1*.*1(RNAi)* rendered worms sick, and we did not pursue the question further.

### DcEntA potentially affects diverse aspects of host cell physiology

To get an overall view of the proteins that were identified by mass spectrometry as candidate interactors of DcEntA, we used the gene set enrichment analysis tools available within Wormbase [73]. Among the enriched phenotype classes, given the disruptive effect of DcEntA on the vesicular pattern of SNF-12, “vesicle organization variant” (WBPhenotype:0001671; p = 5.4x10^-3^) stood out. With regards gene ontology, the most highly over-represented term was the “Cellular Component” class “ribonucleoprotein granule” (GO:0035770; p = 5.6 x10^-6^). This class is for components of a “non-membranous macromolecular complex containing proteins and translationally silenced mRNAs”. This is consistent with the observed increase in *irg-1* expression, a marker of translation inhibition [57,58], provoked by DcEntA, and the enrichment for “peptide biosynthetic process” (GO:0043043; p = 1.2x10^-4^) and several other translation-related classes (S5 Table). DcEntA also disrupts membrane trafficking and we found an enrichment in the class “cellular macromolecule localization” (GO:0070727; p = 1x10^-4^). Indeed, several components of *C*. *elegans* intracellular vesicle transport machinery were identified, including 16 that have been reported to interact physically with VPS-45, orthologue of human VPS45 (vacuolar protein sorting 45 homolog), required for RAB-5-dependent endocytic transport [74], and 11 interactors of LET-413 [75], the nematode Erbin protein that acts as a RAB-5 effector during endocytic recycling and that physically interacts with RAB-5 [76]. Consistent with these observations, DcEntA was found in RAB-5-associated vesicles (Fig 6B). Notably, similar to the observed effect of DcEntA on the pattern of SNF-12::GFP (Fig 4B), the normal pattern of RAB-5 expression was disrupted in worms expressing high levels of DcEntA, with fewer RAB-5-positive vesicles and a more diffuse pattern of fluorescence (Fig 6C, n > 10 worms).

The list of candidate DcEntA interactors also included several proteins involved in cytoskeleton dynamics, including the nematode ezrin/radixin/moesin (ERM) orthologue ERM-1, the microtubule plus-end binding protein EBP-1, and the twinfilin homologue TWF-2, an actin binding protein. One of the main functions of ERM proteins is to link the plasma membrane and the actin cytoskeleton. We observed ERM-1 in randomly orientated fibres in the lateral epidermis, consistent with its reported localisation in other epithelial tissues in *C*. *elegans* [77], and increased at the baso-lateral junction with the seam cell (Fig 6D, left panel). When DcEntA expression was low, we observed a partial colocalisation with ERM-1 at the junction with the seam cell. When DcEntA expression was high, the normal pattern of ERM-1 in fibres was disrupted, the two proteins co-localised in aggregates in the cytoplasm. Additionally, the normal specific localisation of ERM-1 in hyp7 at the baso-lateral junction with the seam cell was replaced by a diffuse expression over the entire surface of contact between the 2 cells (Fig 6D, upper and lower panels respectively, n>10 worms). DcEntA expression also disrupted the actin cytoskeleton, including the prominent cortex underneath the muscle (Fig 6E). Coordinated changes in microtubule and actin dynamics are required for the proper recruitment of SNF-12 to sites of injury in hyp7 [49]. There are thus several potential ways that DcEntA could influence the localisation SNF-12.

There were also 6 NPP proteins, components of the nuclear pore, required for nucleocytoplasmic transport, and at least 5 involved in translation initiation. Two of each category (NPP-1 and NPP-6; EIF-2gamma and F33D11.10, respectively) had previously been implicated in the regulation of AMP gene expression: the 4 corresponding genes were identified in a whole-genome RNAi screen for positive regulators of *nlp-29* expression, and are thus Nipi (No Induction of Peptide after *Drechmeria* Infection) genes [43]. Another 8 candidate DcEntA interactors correspond to Nipi genes (Fig 6A and S4 Table). Thus, it is possible that DcEntA has multiple modes of action: affecting translation, altering endocytosis and the cytoskeleton, as well as blocking nuclear import of STA-2 via an effect on SNF-12 and/or alteration of nuclear pores (see model below).

### DcEntB affects nucleolar size and shape

**Fig 7:**
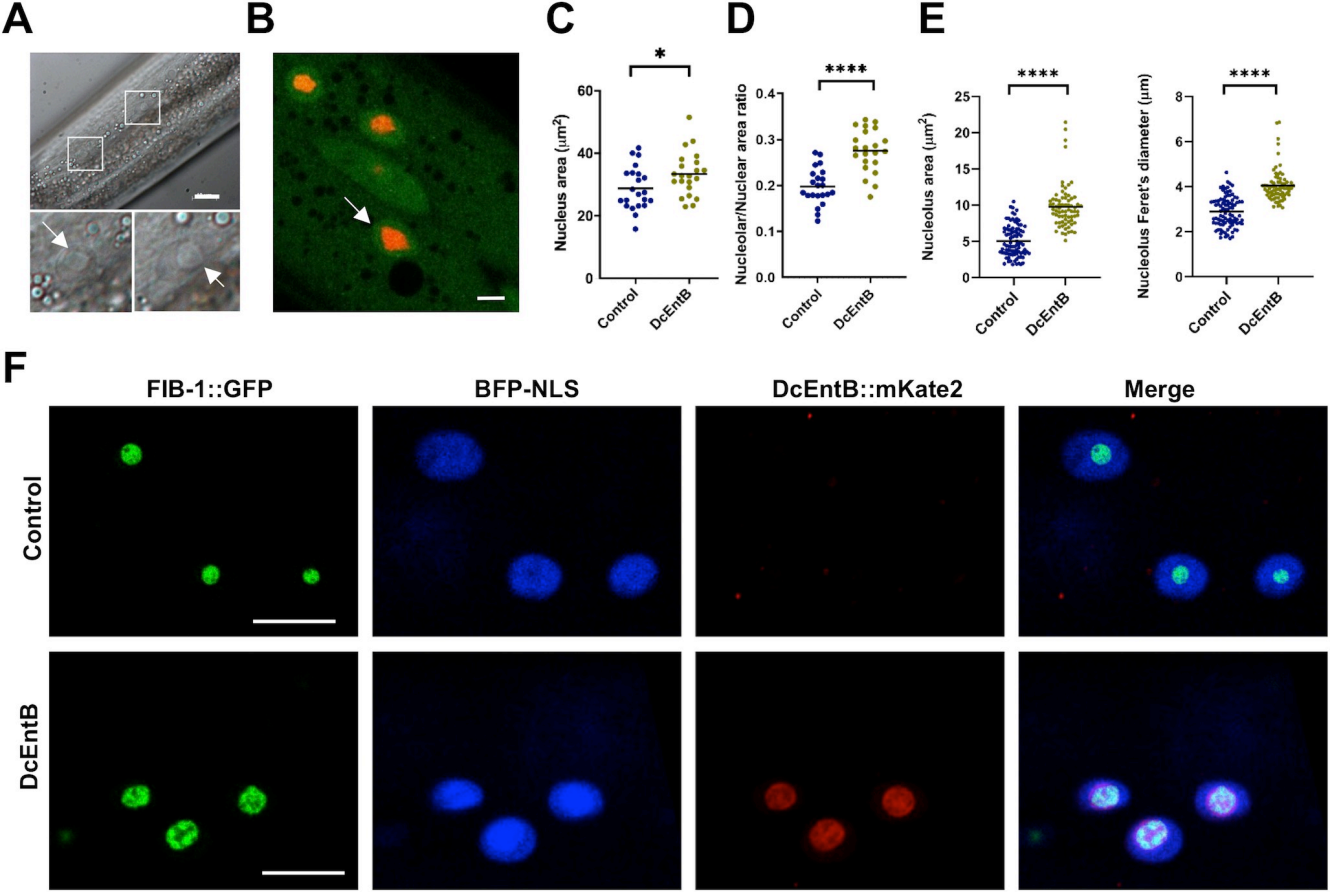
DcEntB makes nucleoli irregular and larger. (**A**) Representative Nomarski image of a young adult worm expressing DcEntB::mKate2. Scale bar, 10 μm. Enlarged views of the boxed regions show the large and irregular nucleoli (white arrows). (**B**) Representative confocal image of a young adult worm expressing DcEntB::mKate2 and STA-2::GFP (IG1977) in hyp7. White arrow points to an irregular nucleolus (red). Scale bar, 10 μm. (**C**, **D**) Quantification of nuclear size (**C**) and nucleolus/nucleus ratio (**D**) in the epidermis of young adult IG1977 worms (DcEntB) and their siblings without the DcEntB transgene (Control). n > 20 in each strain; bars represent the mean; * p < 0.05, unpaired t-test **** p < 0.0001, unpaired t-test. (**E**) Quantification of nucleolus area and Feret’s diameter in hyp7 of young adult worms expressing FIB-1::GFP together with (DcEntB; IG1984) or without (Control; IG1596) DcEntB. **** p < 0.0001. Statistical significance was determined using a nonparametric Mann Whitney test. (**F**) Confocal images of hyp7 nuclei in young adult worms expressing FIB-1::GFP together with (DcEntB; IG1984; lower panels) or without (Control; IG1596; upper panels) DcEntB. All worms also express BFP-NLS; panels from left to right: green, blue, red, and the 3 channels together, scale bar, 5 μm.

Turning to DcEntB, as described above, it localises to the nucleolus. Nucleoli are the site of ribosome biogenesis and also play a role in cells’ responses to diverse stresses [78]. They have been linked to the regulation of immune responses against bacterial pathogens in *C*. *elegans* [79,80]. Interestingly, we observed a clear alteration of nucleolar morphology in worms expressing DcEntB, with many nucleoli exhibiting strikingly angular shapes rather than their usual spherical form (Fig 7A and 7B). This is reminiscent of the morphological changes that accompany several different pharmacological treatments, including ATP depletion, in mammalian cells [81]. Expression of DcEntB was associated with an increase in nuclear size (Fig 7C), and a proportionately larger increase in the size of nucleoli (Fig 7D). We also used the FIB-1::GFP reporter [37] to characterise and quantify these changes, and confirmed a highly significant increase in nucleolus size and Feret’s diameter. The latter is a measure of the longest distance between any two points along the nucleolar boundary and when compared to area gives an indication of any deviation from circularity (Figs 7E and 7F and S10A). This suggests that DcEntB will have a direct effect on nucleolus biology.

### DcEntB increases AMP gene expression and can prime the immune system

**Fig 8:**
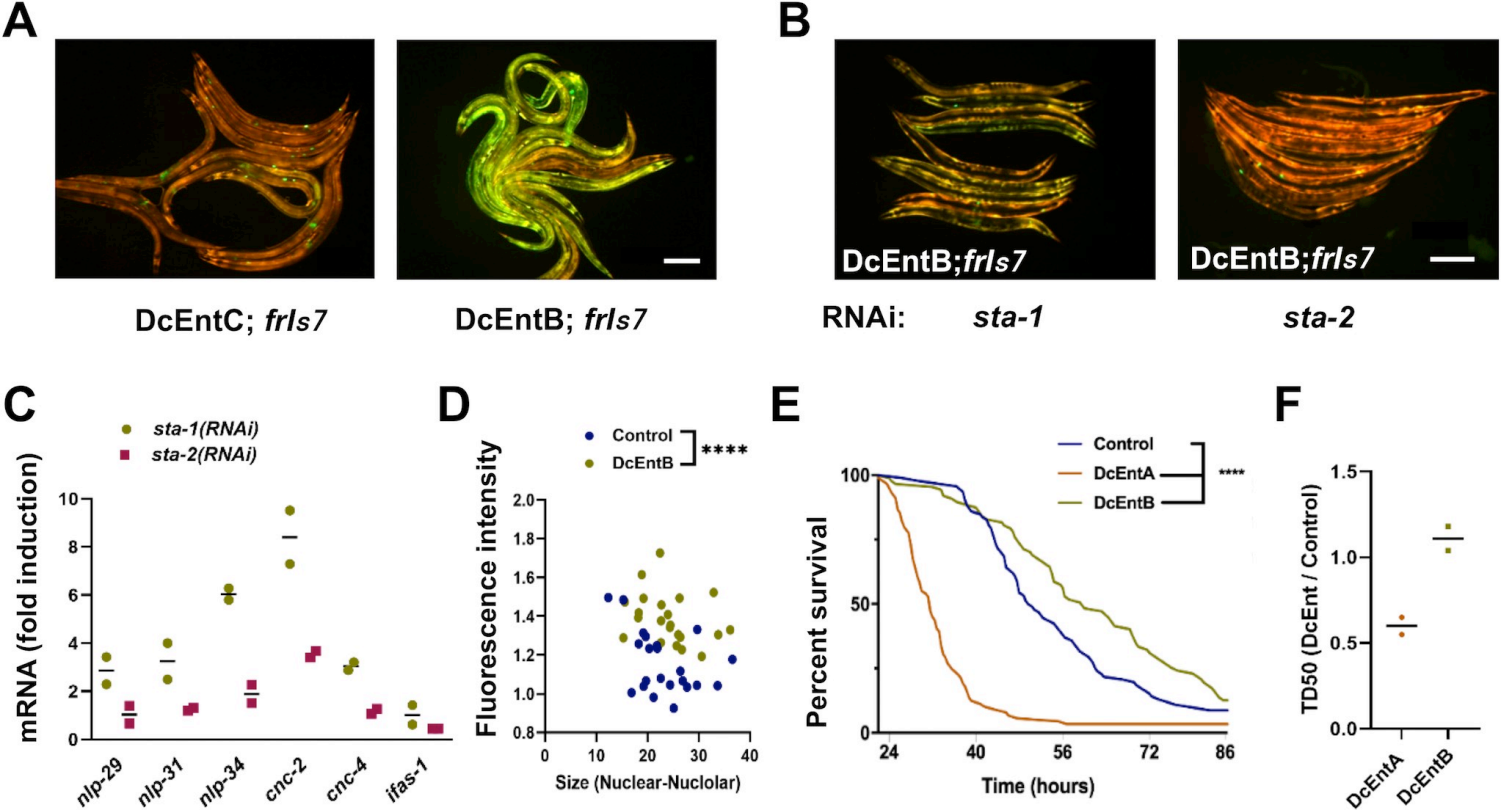
DcEntB induces AMP gene expression in a *sta-2*-dependent manner. (**A**, **B**) Representative images of adult worms, 3 days after the L4 stage, carrying *frIs7* and expressing DcEntC (IG1883) or DcEntB (IG1941) on *E*. *coli* OP50 (**A**) or following RNAi against *sta-1* or *sta-2* (**B**). *frIs7* includes *nlp-29p*::*GFP* and *col-12p*::*dsRed* transgenes; red and green florescence is visualized simultaneously. Scale bar, 200 μm. A difference in GFP levels for worms with *frIs7* between OP50 and RNAi (HT115) bacteria has been observed regardless of the genetic background. (**C**) Quantitative RT-PCR analysis of the expression of *nlp*, *cnc* and *ifas-1* genes in worms expressing DcEntB (IG1941) following RNAi against *sta-1* or *sta-2*. Results from 2 independent experiments are shown relative to the expression levels in age-matched control (hygR;*frIs7* IG1864) worms. (**D**) Intensity of green fluorescence in the nuclei of DcEntB;STA-2::GFP worms (DcEntB, IG1977, green), and their siblings without the DcEntB transgene (Control, blue), plotted against the nuclear-nucleolar size, measured using ImageJ. Each dot represents a nucleus; n>20, **** p < 0.0001, unpaired t-test. (**E**) Survival of control (IG1864) worms and worms expressing DcEntA (IG1942) or DcEntB (IG1941) after infection as young adults with a concentration of *D*. *coniospora* spores 10 times higher than usual at 25°C (n = 92, 91 and 87 respectively). **** p < 0.0001, one-sided log rank test. The curves here are representative of 2 independent biological replicates for which the ratio of median survival (TD50) between worms expressing DcEntA or DcEntB to control worms are shown in (**F**).

In direct contrast to the effect of DcEntA, expression of DcEntB was associated with an increase in the constitutive expression of the *nlp-29p*::*GFP* reporter (Fig 8A). This increase was dependent upon STA-2 since *sta-2*(*RNAi*) reduced reporter gene expression back to the normal level (Fig 8B). Using qRT-PCR, we confirmed the positive effect of DcEntB on *nlp-29* expression, and demonstrated a similar *sta-2*-dependent effect for several *nlp* and *cnc* genes (Fig 8C). Notably, worms expressing DcEntB did not exhibit any change in *ifas-1* expression (Fig 8C). Together, these results suggest that DcEntB might act through the canonical STA-2 pathway to regulate AMP gene expression. Supporting this, when we crossed the DcEntB transgene into a strain expressing STA-2::GFP, we observed a significant increase in the amount of STA-2::GFP in the nucleus (Fig 8D).

There is therefore a striking dichotomy between the effects of DcEntA and DcEntB. DcEntA appears to act as many known virulence factors do, blocking the activation of an immune defence pathway, in this case potentially via an inhibition of the activity of the transcription factor STA-2. DcEntB, on the other hand, appears to activate the same pathway, leading to more STA-2 in the nucleus and more AMP gene expression. We hypothesised that this might reflect a host defence strategy wherein the presence of DcEntB is detected, either directly or indirectly, as a form of surveillance immunity. Should this be the case, one would predict that expression of DcEntB might increase survival following infection. Although we showed that following infection under standard conditions DcEntB-expressing worms had an increased susceptibility to infection (Fig 2D), when we infected the same strain of worms with *D*. *coniospora* using a very high concentration of spores, in contrast to worms expressing DcEntA that died more rapidly, the DcEntB-expressing worms were indeed significantly resistant, and lived longer even than the controls (Fig 8E and 8F). Thus, presumably as a consequence of its complex effect on gene expression and the dynamics of the infection process, depending on the infectious burden, DcEntB can have positive or negative effects on resistance to infection. Nevertheless, the presence of high levels of DcEntB does appear to have the capacity to prime the host immune system.

### DcEntB-induced changes in nucleolar morphology require STA-2 and are associated with a specific induction of targets of the p38 MAPK pathway

**Fig 9:**
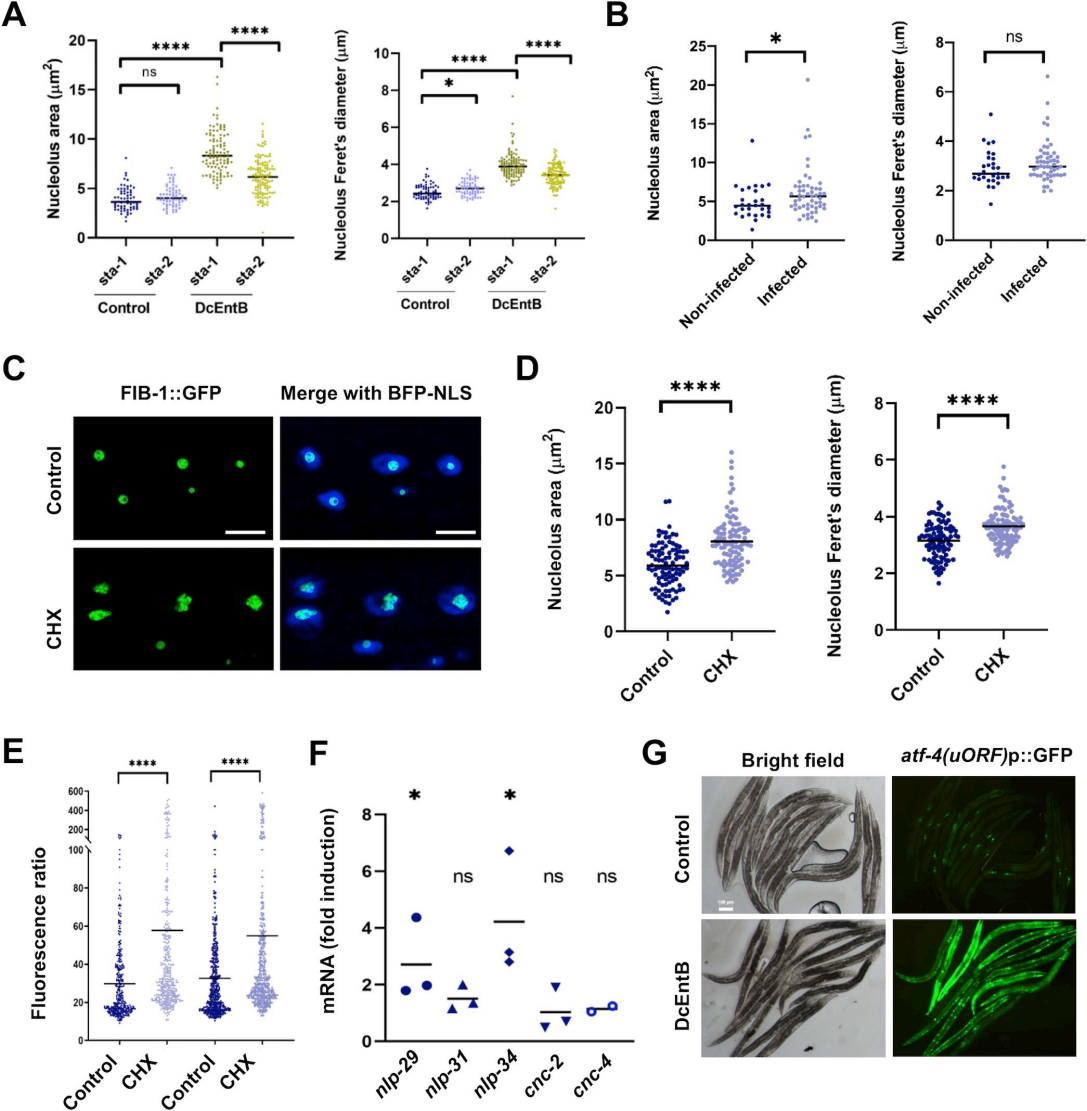
Relationship between nucleolar morphology, translation and AMP gene expression. (**A**, **B**) Quantification of nucleolus area and Feret’s diameter in young adult worms expressing FIB-1::GFP together with (DcEntB; IG1984) or without (Control; IG1596) DcEntB following RNAi against *sta-1* or *sta-2* (**A**) or in IG1596 worms following 24 h infection (**B**). (**C**) Representative confocal images of young adult IG1596 worms (that express BFP-NLS in addition to FIB-1::GFP) after exposure for 6 h to cycloheximide (CHX; lower panels) compared to control (upper panels); left and right panels: green, and green and blue channels together, respectively), scale bar, 5 μm. (**D**) Quantification of nucleolus area and Feret’s diameter in young adult worms expressing FIB-1::GFP (IG1596) after exposure for 6h to CHX, compared to control untreated worms. (**E**) Quantification of relative green fluorescence of young adult worms carrying *frIs7* ([*nlp-29p*::*GFP*; *col-12p*::*dsRed*]; IG274) after exposure for 6 h to CHX, compared to control untreated worms. The results from 2 independent experiments are shown. (**F**) Quantitative RT-PCR analysis of the expression of *nlp* and *cnc* genes in worms carrying *frIs7* (IG274) following exposure as young adults to CHX for 6 h. Results from 3 independent experiments are shown as averages with standard deviation, relative to the expression levels in age-matched control worms. Statistical significance was determined using a nonparametric Mann Whitney test; * p < 0.05, **** p < 0.0001, ns, not significant. (**G**) Representative images (left panels: white light, right panels: green fluorescence) of one day old adult worms carrying an *atf-4p(uORF)*::GFP reporter also expressing DcEntB (IG2045; bottom panels), and their siblings without the DcEntB transgene (upper panels). Scale bar, 200 μm.

To investigate the link between the observed changes in STA-2 nuclear occupancy, AMP gene expression and nucleolar morphology, we first assayed whether *sta-2*(*RNAi*) affected the shape of nucleoli, using the FIB-1::GFP reporter strain. In contrast to control worms, upon *sta-2* inactivation, we observed a very marked decrease in the DcEntB-associated nucleolar phenotypes, both size and Feret’s diameter (Figs 9A and S10B), suggesting that the change in nucleolar morphology could be a consequence of DcEntB’s recruitment of STA-2 to the nucleus and/or the resulting STA-2-dependent changes in gene expression. Notably, when we quantified nucleolar morphology in the FIB-1::GFP reporter strain infected with *D*. *coniospora*, we observed a modest increase in size relative to uninfected controls, without a significant change in Feret’s diameter (Figs 9B and S11A).

**Fig 10:**
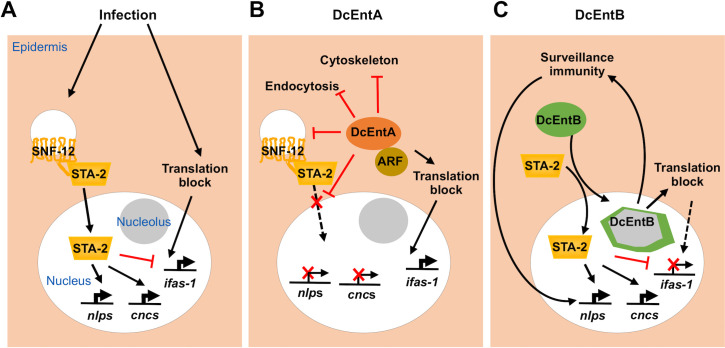
Models of the innate immune response to *D*. *coniospora* infection and the action of DcEntA and of DcEntB. (**A**) Infection by *D*. *coniospora* activates a pathway involving SNF-12 and STA-2. The translocation of STA-2 into the nucleus leads to an increase in the expression of *nlp* and *cnc* genes. An uncharacterized parallel pathway, potentially linked to surveillance of translation, activates *ifas-1*, which is negatively regulated by STA-2. (**B**) DcEntA, via its interaction with host ARF GTPases and other protein partners (brown circle), through ADP ribosylation, potentially interferes with the normal innate immune response at multiple levels. It alters SNF-12 localization, blocks STA-2 nuclear translocation and AMP gene expression. It inhibits translation, leading to an increase in *ifas-1* expression, accentuated by the loss of the repressive function of STA-2. DcEntA interferes with endocytosis, disrupts the cytoskeleton and causes increased cuticle fragility, potentially further increasing susceptibility to infection. (**C**) When DcEntB is expressed in hyp7, it localises to the nucleolus. Expression of DcEntB drives STA-2 into the nucleus, leading to an increase in *nlp* and *cnc* gene expression and suppressing *ifas-1* expression. DcEntB alters the shape and size of the epidermal nucleoli. This provokes a surveillance mechanism leading to the expression of *nlp* but not *cnc* genes.

To explore further the relationship between nucleolar morphology and the *sta-2*-dependent immune response, we treated worms carrying FIB-1::GFP with CHX that, in addition to affecting protein synthesis, is also known to alter nucleolar shape [81]. Exposure of adults to 500 μg/ml CHX for 6 h caused nucleoli to become larger and less round (Figs 9C and 9D and S11B). Additionally, it led to a modest but significant increase in the expression of the *nlp-29p*::*GFP* reporter (Fig 9E). Using qRT-PCR, we confirmed this effect on *nlp-29* expression, and demonstrated a similar effect for *nlp-34* (Fig 9F), albeit to a lesser degree than *irg-1*, which responds strongly to CHX treatment (Fig 5F; [57]). Notably, the expression of *cnc-2* and *cnc-4* was not affected by exposure to CHX (Fig 9F). These 2 genes are not regulated by p38 MAPK PMK-1 upon *D*. *coniospora* infection [40]. When we crossed the DcEntB-expressing strain with a strain carrying the *atf-4*p(uORF)::GFP reporter, we observed a large increase in GFP expression in adult worms (Fig 9G), consistent with an inhibitory effect of DcEntB on protein synthesis. Taken together with the fact that DcEntB does not increase the expression of *ifas-1* (Fig 8C), but CHX treatment does (Fig 5E), these results suggest that the potential inhibition of protein synthesis by DcEntB leads to a specific induction of a p38 MAPK dependent immune response in the epidermis, in addition to its more direct effect on STA-2 activity. The action of DcEntB can be contrasted with the model for the effects of DcEntA that, directly or indirectly, prevents accumulation of STA-2 in the nucleus and thereby inhibits the expression of multiple defence genes, rendering *C*. *elegans* more susceptible to infection. At the same time, potentially as a counter-defensive mechanism, loss of STA-2-dependent repression accentuates the increase in the expression of *ifas-1* provoked by *D*. *coniospora*, and presumably of other host genes that potentially help protect against infection and that are induced following a reduction in translation (Fig 10).

### DcEntB potentially interacts with many host proteins to affect diverse aspects of host cell physiology

In an attempt to understand better the complex effects of DcEntB we undertook the same type of biochemical approach as described above for DcEntA, in the hope of finding meaningful protein partners. We identified 121 proteins as being specifically enriched in the proteins co-immunoprecipitated with DcEntB (S5 Table). One of them, NST-1, is the orthologue of human GNL3L (G protein nucleolar 3 like), a nucleolar GTPase that is essential for ribosomal pre-rRNA processing and cell proliferation [82]. As mentioned above, many heat-labile enterotoxins exert their effects through ADP-ribosylation of guanine nucleotide-binding proteins. Among the other candidate DcEntB interactors that are predicted by NoD NoLS predictor [33] to be nucleolar proteins (S5 Table), PES-7/IQGAP, LET-60/HRas, RAB-6.1/RAB6A and CDC-42/CDC42 all have GTP binding or GTPase activity. Interestingly, the homologue of LET-502, the Rho-associated coiled-coil kinase, ROCK1, was recently shown to concentrate in the nucleolus during human cytomegalovirus infection [83]. Whether these different candidates are authentic interactors of DcEntB remains, however, to be established.

One of the consequences of DcEntB expression is an increase in *nlp-29p*::*GFP* reporter gene expression. In a previous genome-wide RNAi screen for this same phenotype, we identified close to 300 genes [43]. Among the corresponding proteins, only 2 (SEM-5 and K08E3.5) correspond to candidate DcEntB interactors. DcEntB, in common with DcEntA, also appears to inhibit translation. Several of its potential interactors (e.g. CIF-1 and IFG1, eukaryotic translation initiation factor orthologues) are essential components of the translational machinery. As none of these proteins are predicted to be nucleolar (S5 Table), the relevance of these observations remains to be established.

To take a broader view, we again used the enrichment tools in Wormbase. In contrast to the DcEntA interactors, there were few enriched classes. Among them, “nuclei enlarged” (WBPhenotype:0001567; p = 7.4x10^-4^) stood out, with both LET-502/ROCK and LET-60/HRas associated with this term ([84]; S5 Table). Given the alteration of nuclear size and nucleolar morphology provoked by DcEntB expression, these candidates merit further investigation. As knocking down the gene corresponding to one other candidate protein with this annotation, RNP-4, (orthologue of human RBM8A (RNA binding motif protein 8A) causes a block in AMP gene expression [43], understanding precisely the consequences of DcEntB expression will be challenging. It would be facilitated by identifying those host proteins that are not only interactors of DcEntB but also substrates for its enzymatic activity. For the time being, we did not find convincing evidence for specific ADP-ribosylation of any of the candidate enterotoxin interactors. ADP-ribose is a labile group that breaks easily with the fragmentation method used for the mass spectrometric analysis [85]. Nevertheless, the results reported here represent an important step in understanding the complexity of the molecular interactions that underlie *D*. *coniospora*’s capacity to infect and kill *C*. *elegans*.

## Discussion

The comprehension of fungal pathogenesis requires the identification of virulence factors and a dissection of their mode of action. In the current study, we chose to express individual candidate fungal virulence factors, as tagged chimeric proteins in a single tissue, the multi-nuclear epidermal syncytium hyp7. Tagging proteins can affect their sub-cellular localisation and/or function [86,87]. There has, however, been sufficient experience with fluorescent reporter proteins, in *C*. *elegans* and in other model organisms, including genome-wide surveys of protein localization [88], to know that in most cases the chimeric protein acts like its unmodified counterpart. Our approach is analogous to a recent study where numerous candidate secreted effector proteins from the plant pathogen *Colletotrichum higginsianum* were expressed as N-terminal fusions with GFP directly inside plant cells and determined to localize to peroxisomes, Golgi bodies, and microtubules [89]. As another example, a putative virulence factor from the nematode-trapping fungus *Duddingtonia flagrans* was expressed in *C*. *elegans* as a C-terminal GFP-fusion construct where it is localized to nuclei, consistent with the presence of an NLS in its sequence [90]. Among the *D*. *coniospora* proteins we studied here, DcEntB had a nucleolar localization, as predicted *in silico*. Generally, therefore, the expression pattern of a chimeric protein will reflect that of the native protein. In cases where more than one virulence factor act in a complex, however, expressing them individually may not be predictive of their behaviour during a natural infection. Further, the actions of some virulence factors may be antagonistic, as seen for the effect of DcEntA and DcEntB on AMP gene expression, so alone their effects will not reproduce the natural pathophysiology of infection. Nevertheless, when, as here, mutants are not available, heterologous expression can provide one route to understanding virulence factor function under conditions that are more physiological than *ex vivo* or *in vitro* systems.

Dozens of the *D*. *coniospora* proteins predicted to be secreted are lineage-specific [2] and presumable result from co-evolution with nematode hosts. They are of great interest for the understanding of evolutionary dynamics, for which *D*. *coniospora* is potentially a powerful model [11], but represent a major challenge due to the lack of any prior knowledge. Therefore, here, we chose to focus on enterotoxin genes that are expected to play a direct role in fungal virulence. We selected 3 from the expanded genomic repertoire of *D*. *coniospora* genes encoding enterotoxin α domain proteins. Expression of one of them, DcEntC was not associated with strong phenotypes, and did not reduce the lifespan of *C*. *elegans*. This indicates that the effects of DcEntA and DcEntB, which made worms sick and die precociously are specific. They presumably reflect the functions of their auxiliary protein domains. This was consistent also with their unique sets of potential host protein targets. Whether *DcEntC* expression impacts resistance to infection has yet to be established.

We observed that DcEntA, disrupted the actin cytoskeleton and the normal vesicular pattern of SNF-12, prevented the accumulation of STA-2 in the nucleus and blocked AMP gene expression. We recently demonstrated that upon wounding, microtubule rearrangement drives reorganisation of the actin cytoskeleton and is required for proper recruitment of SNF-12 to the injury site, as well as STA-2-dependent AMP gene expression [49]. Thus, DcEntA may inhibit AMP gene expression through an effect on SNF-12 localisation and STA-2 activity. Blocking key immune defence pathways is a strategy used by many pathogens across kingdoms [91–93].

The gene *ifas-1* is induced upon *D*. *coniospora* infection. We found that this is potentially a consequence of translation inhibition, and that suppressing STA-2 activity promoted *ifas-1* expression. While the precise pathway that positively regulates *ifas-1* is currently unknown, this effect could be seen as a fail-safe surveillance mechanism, whereby fungal interference with a major defence pathway leads to a boost of a complementary defence mechanism.

Both DcEntA and DcEntB appear to reduce translation. Many enterotoxin α proteins ADP-ribosylate elongation factor family (EF2) proteins essential for ribosome function. Notably, Exotoxin A from *P*. *aeruginosa* targets EF2, thereby blocking protein synthesis in *C*. *elegans* intestinal epithelial cells [57,58]. It is possible that DcEntA and DcEntB also act at the translational level, impacting host defence protein expression as, while not finding EF2 proteins, we noted several eukaryotic Initiation Factor (eIF) proteins among the specific interactors of both DcEntA and DcEntB. While DcEntA decreases STA-2 levels in the nucleus, which has the potential to boost *ifas-1* expression, DcEntB increases them, potentially explaining why it does not induce *ifas-1* expression (Fig 10).

Interestingly, given the pattern of SNF-12 observed upon expression of DcEntA, with an enrichment along the baso-lateral seam cell boundary, 38 of the high-confidence DcEntA interactors are known to be potential binding partners of DLG-1, which is found in the same location [75]. We also identified 11 proteins reported to interact with AKIR-1, which is essential for AMP gene expression in hyp7 [42], suggesting an additional way in which DcEntA might affect the host response. Together our results indicate that DcEntA affects, directly and indirectly, host defence protein expression, by targeting different cellular processes. This is not unusual for virulence factors, with, for example, EspF from enteropathogenic and enterohemorrhagic *Escherichia coli* described as a “bacterial pathogen’s Swiss army knife” because of the diversity of its actions [94]. Further study will be required to validate the many candidate host protein interactors and to determine whether any have preponderant roles in pathogenesis during a normal infection.

The effect of DcEntB expression was also complex. One prominent consequence was an increase in AMP gene expression, dependent on the canonical STA-2 pathway. DcEntB was concentrated in the nucleolus, a dynamic sub-nuclear organelle for ribosomal RNA (rRNA) biogenesis that acts as a cellular stress sensor [95]. For example, impairment of nucleolar function is thought to stabilize p53, a key regulator of cellular homeostasis [96], while inhibition of proteasome activity leads to sequestration of p53 proteins to the nucleolus [97]. In *C*. *elegans*, the p53 homologue, CEP-1, acts downstream of NOL-6, a nucleolar RNA-associated protein (NRAP), via its transcriptional target SYM-1 to enhance resistance to bacterial infection [80]. More recently, it was shown that infection by *P*. *aeruginosa* decreases the level of the nucleolar pre-rRNA processing protein fibrillarin, FIB-1 [79]. FIB-1 acts downstream of the BRAT/TRIM2 homologue, NCL-1, to regulate rRNA abundance and nucleolar size [37]. Bacterial infection therefore decreases rRNA and nucleolar size [79]. For the moment, no *P*. *aeruginosa* effector has been found to localise specifically to the host nucleolus. Indeed, it was only comparatively recently that examples of bacterial effectors that target the nucleolus were identified, the first being EspF [98]. Here, we found that DcEntB is recruited to nucleoli and can make them larger and irregularly shaped. This is also one of the consequences of infection of the epidermis by *D*. *coniospora*, but whether this depends solely on the action of DcEntB remains to be established. The relationship between the change in nucleolar morphology and the expression of AMP genes appears complex. On the one hand, blocking protein translation, which alters the nucleolus, was associated with a small and specific increase in *nlp* AMP gene expression. On the other, knocking down *sta-2* expression blocked the elevated AMP gene expression induced by DcEntB and reverted nucleoli almost to normal. Further work will be needed to tease out the underlying causal links.

Regardless, this induction of AMPs could be interpreted as a type of surveillance immunity. Potentially, the changes in cellular physiology provoked by DcEntB could be detected as a damage signal and induce an immune response in the epidermis (Fig 10). Several other examples illustrate the important role of surveillance immunity in *C*. *elegans* (reviewed in [99]). In one case, Stx1, a virulence factor from enterohemorrhagic *E*. *coli* that is able to inhibit protein synthesis, activates the intestinal p38 MAPK pathway [100]. Apart from the core MAPK cassette that is shared between epidermis and intestine, the p38 pathway has distinct inputs and outputs in the two tissues (reviewed in [101]). It has been shown, however, that there is an intimate balance of MAPK activity between the 2 tissues, with stimulation of the p38 pathway in one negatively influencing its activity in the other [43], via a mechanism potentially involving the Tribbles homologue NIPI-3 [41,102]. Thus, while we expect virulence factors like DcEntA and DcEntB to act in a cell autonomous manner, during the course of an infection, they also have the potential to influence immune defences in distant tissues. During infection, as mycelia spread, virulence factors would be secreted into different tissues, and potentially into the pseudocoelom. This further complicates any understanding of the dynamic host response to natural infection.

It is interesting to note that in contrast to DcEntA that has a unique C-terminal domain not found in any other protein, DcEntB has orthologues in many pathogenic fungi. These include in nematode-trapping fungi *Dactylellina* spp. [103] and *Drechslerella* spp. [104], as well as the egg-infecting species *Pochonia chlamydosporia* [105]. The different species represent distinct branches on the phylogenetic tree, reflecting the multiple independent origins of nematode parasitism [2]. Orthologues are also found in ant-infecting *Ophiocordyceps* species [106]. It has previously been suggested that heat-labile enterotoxins are important effectors in host adaptation and co-evolution [107]. It is possible that as a more ancient virulence factor, *C*. *elegans* has been able to develop a counter-defensive strategy against DcEntB. This would then potentially drive enterotoxin diversification in *D*. *coniospora*, leading to the emergence of DcEntA. We hypothesise that *C*. *elegans* has not yet evolved an effective defence strategy against this more recent virulence factor. It will clearly be important in the future to assay the expression of the different enterotoxin genes, measure the levels of the corresponding proteins in *C*. *elegans* during an infection, and to gauge their relative importance in pathogenesis, for example by co-expressing more than one factor at a time, all of which is beyond the scope of the current study.

In conclusion, through this initial investigation of *D*. *coniospora* virulence factors, we have revealed the very complicated, sometimes antagonistic, nature of some of the molecular interactions that come into play during natural fungal infection of *C*. *elegans*. In addition to providing insight into the molecular function of two representative enterotoxins, we have gained a new understanding of host defence mechanisms.

## Supporting information

S1 Fig (**A**, **B**) Schematic representation of different culture and selection procedures. (**A**) Transgenic worms carrying *rps-0p*::*hygR* conferring hygromycin resistance together with *unc-122p*::*GFP* as an extrachromosomal array (IG1864) were grown on NGM plates supplemented with hygromycin (left) or on standard NGM plates after manual selection on the basis of the expression of the fluorescent marker. (**B**) IG1864 worms were cultured overnight in liquid in the presence (left) or absence of hygromycin. Worms were transferred to NGM plates and in the latter case selected manually, as above. (**C**) Quantitative RT-PCR analysis comparing the expression of *irg-1* in IG1864 worms selected by growth on hygromycin-supplemented NGM plates to that in worms selected manually (-), and in IG1864 worms selected by synchronization in the presence of hygromycin to that in worms selected manually following synchronization in the absence of hygromycin (+). The results from 2 independent experiments are shown. It can be seen that even in worms that are resistant to hygromycin, prolonged culture in the presence of the antibiotic increases *irg-1* expression, while overnight exposure during early development does not.
(PDF)

S2 Fig Visualisation of *D*. *coniospora* genomes highlighting enterotoxin genes. A Circos plot [108] showing the positions of the predicted enterotoxin genes in the genomes of 2 *D*. *coniospora* strains. The full isolation history of the strains Swe2 (left hand side, derived from ATCC 96282 [2]) and Dan2 (right hand side, ARSEF 6962 [109]) are given elsewhere [11]. Orthologous gene pairs, with their corresponding Genbank protein identifiers, are joined by lines, coloured on the basis of the position on Swe2 chromosomes. The 3 enterotoxins characterised in the present study are shown in orange and the 4 Dan2 specific enterotoxin genes are shown in grey. The 4 Swe2 genes with an asterisk were missing from the original gene prediction [2] and were identified by manual curation. The Dan2 gene marked with the asterisk was not originally predicted to encode an enterotoxin [109], but removal of its unique intron gives rise to a *bona fide* enterotoxin. The Genbank identifiers for the Dan2 chromosomal sequences are shown. Swe2 chromosome 1 is the concatenation of JYHR01000002.1—JYHR01000004.1; chromosome 2 of JYHR01000001.1—JYHR01000007.1; chromosome 3 of JYHR01000008.1—JYHR01000009.1—JYHR01000005.1—JYHR01000006.1—JYHR01000003.1- JYHR010000011.1—JYHR010000010.1. The numbers on the outside of each chromosome indicate length in Mb. The pattern of gene reorganisation matches exactly the pattern of global chromosomal rearrangements seen between Swe2 and Dan2 [11].
(PDF)

S3 Fig (**A**) Expression of enterotoxin genes from the two *D*. *coniospora* strains. We re-analysed the available RNAseq datasets for Swe2 and Dan2, to compare expression for the enterotoxin genes from the original Swe2 gene prediction [2] and their Dan2 orthologues. The log_10_ of the maximal value, expressed as fragments per Kb of transcript per million mapped reads (FKPM), among the different conditions for each strain is shown. Genes are ranked according to their relative expression in Swe2. DcEntC, DcEntA, DcEntB (from top to bottom) are highlighted in grey. The values for the previously studied gene SapA [2], highlighted in yellow, are shown for comparison. (**B**) Sequence relationships between the Swe2 enterotoxins. A phylogenetic tree depicting the deduced relationship between all 23 Swe2 enterotoxins. The tree is rooted in its midpoint. Branch confidence is shown for the inner nodes. The scale bar indicates the line length corresponding to one substitution per site.
(PDF)

S4 Fig Quantitative RT-PCR analysis of the relative expression of mKate2 in worms expressing DcEntA (IG1926), or DcEntC (IG1880), relative to those expressing DcEntB (IG1925). (PDF)

S5 Fig (**A**) Representative images (left panels: white light; right panels: red fluorescence) of two day adult worms expressing DcEntA in a wild-type (IG2043; lower panels) or GPA-12* (IG1926; upper panels) background. Scale bar, 50 μm. (**B**) Quantification of the ratio of relative red fluorescence to size (TOF), or of TOF alone (left and right panels, respectively) of two day adult worms expressing DcEntA in a wild-type (IG2043) or GPA-12* (IG1926) background. (**C**) Lifespan counted from the L4 stage at 25°C of control (*hygR;frIs7* IG1864) worms and worms carrying *frIs7* expressing DcEntA (IG1942), GPA-12* (IG1389), or GPA-12* and DcEntA (GPA-12*; DcEntA IG1948). For each strain, n = 50. **** p < 0.0001, one-sided log rank test. Representative of 2 independent biological replicates. (**D**) Quantitative RT-PCR analysis of the expression of *ifas-1* in the same 4 strains. Data from two independent experiments are shown. The decrease in *ifas-1* expression in IG1389 compared to IG1864 is consistent with previous results [5]. The fold-change in expression level between the 2 indicated conditions is significantly different; * p < 0.05, paired one-sided t test.
(PDF)

S6 Fig (**A**) Confocal images of young adult worms expressing STA-2::GFP (XW18234) without infection (NI) or 18h after infection (Infec) with *D*. *coniospora*. Scale bar 20 μm. (**B**) The relative fluorescence intensity of STA-2::GFP in the nucleus under the same conditions; n = 51 (NI) and 68 (Infec). (**C**) Ratio of the expression of *nlp* and *cnc* gene expression in infected to non-infected worms, measured by quantitative RT-PCR analysis, assayed from the same samples. (**D, E**) Results for an independent biological replicate for the experiments shown in (**B**) and (**C**), respectively; n = 67 (NI) and 74 (Infec). *** p < 0.001, **** p < 0.0001, Mann-Whitney test.
(PDF)

S7 Fig Representative pairs of images (left, white light; right, green fluorescence) of adult *atf-4p(uORF)*::*GFP* reporter worms (LD1499) after 7 h (bottom left 2 panels) and 24 h (bottom right 2 panels) of infection with *D*. *coniospora*, or aged matched non-infected worms (NI, top 4 panels). Scale bar, 200 μm.
(PDF)

S8 Fig Quantitative RT-PCR analysis of the expression of *hsp-4*, *hsp-6*, *hsp-60*, *gst-4* and *gpdh-1* in worms expressing DcEntA (IG1926), DcEntB (IG1925) or DcEntC (IG1880). Results are presented relative to control worms (JDW141).
(PDF)

S9 Fig HCD fragment mass spectrum of peptide sequence TAGASNWIANK, identifying asparagine N275 as a mono-ADP-ribosylation (MAR) site of DcEntA. The generated fragment ions matched to the theoretical mass spectrum of the peptide are marked as b-ions (the product when the charge is retained on the N-terminus; blue), y-ions (when the charge is retained on the C-terminus; red; spanning the MAR-modified residue) and their corresponding ions with a neutral loss (H_2_O or NH_3_; gold). The positions of fragmentation are shown in the inset peptide sequence. Diagnostic ions (pink) of AMP and ADP were generated by breakage of the MAR group during HCD fragmentation.
(PDF)

S10 Fig (**A**) Representative confocal images of hyp7 nuclei in young adult worms expressing FIB-1::GFP with (DcEntB, IG1984; lower two panels) or without (Control; IG1596; upper two panels) DcEntB. (**B**) Representative confocal images of hyp7 nuclei in young adult worms expressing FIB-1::GFP and DcEntB (IG1984) on *sta-1* (upper two panels) or *sta-2* (lower two panels) RNAi. Only the green channel is shown. Scale bar, 5 μm.
(PDF)

S11 Fig (**A**) Representative confocal images of hyp7 nuclei in young adult IG1596 worms expressing FIB-1::GFP after 24 h infection as young adults at 25°C (Infected; lower two panels) or in uninfected animals (Control; upper two panels). (**B**) Representative confocal images of hyp7 nuclei in young adult IG1596 worms expressing FIB-1::GFP after 6 h CHX exposure (CHX; lower two panels) or without CHX exposure (Control; upper two panels). Only the green channel is shown. Scale bar, 5 μm.
(PDF)

S1 Table Full genotypes of transgenic strains. (DOCX)

S2 Table Oligonucleotide primers. (DOCX)

S3 Table Phenotypes of transgenic worms expressing one of the 3 candidate virulence factors. (DOCX)

S4 Table Identification of protein-protein interactors for DcEntA. The first sheet gives quantitative summary statistics for the significant and specific candidate protein-protein interactors for DcEntA obtained from results for analyses of 3 independent samples, referenced to Wormbase release WS275. The subsequent sheets report annotations and gene enrichments using Wormbase tools and WS277. The GeneIDs of the candidate interactors did not evolve between WS275 and WS277.
(XLSX)

S5 Table Identification of protein-protein interactors for DcEntB. The first sheet gives quantitative summary statistics for the significant and specific candidate protein-protein interactors for DcEntB obtained from results for analyses of 3 independent samples, referenced to Wormbase release WS275. The subsequent sheets report annotations and gene enrichments using Wormbase tools and WS277. The GeneIDs of the candidate interactors did not evolve between WS275 and WS277.
(XLSX)
