# Supplementary figures and images for: Antagonistic fungal enterotoxins intersect at multiple levels with host innate immune defences
Source: PLoS Genet. 2021 Jun 24;17(6):e1009600. doi: 10.1371/journal.pgen.1009600 (PMC8263066; doi:10.1371/journal.pgen.1009600)

**A**

No synchronization in liquid

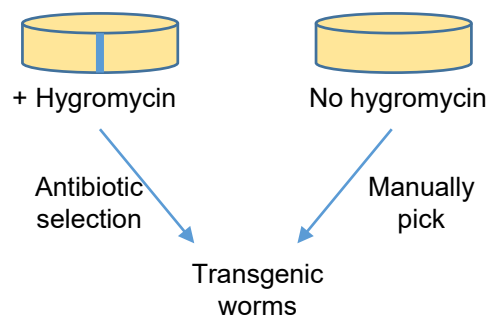**B**

Synchronization in liquid

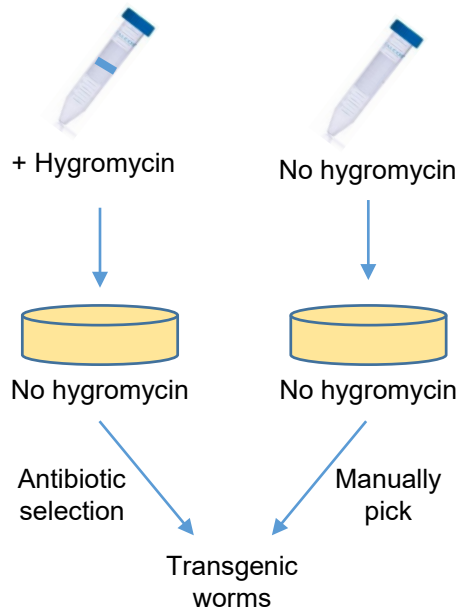**C**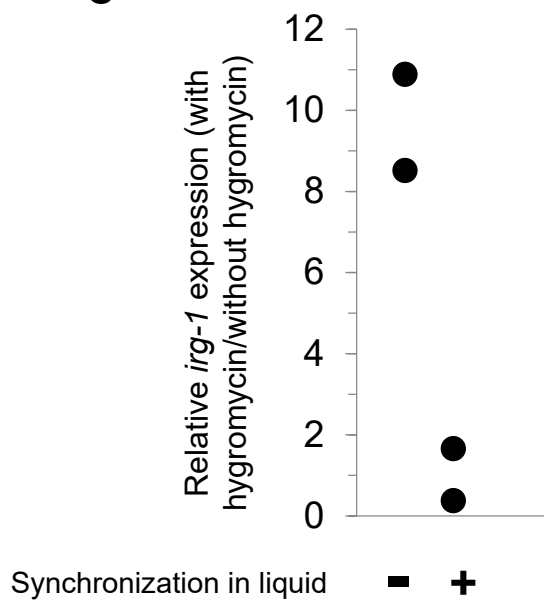

Supplement: S1 Fig — (A, B) Schematic representation of different culture and selection procedures. (A) Transgenic worms carrying rps-0p::hygR conferring hygromycin resistance together with unc-122p::GFP as an extrachromosomal array (IG1864) were grown on NGM plates supplemented with hygromycin (left) or on standard NGM plates after manual selection on the basis of the expression of the fluorescent marker. (B) IG1864 worms were cultured overnight in liquid in the presence (left) or absence of hygromycin. Worms were transferred to NGM plates and in the latter case selected manually, as above. (C) Quantitative RT-PCR analysis comparing the expression of irg-1 in IG1864 worms selected by growth on hygromycin-supplemented NGM plates to that in worms selected manually (-), and in IG1864 worms selected by synchronization in the presence of hygromycin to that in worms selected manually following synchronization in the absence of hygromycin (+). The results from 2 independent experiments are shown. It can be seen that even in worms that are resistant to hygromycin, prolonged culture in the presence of the antibiotic increases irg-1 expression, while overnight exposure during early development does not. (PDF) [file pgen.1009600.s001.pdf]

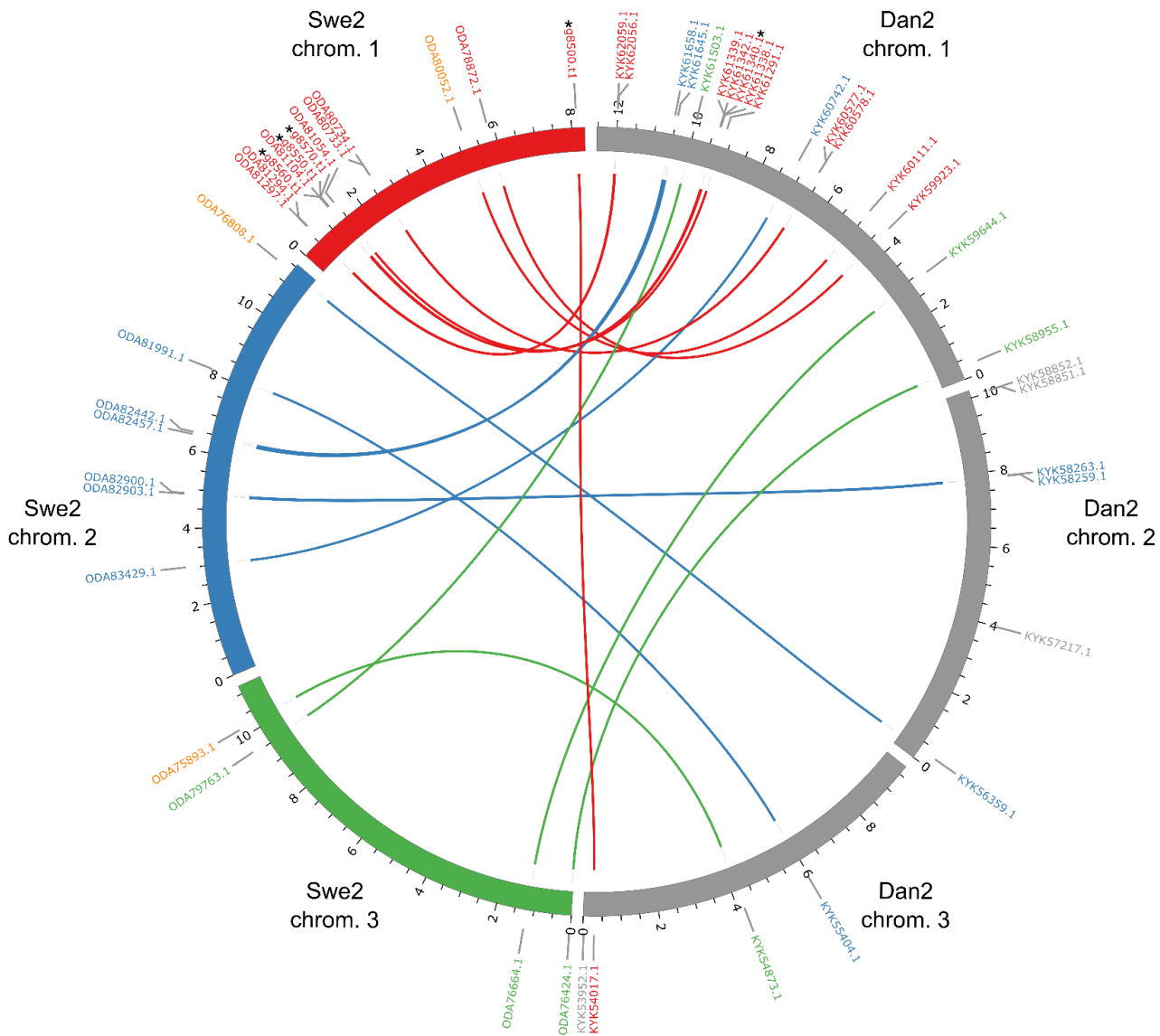

Supplement: S2 Fig — A Circos plot [108] showing the positions of the predicted enterotoxin genes in the genomes of 2 D. coniospora strains. The full isolation history of the strains Swe2 (left hand side, derived from ATCC 96282 [2]) and Dan2 (right hand side, ARSEF 6962 [109]) are given elsewhere [11]. Orthologous gene pairs, with their corresponding Genbank protein identifiers, are joined by lines, coloured on the basis of the position on Swe2 chromosomes. The 3 enterotoxins characterised in the present study are shown in orange and the 4 Dan2 specific enterotoxin genes are shown in grey. The 4 Swe2 genes with an asterisk were missing from the original gene prediction [2] and were identified by manual curation. The Dan2 gene marked with the asterisk was not originally predicted to encode an enterotoxin [109], but removal of its unique intron gives rise to a bona fide enterotoxin. The Genbank identifiers for the Dan2 chromosomal sequences are shown. Swe2 chromosome 1 is the concatenation of JYHR01000002.1—JYHR01000004.1; chromosome 2 of JYHR01000001.1—JYHR01000007.1; chromosome 3 of JYHR01000008.1—JYHR01000009.1—JYHR01000005.1—JYHR01000006.1—JYHR01000003.1- JYHR010000011.1—JYHR010000010.1. The numbers on the outside of each chromosome indicate length in Mb. The pattern of gene reorganisation matches exactly the pattern of global chromosomal rearrangements seen between Swe2 and Dan2 [11]. (PDF) [file pgen.1009600.s002.pdf]

A

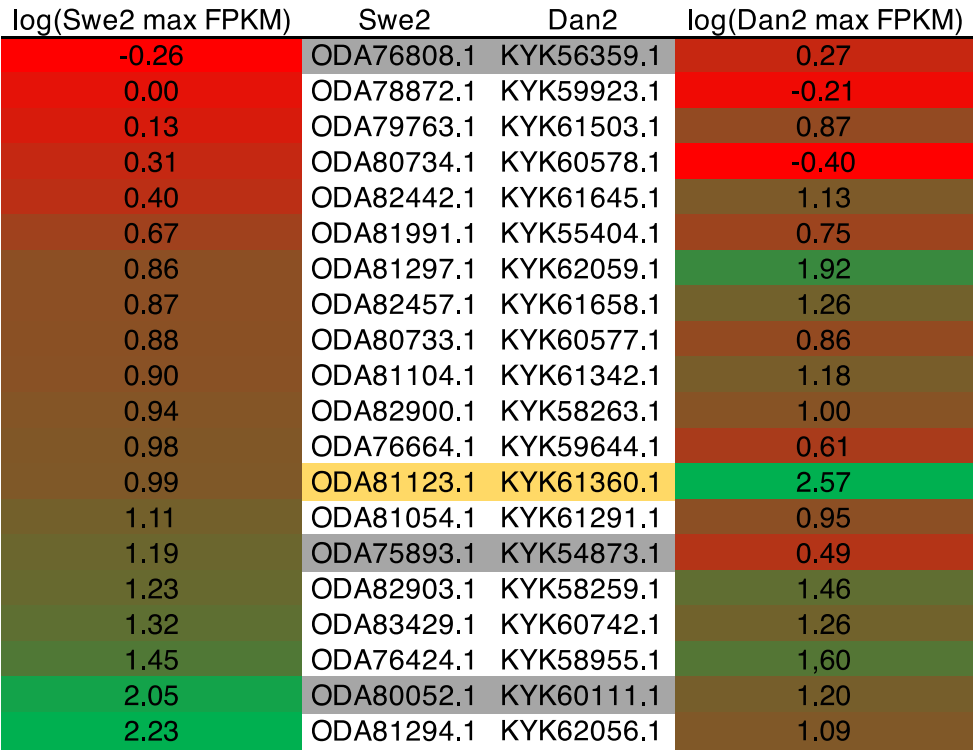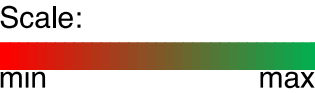

B

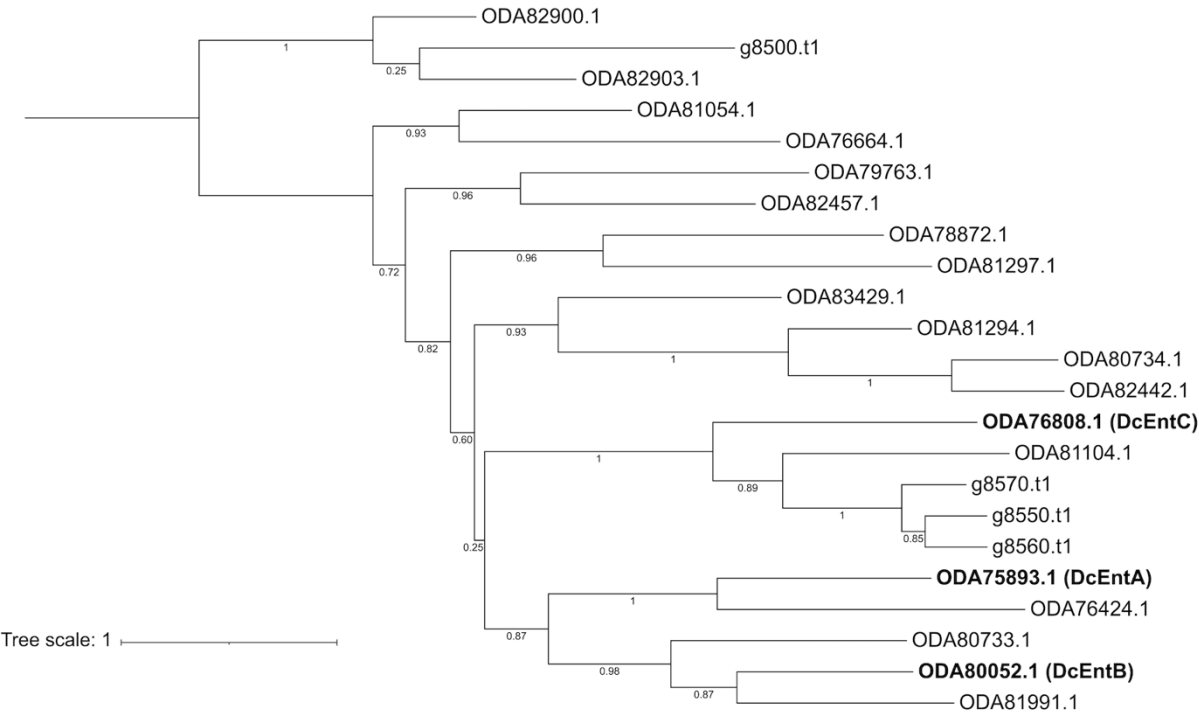

Supplement: S3 Fig — (A) Expression of enterotoxin genes from the two D. coniospora strains. We re-analysed the available RNAseq datasets for Swe2 and Dan2, to compare expression for the enterotoxin genes from the original Swe2 gene prediction [2] and their Dan2 orthologues. The log10 of the maximal value, expressed as fragments per Kb of transcript per million mapped reads (FKPM), among the different conditions for each strain is shown. Genes are ranked according to their relative expression in Swe2. DcEntC, DcEntA, DcEntB (from top to bottom) are highlighted in grey. The values for the previously studied gene SapA [2], highlighted in yellow, are shown for comparison. (B) Sequence relationships between the Swe2 enterotoxins. A phylogenetic tree depicting the deduced relationship between all 23 Swe2 enterotoxins. The tree is rooted in its midpoint. Branch confidence is shown for the inner nodes. The scale bar indicates the line length corresponding to one substitution per site. (PDF) [file pgen.1009600.s003.pdf]

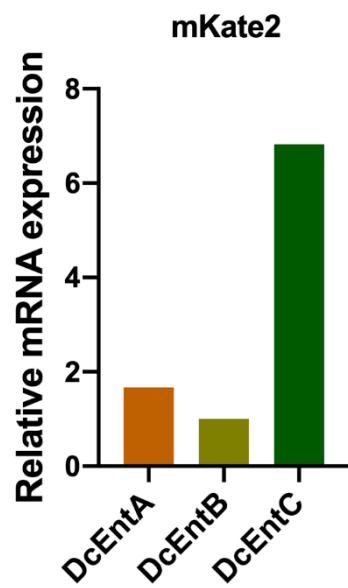

Supplement: S4 Fig — (PDF) [file pgen.1009600.s004.pdf]

**A**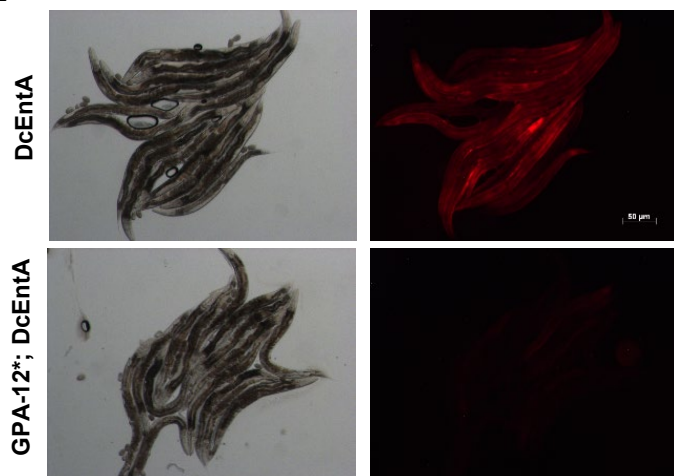**B**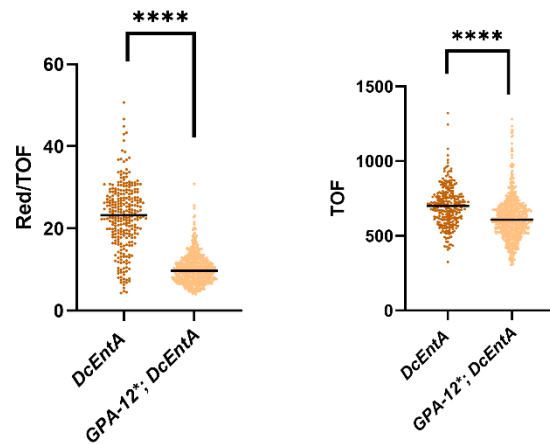**C**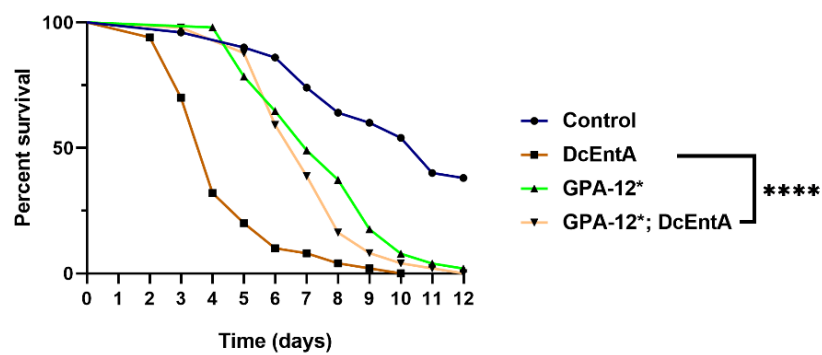**D**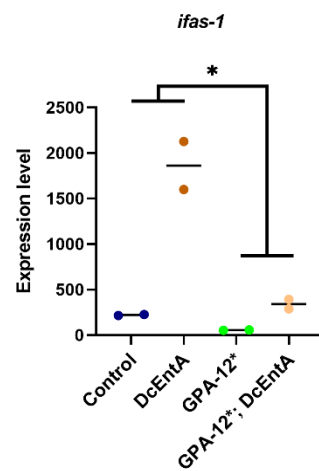

Supplement: S5 Fig — (A) Representative images (left panels: white light; right panels: red fluorescence) of two day adult worms expressing DcEntA in a wild-type (IG2043; lower panels) or GPA-12* (IG1926; upper panels) background. Scale bar, 50 μm. (B) Quantification of the ratio of relative red fluorescence to size (TOF), or of TOF alone (left and right panels, respectively) of two day adult worms expressing DcEntA in a wild-type (IG2043) or GPA-12* (IG1926) background. (C) Lifespan counted from the L4 stage at 25°C of control (hygR;frIs7 IG1864) worms and worms carrying frIs7 expressing DcEntA (IG1942), GPA-12* (IG1389), or GPA-12* and DcEntA (GPA-12*; DcEntA IG1948). For each strain, n = 50. **** p < 0.0001, one-sided log rank test. Representative of 2 independent biological replicates. (D) Quantitative RT-PCR analysis of the expression of ifas-1 in the same 4 strains. Data from two independent experiments are shown. The decrease in ifas-1 expression in IG1389 compared to IG1864 is consistent with previous results [5]. The fold-change in expression level between the 2 indicated conditions is significantly different; * p < 0.05, paired one-sided t test. (PDF) [file pgen.1009600.s005.pdf]

**A**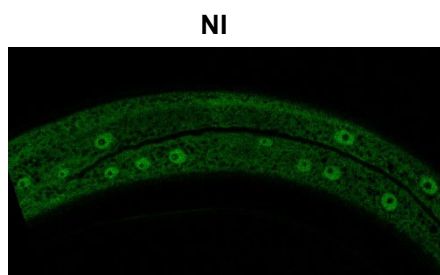**Infec**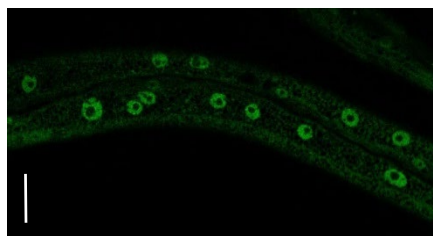**B**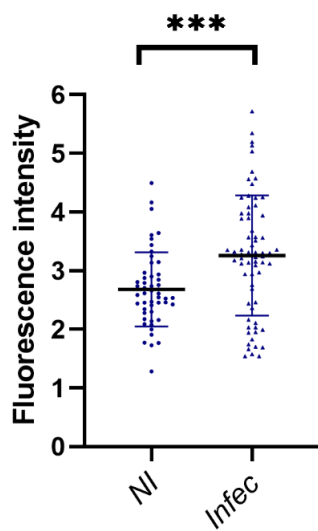**C**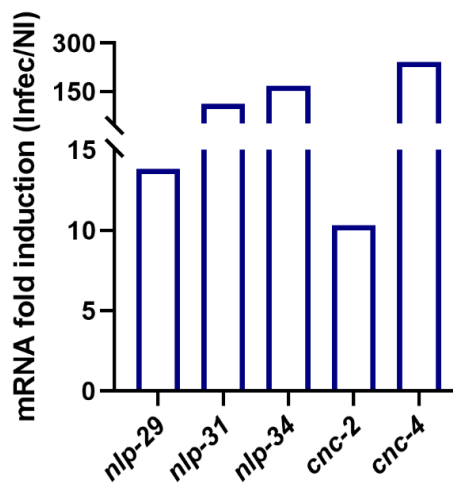**D**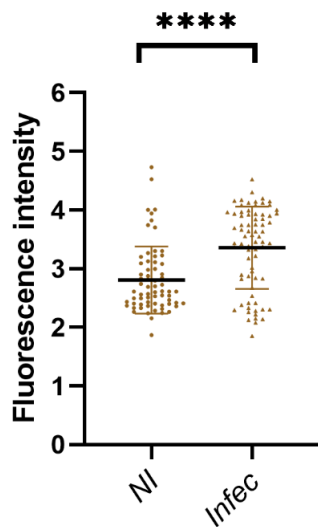**E**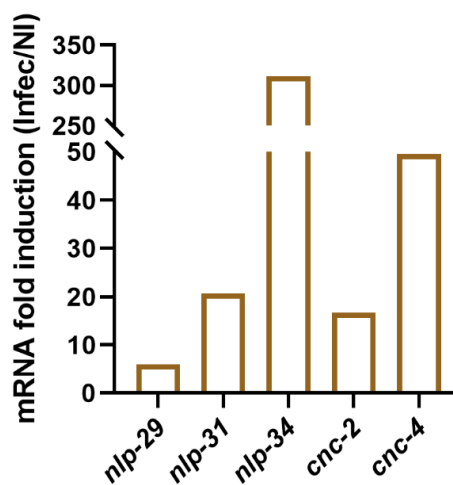

Supplement: S6 Fig — (A) Confocal images of young adult worms expressing STA-2::GFP (XW18234) without infection (NI) or 18h after infection (Infec) with D. coniospora. Scale bar 20 μm. (B) The relative fluorescence intensity of STA-2::GFP in the nucleus under the same conditions; n = 51 (NI) and 68 (Infec). (C) Ratio of the expression of nlp and cnc gene expression in infected to non-infected worms, measured by quantitative RT-PCR analysis, assayed from the same samples. (D, E) Results for an independent biological replicate for the experiments shown in (B) and (C), respectively; n = 67 (NI) and 74 (Infec). *** p < 0.001, **** p < 0.0001, Mann-Whitney test. (PDF) [file pgen.1009600.s006.pdf]

NI

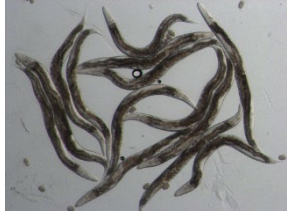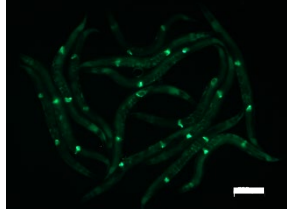

NI

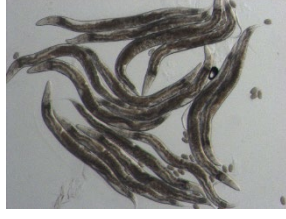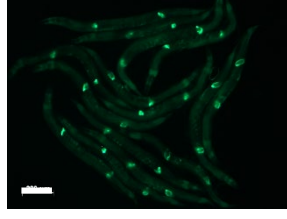

Infec 7h

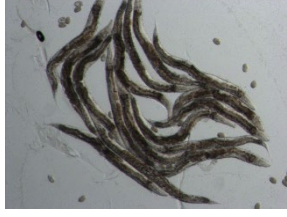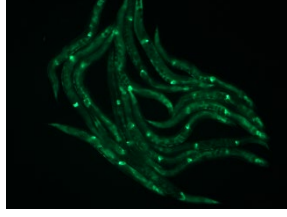

Infec 24h

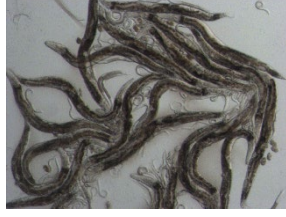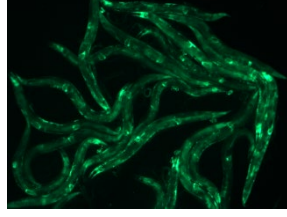

Supplement: S7 Fig — Representative pairs of images (left, white light; right, green fluorescence) of adult atf-4p(uORF)::GFP reporter worms (LD1499) after 7 h (bottom left 2 panels) and 24 h (bottom right 2 panels) of infection with D. coniospora, or aged matched non-infected worms (NI, top 4 panels). Scale bar, 200 μm. (PDF) [file pgen.1009600.s007.pdf]

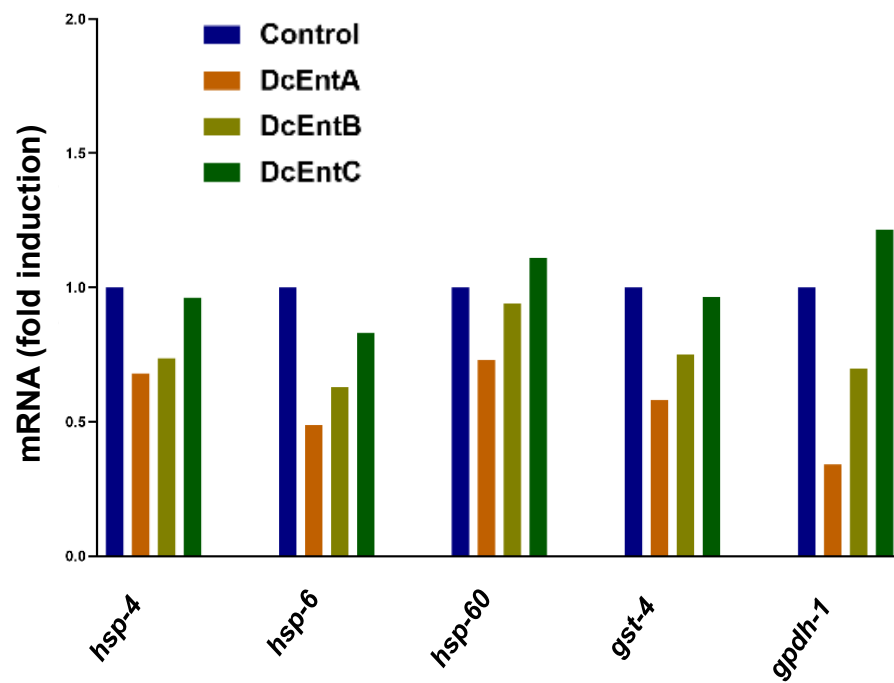

Supplement: S8 Fig — Quantitative RT-PCR analysis of the expression of hsp-4, hsp-6, hsp-60, gst-4 and gpdh-1 in worms expressing DcEntA (IG1926), DcEntB (IG1925) or DcEntC (IG1880). Results are presented relative to control worms (JDW141). (PDF) [file pgen.1009600.s008.pdf]

Scan 40192 Method FTMS; HCD Score 78.82 m/z 837.82

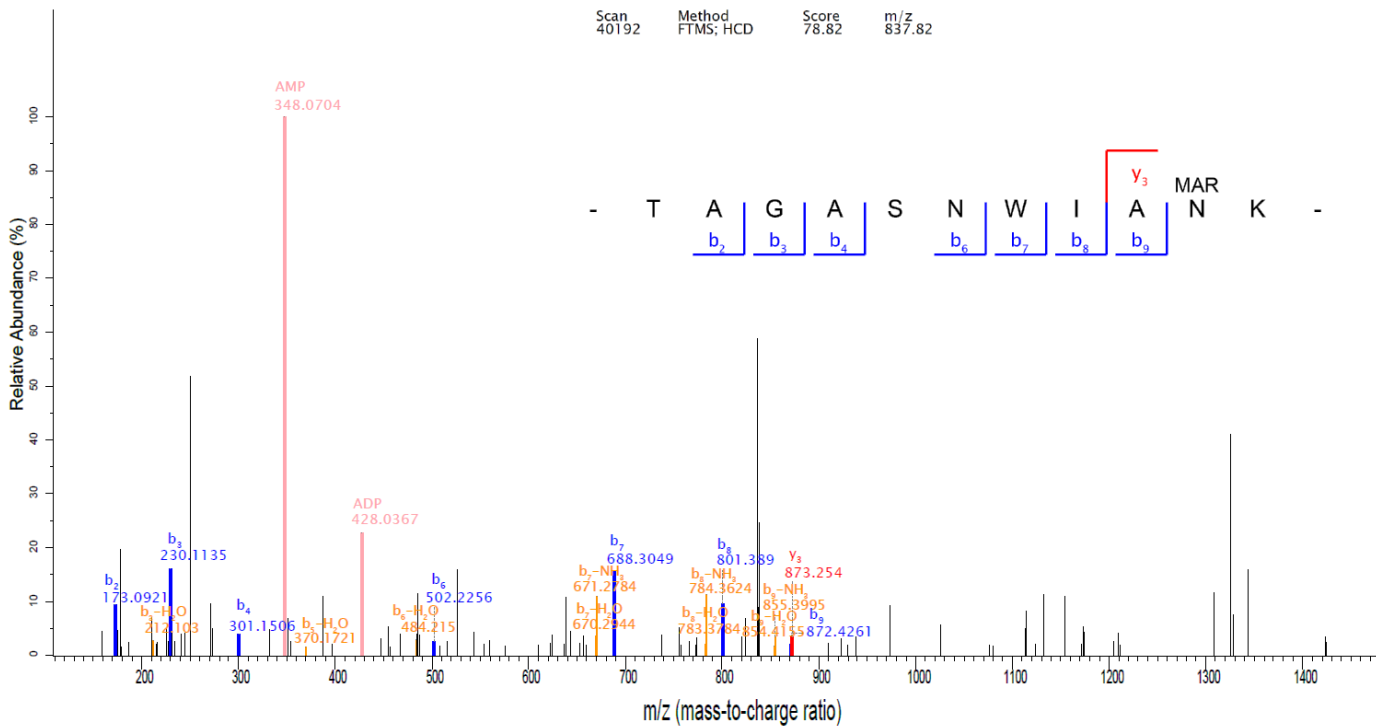

Supplement: S9 Fig — HCD fragment mass spectrum of peptide sequence TAGASNWIANK, identifying asparagine N275 as a mono-ADP-ribosylation (MAR) site of DcEntA. The generated fragment ions matched to the theoretical mass spectrum of the peptide are marked as b-ions (the product when the charge is retained on the N-terminus; blue), y-ions (when the charge is retained on the C-terminus; red; spanning the MAR-modified residue) and their corresponding ions with a neutral loss (H2O or NH3; gold). The positions of fragmentation are shown in the inset peptide sequence. Diagnostic ions (pink) of AMP and ADP were generated by breakage of the MAR group during HCD fragmentation. (PDF) [file pgen.1009600.s009.pdf]

**A**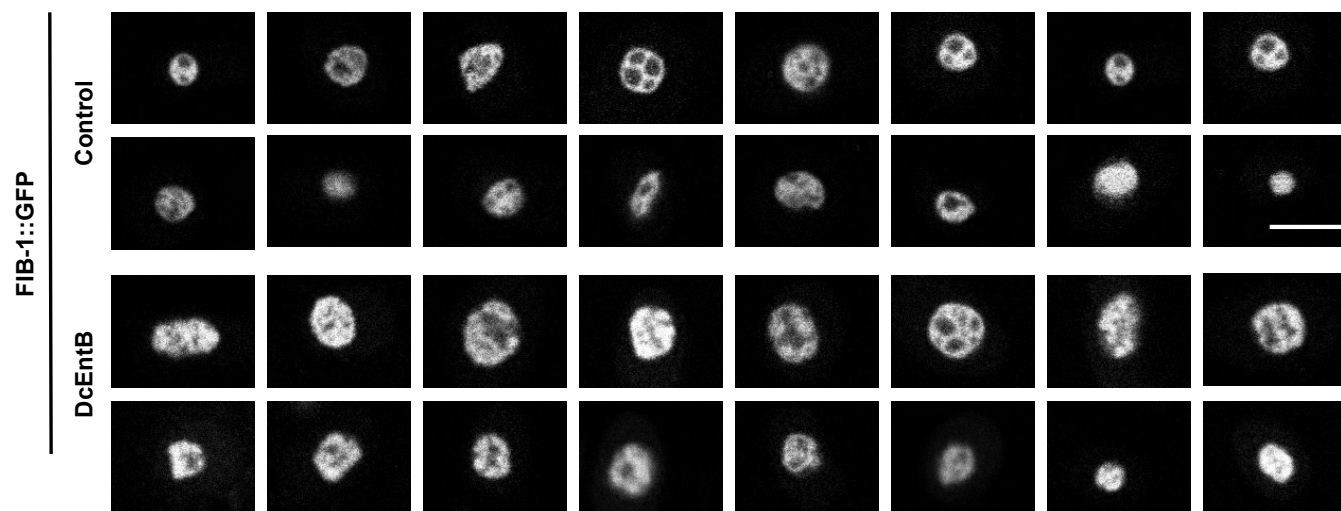**B**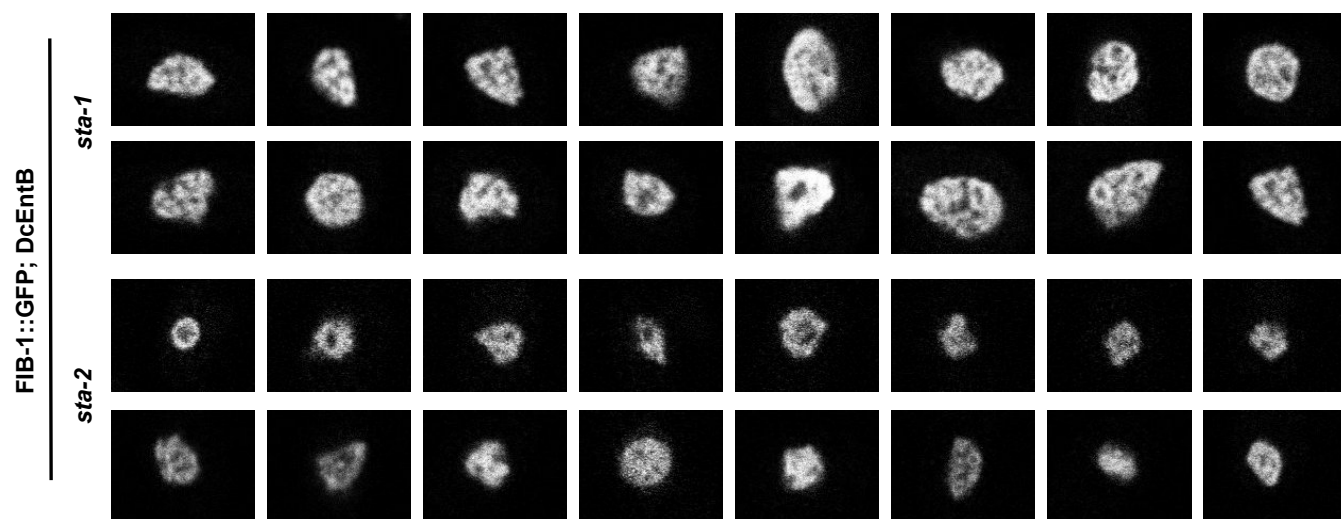

Supplement: S10 Fig — (A) Representative confocal images of hyp7 nuclei in young adult worms expressing FIB-1::GFP with (DcEntB, IG1984; lower two panels) or without (Control; IG1596; upper two panels) DcEntB. (B) Representative confocal images of hyp7 nuclei in young adult worms expressing FIB-1::GFP and DcEntB (IG1984) on sta-1 (upper two panels) or sta-2 (lower two panels) RNAi. Only the green channel is shown. Scale bar, 5 μm. (PDF) [file pgen.1009600.s010.pdf]

**A**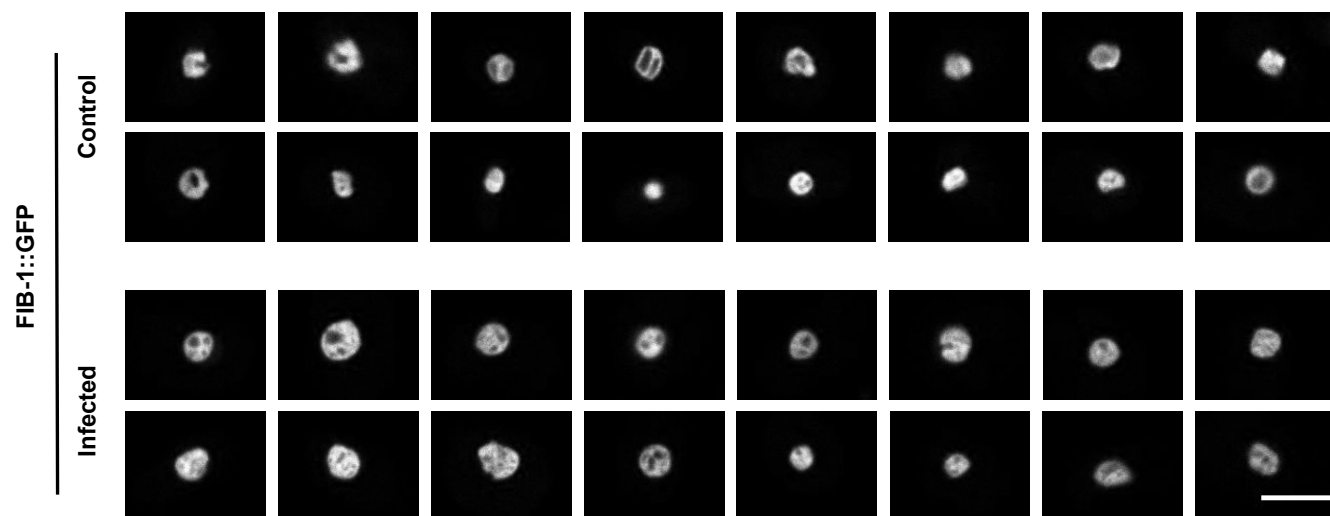**B**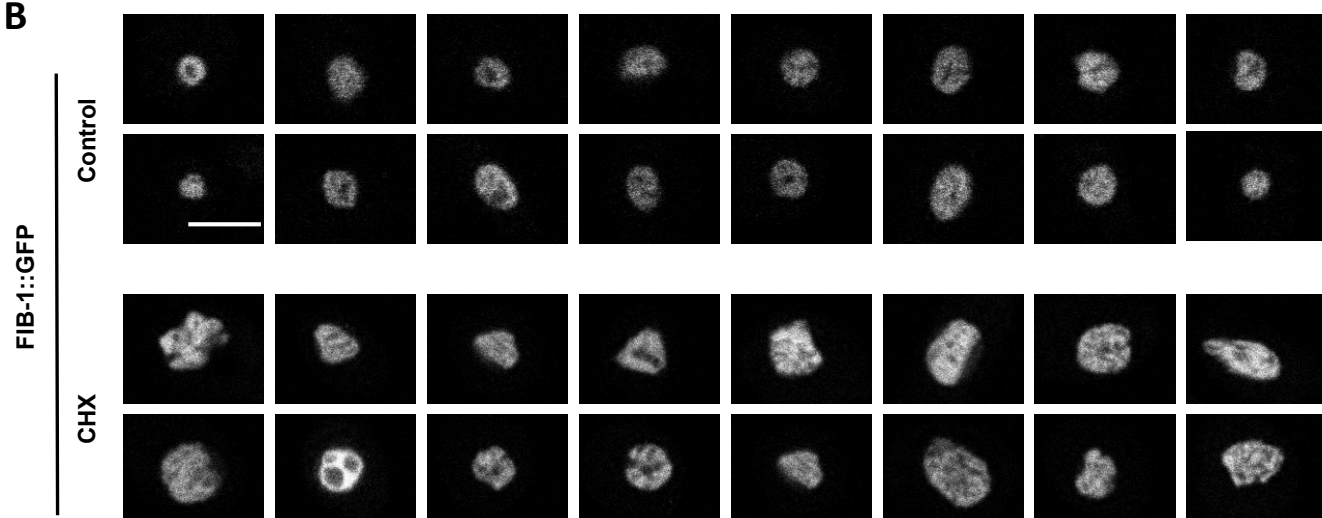

Supplement: S11 Fig — (A) Representative confocal images of hyp7 nuclei in young adult IG1596 worms expressing FIB-1::GFP after 24 h infection as young adults at 25°C (Infected; lower two panels) or in uninfected animals (Control; upper two panels). (B) Representative confocal images of hyp7 nuclei in young adult IG1596 worms expressing FIB-1::GFP after 6 h CHX exposure (CHX; lower two panels) or without CHX exposure (Control; upper two panels). Only the green channel is shown. Scale bar, 5 μm. (PDF) [file pgen.1009600.s011.pdf]
