# Supplementary material for: Antagonistic fungal enterotoxins intersect at multiple levels with host innate immune defences
Source: PLoS Genet. 2021 Jun 24;17(6):e1009600. doi: 10.1371/journal.pgen.1009600 (PMC8263066; doi:10.1371/journal.pgen.1009600)
Supplement: S1 Table — (DOCX) [file pgen.1009600.s012.docx]

Table S1. Full genotypes of transgenic strains.

| **Strain Name** | **Genotype** | **Reference** |
| --- | --- | --- |
| IG274 | *frIs7[nlp-29p::GFP, col-12p::DsRed] IV* | [1] |
| IG1389 | *frIs7[nlp-29p::GFP, col-12p::DsRed] IV; frIs30[(col-19p::GPA-12gf), pNP21(unc-53pB::GFP)] I* | [2] |
| IG1864 | *frIs7[nlp-29p::GFP, col-12p::DsRed] IV; frEx613[unc-122p::GFP, rps-0p::HygR]* | This study |
| JDW141 | *wrdSi23[eft-3p::TIR::P2A::BFP-NLS-degron:_3'tbb-2, ttTi4348] I; oxSi1091[mex-5p::Cas9(smu-2 introns) unc-119+] II; unc-119(ed3) III* | [3] |
| IG823 | *frIs43[col-12p::SNF-12::GFP, ttx-3p::DsRed2] V* | [4] |
| XW18234 | *qxSi727[sta-2p::sfGFP::STA-2; ttTi5605] II* | **[5]** |
| BPW24 | *pmk-1(miy[PMK-1(D327E)]; frIs7[nlp-29p::GFP, col-12p::DsRed] IV* | [6] |
| SJL1 | *cguIs1[fib-1p::FIB-1::GFP::fib-1_3'UTR]* | [7] |
| PX627 | *fxIs1[pie-1p::TIR1::mRuby, I:2851009]) I; spe-44(fx110[spe-44::degron] IV* | [8] |
| IG1502 | *rde-1(ne219) V; Is[wrt-2p::RDE-1_3'unc-54, myo-2p::RFP3]; frIs7[nlp-29p::GFP, col-12p::DsRed IV]* | [9] |
| LD1499 | *atf-4p(uORF)::GFP::unc-54_3'UTR* | [10] |
| FL378 | *erm-1(bab59[ERM-1::mNG::SEC::3xFLAG]) I* | [11] |
| ML2113 | *mcIs67[dpy-7p::Lifeact::GFP; unc-119(+)] V; stIs10088[hlh-1::HIS-24::mCherry, unc-119(+)]* | [12] |
| CZ9334 | *juEx1919[dpy-7p::GFP::RAB-5, ttx-3p::RFP]* | [13] |
| IG1880 | *wrdSi23[eft-3p::TIR::P2A::BFP-NLS-degron:_3'tbb-2] I; frEx614[pZX19(col-19p::DcEntC::FLAG::Degron::mKate_3'unc-54), unc-122p:GFP, rps-0p::HygR]; oxSi1091[mex-5p::Cas9(smu-2 introns) unc-119+] II; unc-119(ed3) III* | This study |
| IG1925 | *wrdSi23[eft-3p::TIR::P2A::BFP-NLS-degron:_3'tbb-2] I; frEx619[pZX25(col-19p::DcEntB::FLAG::Degron::mKate_3'unc-54), unc-122p:GFP, rps-0p::HygR]; oxSi1091[mex-5p::Cas9(smu-2 introns) unc-119+] II; unc-119(ed3) III* | This study |
| IG1926 | *wrdSi23[eft-3p::TIR::P2A::BFP-NLS-degron:_3'tbb-2] I; frEx620[pZX26(col-19p::DcEntA::FLAG::Degron::mKate_3'unc-54), unc-122p:GFP, rps-0p::HygR]; oxSi1091[mex-5p::Cas9(smu-2 introns) unc-119+] II; unc-119(ed3) III* | This study |
| IG1883 | *frEx614[pZX19(col-19p::DcEntC::FLAG::degron::mKate_3'unc-54), unc-122p:GFP, rps-0p::HygR]; frIs7[nlp-29p::GFP, col-12p::DsRed] IV* | This study |
| IG1941 | *frEx619[pZX25(col-19p::DcEntB::FLAG::degron::mKate_3'unc-54), unc-122p:GFP, rps-0p::HygR]; frIs7[nlp-29p::GFP, col-12p::DsRed] IV* | This study |
| IG1942 | *frEx620[pZX26(col-19p::DcEntA::FLAG::degron::mKate_3'unc-54), unc-122p:GFP, rps-0p::HygR]; frIs7[nlp-29p::GFP, col-12p::DsRed] IV* | This study |
| IG1948 | *frEx620[pZX26(col-19p::DcEntA::FLAG::degron::mKate_3'unc-54), unc-122p:GFP, rps-0p::HygR]; frIs7[nlp-29p::GFP, col-12p::DsRed] IV; frIs30[(col-19p::GPA-12gf), pNP21(unc-53pB::GFP)] I* | This study |
| IG1963 | *frEx620[pZX26(col-19p::DcEntA::FLAG::degron::mKate_3'unc-54), unc-122p:GFP, rps-0p::HygR];pmk-1(miy[PMK-1(D327E)]; frIs7[nlp-29p::GFP, col-12p::DsRed] IV* | This study |
| IG1971 | *qxSi727[sta-2p::sfGFP::STA-2; ttTi5605] II; frEx620[pZX26(col-19p::DcEntA::FLAG::degron::mKate_3'unc-54), unc-122p:GFP, rps-0p::HygR]; wrdSi23[eft-3p::TIR::P2A::BFP-NLS-degron:_3'tbb-2] I* | This study |
| IG1977 | *qxSi727[sta-2p::sfGFP::STA-2; ttTi5605] II; frEx619[pZX25(col-19p::DcEntB::FLAG::degron::mKate_3'unc-54), unc-122p:GFP, rps-0p::HygR]; wrdSi23[eft-3p::TIR::P2A::BFP-NLS-degron:_3'tbb-2] I* | This study |
| IG1984 | *wrdSi23[eft-3p::TIR::P2A::BFP-NLS-degron:_3'tbb-2] I; frEx619[pZX25(col-19p::DcEntB::FLAG::degron::mKate_3'unc-54), unc-122p:GFP, rps-0p::HygR]; cguIs1[fib-1p::FIB-1::GFP::fib-1_3'UTR]* | This study |
| IG1596 | *wrdSi23[eft-3p::TIR::P2A::BFP-NLS-degron:_3'tbb-2] I; cguIs1[fib-1p::FIB-1::GFP::fib-1_3'UTR]* | This study |
| IG1998 | *frEx620[pZX26(col-19p::DcEntA::FLAG::degron::mKate_3'unc-54), unc-122p:GFP, rps-0p::HygR]; frIs43[col-12p::SNF-12::GFP, ttx-3p::DsRed2]* | This study |
| IG2022 | *frEx620[pZX26(col-19p::DcEntA::FLAG::degron::mKate_3'unc-54), unc-122p:GFP, rps-0p::HygR]; juEx1919[dpy-7p::GFP::RAB-5, ttx-3p::RFP]* | This study |
| IG2024 | *frEx620[pZX26(col-19p::DcEntA::FLAG::degron::mKate_3'unc-54), unc-122p:GFP, rps-0p::HygR]; mcIs67[dpy-7p::Lifeact::GFP; unc-119(+)] V; stIs10088[hlh-1::HIS-24::mCherry, unc-119(+)]/+* | This study |
| IG2043 | *frEx620[pZX26(col-19p::DcEntA::FLAG::degron::mKate_3'unc-54), unc-122p:GFP, rps-0p::HygR];*  *frIs30[(col-19p::GPA-12gf), pNP21(unc-53pB::GFP)] I* | This study |
| IG2044 | *frEx620[pZX26(col-19p::DcEntA::FLAG::degron::mKate_3'unc-54), unc-122p:GFP, rps-0p::HygR];*  *atf-4(uORF)p::GFP::unc-54_3'UTR* | This study |
| IG2051 | *frEx620[pZX26(col-19p::DcEntA::FLAG::degron::mKate_3'unc-54), unc-122p:GFP, rps-0p::HygR];*  *erm-1(bab59[ERM-1::mNG::SEC::3xFLAG]) I* | This study |

1. Pujol N, Cypowyj S, Ziegler K, Millet A, Astrain A, Goncharov A, et al. Distinct innate immune responses to infection and wounding in the *C. elegans* epidermis. Curr Biol. 2008;18(7):481-9. Epub 2008/04/09. doi: 10.1016/j.cub.2008.02.079. PubMed PMID: 18394898.

2. Labed SA, Omi S, Gut M, Ewbank JJ, Pujol N. The pseudokinase NIPI-4 is a novel regulator of antimicrobial peptide gene expression. PLoS One. 2012;7(3):e33887. Epub 2012/04/04. doi: 10.1371/journal.pone.0033887. PubMed PMID: 22470487; PubMed Central PMCID: PMC3309975.

3. Ashley GE, Duong T, Levenson MT, Martinez MAQ, Johnson LC, Hibshman JD, et al. An expanded auxin-inducible degron toolkit for *Caenorhabditis elegans*. Genetics. 2021;217(3). Epub 2021/03/08. doi: 10.1093/genetics/iyab006. PubMed PMID: 33677541.

4. Dierking K, Polanowska J, Omi S, Engelmann I, Gut M, Lembo F, et al. Unusual regulation of a STAT protein by an SLC6 family transporter in *C. elegans* epidermal innate immunity. Cell Host Microbe. 2011;9(5):425-35. Epub 2011/05/18. doi: 10.1016/j.chom.2011.04.011. PubMed PMID: 21575913.

5. Miao R, Li M, Zhang Q, Yang C, Wang X. An ECM-to-Nucleus Signaling Pathway Activates Lysosomes for *C. elegans* Larval Development. Dev Cell. 2020;52(1):21-37 e5. Epub 2019/11/19. doi: 10.1016/j.devcel.2019.10.020. PubMed PMID: 31735670.

6. Weaver BP, Weaver YM, Omi S, Yuan W, Ewbank JJ, Han M. Non-Canonical Caspase Activity Antagonizes p38 MAPK Stress-Priming Function to Support Development. Dev Cell. 2020;53(3):358-69.e6. doi: 10.1016/j.devcel.2020.03.015.

7. Yi YH, Ma TH, Lee LW, Chiou PT, Chen PH, Lee CM, et al. A Genetic Cascade of *let-7-ncl-1-fib-1* Modulates Nucleolar Size and rRNA Pool in *Caenorhabditis elegans*. PLoS Genet. 2015;11(10):e1005580. Epub 2015/10/23. doi: 10.1371/journal.pgen.1005580. PubMed PMID: 26492166; PubMed Central PMCID: PMCPMC4619655.

8. Kasimatis KR, Moerdyk-Schauwecker MJ, Phillips PC. Auxin-Mediated Sterility Induction System for Longevity and Mating Studies in *Caenorhabditis elegans*. G3 (Bethesda). 2018;8(8):2655-62. doi: 10.1534/g3.118.200278. PubMed PMID: 29880556; PubMed Central PMCID: PMCPMC6071612.

9. Zugasti O, Bose N, Squiban B, Belougne J, Kurz CL, Schroeder FC, et al. Activation of a G protein-coupled receptor by its endogenous ligand triggers the innate immune response of *Caenorhabditis elegans*. Nat Immunol. 2014;15(9):833-8. Epub 2014/08/05. doi: 10.1038/ni.2957. PubMed PMID: 25086774; PubMed Central PMCID: PMC4139443.

10. Statzer C, Venz R, Bland M, Robida-Stubbs S, Meng J, Patel K, et al. ATF-4 and hydrogen sulfide signalling mediate longevity from inhibition of translation or mTORC1. bioRxiv. 2020:2020.11.02.364703. doi: 10.1101/2020.11.02.364703.

11. Bidaud-Meynard A, Nicolle O, Heck M, Le Cunff Y, Michaux G. A V0-ATPase-dependent apical trafficking pathway maintains the polarity of the intestinal absorptive membrane. Development. 2019;146(11). Epub 2019/05/22. doi: 10.1242/dev.174508. PubMed PMID: 31110027; PubMed Central PMCID: PMCPMC7376742.

12. Lardennois A, Pasti G, Ferraro T, Llense F, Mahou P, Pontabry J, et al. An actin-based viscoplastic lock ensures progressive body-axis elongation. Nature. 2019;573(7773):266-70. Epub 2019/08/30. doi: 10.1038/s41586-019-1509-4. PubMed PMID: 31462781.

13. Chuang M, Hsiao TI, Tong A, Xu S, Chisholm AD. DAPK interacts with Patronin and the microtubule cytoskeleton in epidermal development and wound repair. Elife. 2016;5. doi: 10.7554/eLife.15833. PubMed PMID: 27661253; PubMed Central PMCID: PMCPMC5053806.
