# Supplementary material for: Antagonistic fungal enterotoxins intersect at multiple levels with host innate immune defences
Source: PLoS Genet. 2021 Jun 24;17(6):e1009600. doi: 10.1371/journal.pgen.1009600 (PMC8263066; doi:10.1371/journal.pgen.1009600)
Supplement: S2 Table — (DOCX) [file pgen.1009600.s013.docx]

| **Table S2. Oligonucleotide primers** | | |
| --- | --- | --- |
| **Fusion PCR primers** | | |
|  | | |
| **JEP** | **Sequence name** | **Sequence** |
| 2247 | *pDest_pcol-19 R* | GTTGATGAACTGATGTCTTTC |
| 2925 | *3’unc-54* | CATCTCGCGCCCGTGCCTCTGACTTC |
| 3232 | *g4535-F* | GAAAGACATCAGTTCATCAACGCGGCCGCATGTGTTCCGGAGGCTCCAACG |
| 3233 | *g4535-R* | TCGTCATCCTTGTAATCATCGATGTAGTCAGGGGATGTTGCTTTGG |
| 3234 | *FLAG+MKATE-F* | ATCGATGATTACAAGGATGACG |
| 3235 | *FLAG+MKATE-R* | GAGGCACGGGCGCGAGATGTTAACGGTGTCCGAGCTTGGATGGGAGG |
| 3236 | *prps-0:HygR-F* | CACTATAGGGCGAATTGGGTACCATTTTTGCTTTCGTCGTAAATC |
| 3237 | *prps-0:HygR-R* | ATCTGATGACAGCGGCCGCGGCTATTCCTTTGCCCTCGGACG |
| 3247 | *degron-F* | AATCCGGCGCGCCAAGCGCTGCTGGTTCCATGCCTAAAGATCCAGCCAAACC |
| 3248 | *degron-R* | GGACATGGAACCAGCAGCGCTTGGCGCGCCCTTCACGAACGCCGCCGCC |
| 3251 | *g6833-F* | GAAAGACATCAGTTCATCAACGCGGCCGCATGGTTCCGCCACCAGCCTCGCCG |
| 3252 | *g6833-R* | TCGTCATCCTTGTAATCATCGATACCGCTTGTGTGCCCCTTCTTTGC |
| 3253 | *g2698-F* | GAAAGACATCAGTTCATCAACGCGGCCGCATGTTCATTGGCTTTACTGTCACC |
| 3254 | *g2698-R* | TCGTCATCCTTGTAATCATCGATAGGATGCAACTCGCATTTTCC |
| 3269 | *g2819-F* | GAAAGACATCAGTTCATCAACGCGGCCGCATGCACTTGGTCCCATCTGACG |
| 3270 | *g2819-R* | TCGTCATCCTTGTAATCATCGATGGCCTTTTCGCCGCCAAAACCTTTGC |
| 3271 | *g7949-F* | GAAAGACATCAGTTCATCAACGCGGCCGCATGCGCCGGCACAAGCTCAAGCCCG |
| 3272 | *g7949-R* | TCGTCATCCTTGTAATCATCGATTGAGTCTTTGATTTCCGAGAAGCC |
|  |  |  |
|  |  |  |
| **qRT-PCR primers** | | |
|  | | |
| **JEP** | **Sequence name** | **Sequence** |
| 538 | *act-1* | CCATCATGAAGTGCGACATTG |
| 539 | *act-1* | CATGGTTGATGGGGCAAGAG |
| 952 | *nlp-29* | TATGGAAGAGGATATGGAGGATATG |
| 848 | *nlp-29* | TCCATGTATTTACTTTCCCCATCC |
| 950 | *nlp-31* | GGTGGATATGGAAGAGGTTATGGAG |
| 953 | *nlp-31* | GTCTATGCTTTTACTTTCCCC |
| 969 | *nlp-34* | ATATGGATACCGCCCGTACG |
| 970 | *nlp-34* | CTATTTTCCCCATCCGTATCC |
| 549 | *cnc-2* | TCCCATGCCCATACCGTAAC |
| 944 | *cnc-2* | CCGCTCAATATGGTTATGGAG |
| 1124 | *cnc-4* | ACAATGGGGCTACGGTCCATAT |
| 1125 | *cnc-4* | ACTTTCCAATGAGCATTCCGAGGA |
| 1676 | *irg-1* | CCATGGAATGAAACTTGTGG |
| 1677 | *irg-1* | CCAGTTTCGTTCATCTTCACA |
| 2340 | *ifas-1* | TTCCTGAGTGCTCACGAAGG |
| 2341 | *ifas-1* | AACACTGAGGAACGACCAGG |
| 2863 | *hsp-4* | GCCATCTCGTGGAATCAACC |
| 2864 | *hsp-4* | GTGAGTGGATTGACGTCAAG |
| 2876 | *hsp-6* | CAACAGATCGTTATCCAATC |
| 2877 | *hsp-6* | TTCTCTTTGCGTCCTCAGC |
| 3119 | *hsp-60* | GTTGAAGTTGGAGAGAAGAAGGACC |
| 3120 | *hsp-60* | ATCTGAGAAGAGCAACACCTCC |
| 3307 | *gst-4* | GAAAATTTGGACTCGCTGG |
| 3308 | *gst-4* | AAGAAATCATCACGGGCTGG |
| 1795 | *gpdh-1* | AGCACTAAAGAACATTGTCGCC |
| 1796 | *gpdh-1* | TGGTAATGAGATCAGCCACTCC |
| 3179 | *mKate2* | ACAACGTCAAGATCCGTGGAGTC |
| 3180 | *mKate2* | GGTAGGTGGTCTTGAGGTTGC |
