# Supplementary material for: Antagonistic fungal enterotoxins intersect at multiple levels with host innate immune defences
Source: PLoS Genet. 2021 Jun 24;17(6):e1009600. doi: 10.1371/journal.pgen.1009600 (PMC8263066; doi:10.1371/journal.pgen.1009600)
Supplement: S3 Table — (DOCX) [file pgen.1009600.s014.docx]

| **Phenotype** | **DcEntA** | **DcEntB** | **DcEntC** |
| --- | --- | --- | --- |
| Low or irregular pumping rate | **x** | **x** | **x** |
| Abnormal locomotion or lethargic | **x** |  | **x** |
| Protruding vulva | **x** | **x** |  |
| Bursting (through vulva) |  | **x** |  |
| Single gonad arm | **x** | **x** |  |
| Asymmetric gonad arms | **x** |  | **x** |
| Egg retention |  |  | **x** |
| Major morphological defects (e.g. square head) | **x** |  |  |

**Supplementary Table 3. Phenotypes of transgenic worms expressing one of the 3 candidate virulence factors.** Ten worms of each strain cultured at 25˚C were inspected as 2-day old adults. A cross indicates that at least one worm exhibited the phenotype.
