# Supplementary material for: Antagonistic fungal enterotoxins intersect at multiple levels with host innate immune defences
Source: PLoS Genet. 2021 Jun 24;17(6):e1009600. doi: 10.1371/journal.pgen.1009600 (PMC8263066; doi:10.1371/journal.pgen.1009600)
Supplement: Attachment — Submitted filename: Zhang_response.pdf [file pgen.1009600.s017.pdf]

We thank the reviewers for their extensive and constructive remarks. We were pleased that they found the work to be novel and highly significant. Below, we address their major/specific comments. If needed, we could equally detail how we propose to address all of the numerous minor comments by specific clarifications in the text or small changes to the figures.

Reviewer 1.

The correlation with translation inhibition upon fungal infection and its interaction with the p38 MAPK pathway is not very well articulated. Since this does not add significantly to the conclusions of the study, the authors could consider removing this part or provide more evidence to support it.

Our investigation with these fungal virulence factors revealed a new aspect of the epidermal innate immune response that we had previously overlooked. As such, we propose to follow the reviewer's suggestion and would provide more evidence to support it. Specifically, we have now conducted experiments with a known reporter of translation inhibition (*[Patf-4(uORF)::GFP::unc-54(3'UTR)];* Venz *et al.,* G3*,* 2020) that confirm the preliminary data presented in the original manuscript and would include this in a revised version.

If host ARF proteins are required by DcEntA to cause ADP ribosylation of targets, is the prediction that arf mutants would be less susceptible to D. coniospora infection? Or, would arf knockdown by RNAi be sufficient to reduce the negative effect of DcEntA on nlp-29 gene induction? There may be technical limitations in performing these experiments but at least some discussion on hypotheses arising from the presented model would be helpful to include in the manuscript.

We did conduct RNAi experiments knocking down the expression of ARF-1.1 and ARF-1.2, which we identified as DcEntA interactors. Alone, *arf-1.1*(*RNAi*) had no obvious effect, but as *arf-1.2*(*RNAi*) alone, or in combination with *arf-1.1*(*RNAi*) renders worms sick, we have not been able to pursue the question. As suggested by the reviewer, we would therefore add some discussion on these hypotheses in a revised version.

Have the authors attempted host-induced gene silencing of DcEntA and DcEntB to study their putative significance during fungal infection? Is this feasible to do in order to prioritise which fungal virulence factors to study in the future?

The reviewer is alluding to reports that for several phytopathogenic fungi, host-derived dsRNA or ssRNA can enter an invading pathogen and through the fungal RNAi machinery silence specific genes (e.g. PMID 20884801). As described in a publicly available PhD thesis (http://www.theses.fr/2016AIXM4080/document), we made various attempts to apply this technique during infection of *C. elegans* by *D. coniospora*. Despite evidence for the existence of a functional RNAi pathway in *D. coniospora*, none of these approaches were successful. If deemed appropriate, we could certainly mention this briefly in a revised version.

Reviewer 2.

Would it be possible to modulate the level of expression to observe the effects of lower doses of the virulence factor by adjusting the concentration of auxin? A dose-response fitness curve could be established or irg-1 induction monitored (it is induced by DcEntA but not by Drechmeria infection).

The control experiments suggested by the reviewer are certainly feasible and we could conduct them if deemed absolutely necessary, but we believe that the comment can be answered in 2 other ways. The first is that the appearance of all of the phenotypes that we have observed coincides with the established temporal expression profile for *col-19* promoter activity. We omitted to highlight this fact, but would do so in a revised version. Secondly, we have previously shown that GPA-12* represses *col-19* expression (Lee et al. 2018). We now have data for worms expressing DcEntA in a GPA-12* background. We observe a reduction of DcEntA expression, a suppression of the usual sickness and an abrogation of *F40H7.12* expression. We propose to include these results that indicate that the phenotypes we observe are due to DcEntA expression in a revised version.

a second category of control would be to express mutant virulence factors in which the ADP-ribosylating activity is impaired by point mutation.

We could make such mutants if deemed absolutely essential; their production and subsequent assay would take several months. On the other hand, the ADP-ribosylating activity of enteroxins is well established, so that researchers often do not consider it necessary to provide such a control. For example, in their 2001 *C. elegans* study, Darby and Falkow did not make ADP-ribosylating dead mutant. We would rather propose modulating the text to explain that the *D. coniospora* enterotoxins have all the sequence features exhibited by other validated ADP-ribosylating enterotoxins and are presumed therefore to be enzymatically active.

It is not clear to this reviewer whether there is a Drechmeria infection-induced nuclear uptake of this transcription factor, as it is apparently already found in noninfected nematodes when using a transgenic reporter (Dierking et al., 2011) while it appears to be absent upon STA-2 antibody staining (Zhang, et al., Immunity, 2015). Is it possible to quantify the nuclear concentration of this transcription factor using the STA-2 specific antibody prior to and during Drechmeria infection? It would also be needed to monitor this parameter upon DcEntA expression to confirm the results obtained with the transgenic fusion protein.

The reviewer is correct that in our previous work, using a strain carrying a multi-copy transgene with GFP::STA-2 expressed under control of a strong epidermal promoter (col-12p::GFP::STA-2), GFP::STA-2 was seen in the nucleus. The reviewer is also correct that Zhang, et al. reported results regarding STA-2 localisation using an anti-STA-2 antibody. Notably, there was no quantitative demonstration of the expected increase in nuclear STA-2 upon hemidesmosome disassembly. We note also that we have also failed to replicate a number of their findings (see pp52-57 in http://www.theses.fr/2017AIXM0513/document). In

2015, we did request an aliquot of the antibody, but there were apparently practical barriers, so we have never been able to replicate or extend the Zhang, et al. study. On the other hand, we do now have two transgenic strains that are expected to express a STA-2 reporter protein at the physiological level, as they are single-copy knock-ins, (*frSi11*[pNP152(*sta-2p::STA-2::mKate*), *unc-119(+) ttTi5605*]) and *qxIs727* (*sta-2p::GFP::STA-2*), kindly provided by Xiaochen Wang (Miao *et al*., Dev Cell, 2020). They behave identically in all assays that we have conducted, and notably, both show that some STA-2 is present constitutively in the nucleus. We reported results with the latter strain in the MS. To address the reviewer's comment, we propose to undertake a quantification of STA-2 nuclear occupancy following *D. coniospora* using *qxIs727* and to report the results we obtain in a revised version.

if the two factors were expressed at levels similar to those happening during infection, would this effect still be apparent? In the present setting, is it possible, at least, to overexpress jointly both virulence factors? What would happen to STA-2 subcellular localization?

Unfortunately, we do not know their expression level during infection as we cannot tag the endogenous protein and do not have the necessary specific antibodies. Regardless of this, it seems unlikely that of the hundreds of secreted potential virulence factors, DcEntA and DcEntB are the only fungal proteins that can affect STA-2 localisation. Thus such a test, expressing DcEntA and DcEntB together, while answering the reviewer's question, does not seem to us to be a priority. Thus, although we could perform the experiment if deemed essential, but propose rather to add a sentence addressing the issue in a revised version.

The link made between DcEntB and translation inhibition at the end of the paragraph on cycloheximide (CHX) treatment appears to be tenuous. The fact that CHX affects nucleolus morphology, as DcEntB does, does not imply that DcEntB also inhibits translation, in as much as F40H7.12 is also not induced by DcEntB.

We showed that DcEntB suppresses F40H7.12 expression in a *sta-2*-dependent manner. We also showed that DcEntB increases the nuclear occupancy of STA-2. The simplest explanation for these observations is that nuclear STA-2 suppresses F40H7.12 expression. We would clarify this in the text. Further, to address the reviewer's point directly, we would use the *[Patf-4(uORF)::GFP::unc-54(3'UTR)]* reporter referred to above, and report our observations in a revised version.

Reviewer 3.

The protein interaction studies are over-interpreted given the data presented. What is the negative control in these binding studies? Does the negative control contain the other domains added to these overexpression constructs? None of the interactions identified using IP-MS were validated using a second method nor was the function of these interactions tested. Thus, while providing potentially interesting datasets, these data should be treated as much more preliminary than presented. The description of the pathway analyses of these candidate interactors should therefore be toned down.

These remarks can be contrasted with those of reviewer 1: *Another strength of the study is the biochemical Co-IP/MS experiments to identify potential toxin interactors and discussion around this topic. This will provide a great resource to dissect in the future the exact effects of these toxins on host cell physiology*.
Regarding the controls, we regret that we were not clear enough in the text. As we wrote in the Methods, "This dataset also includes unpublished pull-down data for three other structurally-unrelated virulence factors (Harding et al., manuscript in preparation) used to identify proteins that bound in a non-specific manner… Proteins that passed the combined threshold in a second strain (PRIDE repository PXD021929) were considered as potential cross-reactive, non-specific binders and were filtered out from the volcano plot and summary spreadsheets". In other words, we have data for 5 different fungal proteins, each with the same tags. For each, we reported only those host proteins that were found to be enriched in a statistically significant manner uniquely in the sample in question. We would make this clearer in a revised version.
To respond to the reviewer's other comments, on the basis of the biochemical data, we have continued our investigation of the potential mode of action of the virulence factors using newly acquired strains expressing chimeric reporter proteins. Thus, we now know that DcEntA is clearly associated with endosomes, and also with the ezrin/moesin/radixin protein ERM-1, and with actin filaments.

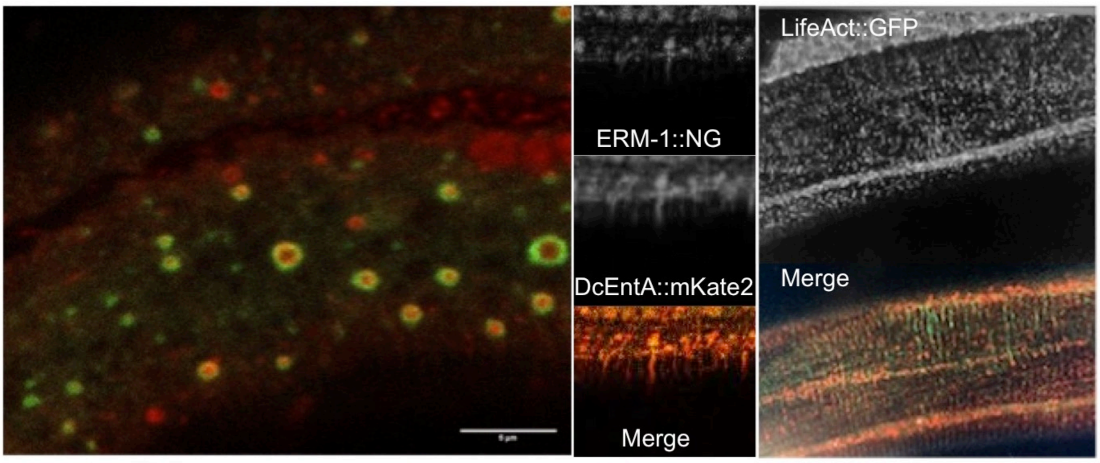

DcEntA (red) colocalises with endosomal vesicles (left panel, RAB-5::GFP and DcEntA::mKate2), with ERM-1 (middle panels: top, ERM-1::NG; middle, DcEntA::mKate2; bottom, merge) and with actin filaments (right panel, top, LifeAct::GFP; bottom, merge).

Consequently, rather than "toning down" the analyses, propose to reinforce them with this data. Thus, we would propose to modify the relevant part of the results with a text such as, "*Several other components of C. elegans intracellular vesicle transport machinery were identified, including 16 that have been reported to interact physically with VPS-45, orthologue of human VPS45 (vacuolar protein sorting 45 homolog), required for RAB-5-dependent endocytic transport (Gengyo-Ando et al., 2007), and 11 interactors of LET-413 (Waaijers et al., 2016), the nematode Erbin protein that acts as a RAB-5 effector during endocytic recycling (Liu et al., 2018)* ***and that physically interacts with RAB-5 (Liu et al., 2017). Consistent with these observations, we observed the presence of DcEntA in RAB-5-associated vesicles [new data as panel in Figure 4]. Notably, the***

normal pattern of RAB-5 expression was disrupted in the strain expressing DcEntA [new data as panel in Figure 4]. *The list of candidate DcEntA interactors also included several proteins involved in cytoskeleton dynamics, including the nematode ezrin/moesin/radixin orthologue ERM-1, the microtubule plus-end binding protein EBP-1, and the twinfilin homologue TWF-2, an actin binding protein.* ***In agreement with the biochemical data, DcEntA clearly colocalized with ERM-1::NG and with the F-actin marker LifeAct::GFP [new data as panel in Figure 4]. Expression of DcEntA was also found to disrupt the normal pattern of both ERM-1::NG and LifeAct::GFP in hyp7 [new data as panel in Figure 4].*** *Coordinated changes in microtubule and actin dynamics are required for the proper recruitment of SNF-12 to sites of injury in hyp7. Without SNF-12 recruitment, there is an abrogation of the immune response (Taffoni et al., 2020). There are thus several potential ways that DcEntA could influence membrane trafficking and SNF-12 localisation*". We believe that this would substantially strengthen this part of our study in a revised version.

Along this same line, the authors do not show that "DcEntA has ADP-ribosylation activity" as they say in the title of that Results section (hey demonstrate an interaction with an ADP-ribosylase, but don't confirm this interaction or show that it has any functional significance). Similarly, the identification of an ADP-ribosylation modification on DcEntA doesn't demonstration auto-ADP-ribosylation.

As acknowledged above, the reviewer is correct that we do not present any formal proof that DcEntA and DcEntB, proteins that have all the sequence features found in validated ADP-ribosylases, do have the predicted enzymatic activity. We would therefore modulate the text in a revised version.

There are some issues with the presentation of several sets of data. N and P values are not provided in Fig. 5A and B, Fig. S3, and Fig. S4. P values are not provided in Fig. 3 and Fig 10F. It would be helpful to quantify the frequency of at least some of the phenotypes presented (ie Fig. 3D and F, Fig. 4A and B, Fig. 8A and B, Fig. 9A and B, Table S4). Many studies provide an n of only 2; while these data generally look very solid, adding a third repeat would be better.

We would propose to modify the text as many of the details are in the figure legends already. We would add p values wherever necessary. There are figures where we deliberately omitted them, as the results were patently obvious. The same applies for quantification. For example, we did not provide any for Fig. 4A and B, but here and elsewhere, could do so if judged necessary. For Fig 5A and B, the N values are in the legend. In cases where there is any ambiguity, a third repeat could be included in a revised version.

There seems to be lots of worm genes and worm jargon presented to the reader, and I wonder how well the non-expert will follow everything. Could schematics be added to some figures better outlining the signaling pathways/target genes being tested when the specific data are presented. Similarly, the authors don't really explain (at least not in sufficient detail) what nipi, nlp, cnc genes are. Likewise, wherever possible, I would use clearer transgene nomenclature - ie

refer in the text and legends to nlp-29::gfp, not frIs7, so the reader doesn't have to look up the strains while reading the results.

We would modify the text and add the suggested schematics in a revised version.

While DcEntC expression did not affect lifespan, it would be interesting to test if DcENtC does in fact have an effect in the presence of fungus; the authors could be missing something interesting here.

Although the reviewer raises an interesting point, we think that adding the results of such an analysis of DcEntC would add unwarranted complexity to the current study. We propose, however, to mention such an idea in a revised version.

Our study aimed at exploring the potential mechanisms involving DcEntA and DcEntB, and we firmly believe that this is, of itself, of interest. Indeed, the significance of the work as such was recognised by the 3 expert reviewers. On the other hand, after reading the preprint, certain colleagues have raised the question of the actual role of enterotoxins during natural infection. As we already state, technical barriers preclude the direct detection of DcEntA or DcEntB during infection or the assay of the consequences of their knock-down (see also the response regarding host-induced gene silencing, above). We would, however, add a new Supplementary Table summarising all the available transcriptome data for all *D. coniospora* enterotoxin genes, complementing our own data with that from Zhang *et al*. (PMID: 26975455). These data clearly support the supposition that enterotoxins are expressed during natural infection of *C. elegans*. Given the high-confidence prediction of signal peptides, and the unusual expansion of the family in *D. coniospora*, there is every reason to believe that the enterotoxins do play an important role during infection and these arguments will be made more clear in the Discussion.
Colleagues have also asked how our findings compare to the role of enterotoxins in other infections. In the current MS, we refer to a broad review on enterotoxins (Krueger and Barbieri) and specific examples from *C. elegans*. If judged absolutely necessary, we could extend this part of the discussion to a broader review of enterotoxin action.
