# Supplementary material for: Antagonistic fungal enterotoxins intersect at multiple levels with host innate immune defences
Source: PLoS Genet. 2021 Jun 24;17(6):e1009600. doi: 10.1371/journal.pgen.1009600 (PMC8263066; doi:10.1371/journal.pgen.1009600)
Supplement: Attachment — Submitted filename: Zhang_Response.docx [file pgen.1009600.s018.docx]

**Reviewer 1.**

**Major comments:**

The correlation with translation inhibition upon fungal infection and its interaction with the p38 MAPK pathway is not very well articulated. Since this does not add significantly to the conclusions of the study, the authors could consider removing this part or provide more evidence to support it.

Our investigation with these fungal virulence factors revealed a new aspect of the epidermal innate immune response that we had previously overlooked. As suggested by the reviewer, we therefore now provide more evidence to support it. Specifically, we have conducted experiments with a known reporter of translation inhibition (*[atf-4p(uORF)::GFP];* Venz *et al.,* G3*,* 2020) that confirm the preliminary data presented in the original manuscript and include this (as the new Figure 5G&H, 9G and S7).

If host ARF proteins are required by DcEntA to cause ADP ribosylation of targets, is the prediction that arf mutants would be less susceptible to D. coniospora infection? Or, would arf knockdown by RNAi be sufficient to reduce the negative effect of DcEntA on nlp-29 gene induction? There may be technical limitations in performing these experiments but at least some discussion on hypotheses arising from the presented model would be helpful to include in the manuscript.

We did conduct RNAi experiments knocking down the expression of ARF-1.1 and ARF-1.2, which we identified as DcEntA interactors. Alone, *arf-1.1*(*RNAi*) had no obvious effect, but as *arf-1.2*(*RNAi*) alone, or in combination with *arf-1.1*(*RNAi*) renders worms sick, we have not been able to pursue the question. As suggested by the reviewer, we have added this information to the text.

Have the authors attempted host-induced gene silencing of DcEntA and DcEntB to study their putative significance during fungal infection? Is this feasible to do in order to prioritise which fungal virulence factors to study in the future?

The reviewer is alluding to reports that for several phytopathogenic fungi, host-derived dsRNA or ssRNA can enter an invading pathogen and through the fungal RNAi machinery silence specific genes (e.g. PMID 20884801). As described in a publicly available PhD thesis (http://www.theses.fr/2016AIXM4080/document), we made various attempts to apply this technique during infection of *C. elegans* by *D. coniospora*. Despite evidence for the existence of a functional RNAi pathway in *D. coniospora*, none of these approaches were successful. As this was not deemed essential by the editor, we have not mentioned this.

**Minor comments:**

page 12: "This presumably reflected stochastic loss of protein expression in the epidermis"

This sentence may be unclear to readers who are not familiar with C. elegans transgenesis

In the light of comments from Reviewer #3, who judged this first part of the Results “distracting”, we have removed the corresponding section, so this is no longer relevant.

page 12. "large family of enterotoxin a domain proteins in Drechmeria"

Add reference or explain what you mean by large family

We have altered this section and added 2 new supplementary figures (Figure S2 and S3) to address this point, as well as the point about Supplementary Fig S3 (below) and Reviewer #2’s minor point “a”.

page 13. "Enterotoxin expression in the epidermis makes worms more susceptible to infection". This is a very interesting result. Can the authors comment perhaps in the discussion whether the enterotoxins are likely to reach the epidermal syncytium during fungal infection or do the authors think that they may act in multiple tissues? Do the authors think these toxins enter cells or could they also act in the body cavity? It would be interesting to try heterologous expression in the future without removing the signal peptide as another control or in different tissues.

The reviewer makes a number of interesting points. We have added a discussion on where the enterotoxins are likely to act during an infection.

page 13. "albeit at an elevated level (Sup Fig S3)"

This is unclear or can be misinterpreted e.g. by assuming that the reference is the expression of these toxins during infection. Figure S3 shows that DcEntC is more highly expressed than DcEntA/B in the recovered transgenics, which is interesting since DcEntC ox animals show subtle phenotypes despite having higher level of expression of the corresponding transgene.

Please see above.

page 13: "DcEntA blocks AMP expression"

Maybe add a comment at this point on the expression of the Red marker of frIs7 - I assume it is not changed but good to argue against a general block in transcription.

page 14: "This indicates that DcEntA acts downstream of gpa-12 to block defence gene expression"

It is not possible to rule out that DcEnt A is acting independently (rather than downstream) so I suggest modifying to "downstream or in parallel". Same for the PMK-1 activation experiment described in the next paragraph on the same page.

We have made the suggested edits.

page 14: "progressive disruption of vesicular pattern of SNF-12 at the hypodermis".

I would suggest removing the word progressive as only one snapshot is provided. This looks like a very substantial change in expression. Can you describe the new pattern in more detail in the text and figure legend (I think this only comes much later on page 18) ? Do you now see ectopic expression in the seam cells? There seems to be more SNF-12::GFP signal now in the membrane, is that correct ?

To address this comment, we have added several examples of the SNF-12 pattern in worms expressing different levels of DcEntA (revised Figure 4) and describe our observations in the text.

page 15: Is there any evidence that F40H7.12 is part of a "defence" programme? Please add reference if this is already shown. This is important because although nlps are well-studied, the role of F40H7.12 is unclear which creates some confusion about the significance of this result. Also, is there any evidence that F40H7.12 is induced upon DcEntA expression in the epidermis? This needs to be mentioned in the discussion.

In the light of this comment and minor comments from the other 2 reviewers, we have produced a short article about F40H7.12 (now called *ifas-1*), include this with the revised MS and refer to it in the revised MS (as Omi *et al*., submitted). We have also added data and discussion about *ifas-1* regarding its induction as suggested.

page 15: "possibly because of an action blocking translation"

I would remove this claim since not enough data are shown to thoroughly support it.

As mentioned above, we now provide several new lines of evidence to support the inhibition of translation upon infection and enterotoxin expression.

Figure 2 legend: Clarify what "control" means in 2B (is it the same with Fig. 2D) and whether this applies to panel 2C as well?

We have modified the legends to make this clear.

page 16 and Figure 5D: Unclear what is the reference for comparison. I wasn't sure whether the expression levels shown are in comparison to some control RNAi treatment? Please add this information to the figure legend.

page 19: "The latter is a measure of maximum length..." - this definition is not clear for the reader so needs to be further explained.

Figure 9: Sample used as experimental reference here is not clear. If control RNAi bacteria are used, it is best to specify this in the figure legend and perhaps add image in panel B.

In all these cases, we have explained more clearly the controls, definitions and comparisons.

The authors find that irg-1 expression is induced by heterologous expression of DcEntA but irg-1 expression is not induced 12-24 hours post infection by D. coniospora. An explanation or hypothesis as to why that may be the case would be helpful.

We now include an explanation as suggested, as part of the more detailed analysis of translational inhibition (see above).

How do the authors interpret then that DcEntB makes worms more susceptible to fungal infection (panel 2D). An mKate2 only control in the future may help rule out mild effects due to FP expression ?

As we now make clearer, DcEntB expression actually has a protective effect when worms are infected with a very high concentration of spores.

Fig 3: Consider labelling frIs7 to help the reader understand the GFP/dsRED markers present in this transgene.

Fig 4: Scale bar missing in panel A and D.

Fig.7. PMK-1 changes in phosphorylation in the presence of DcEntA are not shown. Role of translation is unclear in terms of targets and significance. The authors could present a combined model for both toxins in a single figure.

Fig 9: Scale bar is displaced in B. Missing * of significance from panel C and D.

Fig 10: Missing * of significance from panel F. Please check all figures.

All these points have been corrected or addressed.

**Reviewer 2.**

Would it be possible to modulate the level of expression to observe the effects of lower doses of the virulence factor by adjusting the concentration of auxin? A dose-response fitness curve could be established or irg-1 induction monitored (it is induced by DcEntA but not by Drechmeria infection).

Although the control experiments suggested by the reviewer are feasible, with editorial agreement, we have addressed these comments in 2 ways. First, we correlate DcEntA expression levels with the severity of the observed phenotypes, for example in Figures 4 and 5. Secondly, we have previously shown that GPA-12* represses *col-19* expression (Lee et al. 2018). We now have data for worms expressing DcEntA in a GPA-12* background. We observe a reduction of DcEntA expression, a suppression of the usual sickness and an abrogation of *F40H7.12/ifas-1* expression (Figure S5). We have included these results that indicate that the phenotypes we observe are due to DcEntA expression in the revised version.

a second category of control would be to express mutant virulence factors in which the ADP-ribosylating activity is impaired by point mutation.

With editorial agreement, we will not be making such mutants, but have rather modulated the text to explain that the *D. coniospora* enterotoxins have all the sequence features exhibited by other validated ADP-ribosylating enterotoxins and are presumed therefore to be enzymatically active.

It is not clear to this reviewer whether there is a Drechmeria infection-induced nuclear uptake of this transcription factor, as it is apparently already found in noninfected nematodes when using a transgenic reporter (Dierking et al., 2011) while it appears to be absent upon STA-2 antibody staining (Zhang, et al., Immunity, 2015). Is it possible to quantify the nuclear concentration of this transcription factor using the STA-2 specific antibody prior to and during Drechmeria infection? It would also be needed to monitor this parameter upon DcEntA expression to confirm the results obtained with the transgenic fusion protein.

The reviewer is correct that in our previous work, using a strain carrying a multi-copy transgene with GFP::STA-2 expressed under control of a strong epidermal promoter (*col-12*p::GFP::STA-2), GFP::STA-2 was seen in the nucleus. The reviewer is also correct that Zhang et al. reported results regarding STA-2 localisation using an anti-STA-2 antibody. Notably, in that paper, there was no quantitative demonstration of the expected increase in nuclear STA-2 upon hemidesmosome disassembly. We note also that we have also failed to replicate a number of their findings (see pp 52-57 in http://www.theses.fr/2017AIXM0513/document). In 2015, we did request an aliquot of the antibody, but there were apparently practical barriers, so we have never been able to replicate or extend the Zhang et al. study. On the other hand, we do now have two transgenic strains that are expected to express a STA-2 reporter protein at the physiological level, as they are single-copy knock-ins, one we generated, (*frSi11*[pNP152(*sta-2p::STA-2::mKate*), *unc-119(+) ttTi5605*]) and *qxIs727* (*sta-2p::GFP::STA-2*), kindly provided by Xiaochen Wang (Miao *et al*., Dev Cell, 2020). They behave identically in all assays that we have conducted, and notably, both show that some STA-2 is present constitutively in the nucleus. To address the reviewer’s comment, we have quantified STA-2 localisation following *D. coniospora* infection using *qxIs727* and now report the observed correlation between AMP gene expression and increased nuclear occupancy (Figure S6).

if the two factors were expressed at levels similar to those happening during infection, would this effect still be apparent? In the present setting, is it possible, at least, to overexpress jointly both virulence factors? What would happen to STA-2 subcellular localization?

Unfortunately, we do not know their expression levels during infection as we cannot tag the endogenous proteins and do not have the necessary specific antibodies. Regardless of this, it seems unlikely that of the hundreds of secreted potential virulence factors, DcEntA and DcEntB are the only fungal proteins that can affect STA-2 localisation. Thus such a test, expressing DcEntA and DcEntB together, while answering the reviewer’s question, does not seem to us to be a priority. With editorial approval, we have rather added text addressing the issue to the discussion of the revised version.

The link made between DcEntB and translation inhibition at the end of the paragraph on cycloheximide (CHX) treatment appears to be tenuous. The fact that CHX affects nucleolus morphology, as DcEntB does, does not imply that DcEntB also inhibits translation, in as much as F40H7.12 is also not induced by DcEntB.

We showed that DcEntB suppresses F40H7.12/*ifas-1* expression in a *sta-2*-dependent manner. We also showed that DcEntB increases the nuclear occupancy of STA-2. As we now make explicit, the simplest explanation for these observations is that nuclear STA-2 suppresses *ifas-1* expression. Further, to address the reviewer’s point directly, we used the *[atf-4p(uORF)::GFP]* reporter referred to above, and report our observation of its induction by DcEntB (Figure 9G). This has allowed us to refine our model (Figure 10) in the revised version.

**Minor comments**

a) The authors refer to "an unusually large number of genes for enterotoxin alpha subunit proteins". Does this mean tens, hundreds of genes? It is also not specified how the three enterotoxins have been chosen from this apparently large pool.

We have altered this section and added 2 supplementary figures (Figure S2, S3) to address these points.

b) Is the depurination caused by RIPs occurring all over the ribosomal RNA or at a specific site in the sarcin-ricin loop?

c) Is F40H7.12 also induced by the expression of the RIP virulence factor?

In the light of comments from Reviewer #3, who judged this first part of the Results “distracting”, we have removed the corresponding section, so these points are no longer relevant.

d) Is any function known for F40H7.12 and could it be related in some ways to some of the proteins that appear to bind to DcEntA?

In the light of this comment and a minor comment of reviewer #1, as detailed above, we have now produced a short article about F40H7.12/*ifas-1* and refer to it in the revised MS.

e) Discussion: the statement that fluorescent reporter proteins are accurate as regards other model organisms likely needs some modulation. While using only a short 3xHA-tag, Bischof et al. (Development, 2013) reported in the Drosophila model that the overexpression of tagged proteins led to a 20% false-negative rate, that is, the tagged proteins were not functional. Thus, one could imagine that their subcellular localization might also be affected.

f) In the Introduction, the authors state that gene knock-down is possible in D. coniospora; yet, in the Discussion, they mention that "mutants are not available". Some clarification is required on this point.

These suggestions have been implemented.

g) Some references appear to be incomplete, e.g., Caragine et al., Eliyahu et al., or Taffoni et al.

We thank the reviewer for pointing out this problem. It was caused by an erroneous field attribution in the corresponding Pubmed records for some journals and has been corrected.

h) Fig. 4: controls with uninfected and D. coniospora-infected wild-type worms would be helpful for both SNF12::GFP and STA-2::GFP.

We now provide a quantification of STA-2::GFP nuclear occupancy as requested (see also above). For SNF-12::GFP, we have previously published a detailed examination of its dynamics following wounding (Taffoni et al., 2020), which we refer to, and are currently preparing a manuscript describing the even more complex changes that accompany infection.

i) Fig 9A: why are worms not dying faster than in Fig. 2D if infected with a 10-fold higher dose?

The control and DcEntA-expressing worms do in fact die more rapidly. We have made this clearer in the revised version.

j) Legend to Fig. 4: ImageJ is mentioned whereas Fiji is referred to in Methods.

This has been corrected.

k) Fig. S2C: a picture at higher resolution is desirable as some aliasing can be observed.

As Fig. S2 has been removed, this is no longer relevant.

Fig. S6 is difficult to understand for a nonspecialist. The captions are not readable on a printed figure, even with a magnifying glass.

The figure has been modified to make it more legible.

**Reviewer 3.**

The protein interaction studies are over-interpreted given the data presented. What is the negative control in these binding studies? Does the negative control contain the other domains added to these overexpression constructs? None of the interactions identified using IP-MS were validated using a second method nor was the function of these interactions tested. Thus, while providing potentially interesting datasets, these data should be treated as much more preliminary than presented. The description of the pathway analyses of these candidate interactors should therefore be toned down.

These remarks can be contrasted with those of reviewer 1: *Another strength of the study is the biochemical Co-IP/MS experiments to identify potential toxin interactors and discussion around this topic. This will provide a great resource to dissect in the future the exact effects of these toxins on host cell physiology*.

Regarding the controls, we regret that we were not clear enough in the text. As we wrote in the Methods, “This dataset also includes unpublished pull-down data for three other structurally-unrelated virulence factors (Harding et al., manuscript in preparation) used to identify proteins that bound in a non-specific manner… Proteins that passed the combined threshold in a second strain (PRIDE repository PXD021929) were considered as potential cross-reactive, non-specific binders and were filtered out from the volcano plot and summary spreadsheets”. In other words, we have data for 5 different fungal proteins, each with the same tags. For the 2 enteroxins analysed here, we reported only those host proteins that were found to be enriched in a statistically significant manner uniquely in the sample in question. Note that we have already made the raw data for all 5 fungal proteins publicly available. We have made this clearer in the revised version.

To respond to the reviewer’s other comments, we have substantially honed the description of pathway analyses, especially for DcEntB. At the same time, on the basis of the biochemical data, we extended our investigation of the potential mode of action of the virulence factors. Thus, we now demonstrate that DcEntA is clearly associated with endosomes, and also with actin filaments, as well as with ERM-1 that was identified as a potential direct interactor. Consequently, we have reinforced this part of the manuscript with additional data (Figure 6B-E), and modified the text accordingly. We believe that this represents an important improvement to our study.

Along this same line, the authors do not show that "DcEntA has ADP-ribosylation activity" as they say in the title of that Results section (hey demonstrate an interaction with an ADP-ribosylase, but don't confirm this interaction or show that it has any functional significance). Similarly, the identification of an ADP-ribosylation modification on DcEntA doesn't demonstration auto-ADP-ribosylation.

As acknowledged above, the reviewer is correct that we do not present any formal proof that DcEntA and DcEntB, proteins that have all the sequence features found in validated ADP-ribosylases, do have the predicted enzymatic activity. We have therefore modulated the text.

There are some issues with the presentation of several sets of data. N and P values are not provided in Fig. 5A and B, Fig. S3, and Fig. S4. P values are not provided in Fig. 3 and Fig 10F. It would be helpful to quantify the frequency of at least some of the phenotypes presented (ie Fig. 3D and F, Fig. 4A and B, Fig. 8A and B, Fig. 9A and B, Table S4). Many studies provide an n of only 2; while these data generally look very solid, adding a third repeat would be better.

We have made all the appropriate changes, following editorial consultation. In a number of cases, we have added extra replicates and/or p values as suggested. In other cases, as the results with reporter genes are so clear cut and have been corroborated by qRT-PCR analyses, we have not performed a COPAS-based quantification. And conversely, when qRT-PCR analyses are clear cut, we have not always added a third repeat; in those cases, p values have no real statistical value and have been omitted.

There seems to be lots of worm genes and worm jargon presented to the reader, and I wonder how well the non-expert will follow everything. Could schematics be added to some figures better outlining the signaling pathways/target genes being tested when the specific data are presented. Similarly, the authors don't really explain (at least not in sufficient detail) what nipi, nlp, cnc genes are. Likewise, wherever possible, I would use clearer transgene nomenclature - ie refer in the text and legends to nlp-29::gfp, not frIs7, so the reader doesn't have to look up the strains while reading the results.

We have modified the text (while adhering to the strict *C. elegans* nomenclature rules) and added schematics as suggested.

While DcEntC expression did not affect lifespan, it would be interesting to test if DcENtC does in fact have an effect in the presence of fungus; the authors could be missing something interesting here.

Although the reviewer raises an interesting point, we think that adding the results of such an analysis of DcEntC would add unwarranted complexity to the current study. With the agreement of the editor, we have not conducted these tests, but have mentioned the possibility in the revised version.

1. The plasmid construction section in the Methods could use more details. I might start by adding a reference to Fig. 1 and explaining what all the components being assembled are and what they do. I would also present all the aliases for the three Ent genes one time and then stick with a single nomenclature thereafter (for example, the nomenclature in the Methods, in Fig. 1, and in Table S2 all differ).

We have implemented the suggested changes.

2. At the start of the Results, the authors describe elegant approaches to engineer expression of g2698 without developmental lethality, but the manuscript then shifts gears and is focused on other enterotoxins (that do not use this methodology to modulate their expression). I gather that this reflects the history of why expression of these 3 toxins was engineered in this way, but it is distracting since the manuscript shifts gears completely between the first section and the rest of the results. It might be less distracting to touch on this more briefly and leave the details in the methods. Is this approach described in another manuscript that could be referenced?

We agree that this part, which was under-developed and not connected with the rest of the manuscript, does not add significantly to the study. It has also been drawn to our attention that it could be considered close to “dual-use” material. As we have substantially expanded both the cell biology and the investigation of translational inhibition, we have removed this part of the Results.

3. The authors state that they did not use auxin to control enterotoxin levels. Can they also clarify in the text if they used RNAi to further inhibit developmental expression of these enterotoxins?

This has been clarified.

4. Is F40H7.12 expression regulated by D. coniospora?

In the light of this comment and minor comments from the other 2 reviewers, we have now produced a short article about F40H7.12/*ifas-1* and refer to it in the revised MS. We have also added data and discussion regarding its induction as suggested.

5. The data for Fig. 5D shows one rep; the other two reps are in Figure S5. It's odd that these data weren't combined, I assume because of the markedly different ranges. Since these data represent large gene expression changes, could these data be combined (and statistical analysis performed) if they were plotted on a log2 scale? At a minimum, the choice to present these data in this way should be commented on. Same thoughts about the three replicates presented separately in Fig. S4.

We have re-organised the data as suggested.

6. The authors need to clarify that the GSEA analysis in the DcEntA section is focused on the DcEntA interactors; this isn't stated in the Results section.

This has been clarified.

7. There is lots of Discussion present in the Results sections dealing with the protein interaction studies. These should be shortened and moved to the Discussion. Similarly, the Discussion section is overly long and should be more focused.

In the light of the other reviewers’ comments, and with editorial approval, we have strengthened the results related to the protein interactors through cell biological analyses and, in parallel, trimmed down the more speculative sections, based only on *in silico* analyses.

8. The mention of mass spec on four other worm strains and the reference to an in preparation manuscript that is not in the references should be deleted; I don't think this is relevant here.

As mentioned above, the results for the other strains were used to define the specific protein interactors. All the data is publicly available. This has been clarified.

9. I would add a key to Fig. 1B so it is clear what domains are present without having to refer back to the legend. The light blue domain is not defined.

We have made the suggested modifications.

10. Table S3 was not converted to a PDF correctly.

This has been rectified.
